# Exploration of binding site pattern in arachidonic acid metabolizing enzymes, Cyclooxygenases and Lipoxygenases

**DOI:** 10.1186/s13104-015-1101-4

**Published:** 2015-04-16

**Authors:** Kakularam Kumar Reddy, Veena Kumari Vidya Rajan, Ashish Gupta, Polamarasetty Aparoy, Pallu Reddanna

**Affiliations:** School of Life Sciences, University of Hyderabad, Hyderabad, 500 046 India; Sastra University, Tirumalaisamudram, Thanjavur, 613401 India; Centre for Computational Biology and Bioinformatics, School of Life Sciences, Central University of Himachal Pradesh, Dharamshala, 176215 India; National Institute of Animal Biotechnology, Hyderabad, 500049 India

**Keywords:** Cyclooxygenase, Lipoxygenase, Arachidonic acid, Specific inhibitors, Receptor based pharmacophore, Drug design

## Abstract

**Background:**

Cyclooxygenase (COXs) and Lipoxygenase (LOXs) pathways are the two major enzymatic pathways in arachidonic acid (AA) metabolism. The term eicosanoid is used to describe biologically active lipid mediators including prostaglandins, thromboxanes, leukotrienes and other oxygenated derivatives, which are produced primarily from AA. Eicosanoids generated in a tissue specific manner play a key role in inflammation and cancer. As AA is the substrate common to variety of COXs and LOXs, inhibition of one pathway results in diversion of the substrate to other pathways, which often is responsible for undesirable side effects. Hence there is need for development of not only isozyme specific inhibitors but also dual/multi enzyme inhibitors. Understanding the interactions of AA and characterizing its binding sites in these enzymes therefore is crucial for developing enzyme specific and multi enzyme inhibitors for enhancing therapeutic efficacy and/or overcoming side effects.

**Results:**

AA binding sites in COXs and LOXs are identified and compared by the development of receptor based pharmacophore using MultiBind. Physico chemical properties were compared to understand the details of the binding sites in all the enzymes and to elucidate important amino acids that can be targeted for drug design. The alignment of AA binding sites in the seven enzymes COX-1, COX-2, 5-LOX, 12-LOX, 15-LOX and plant soybean LOX-1 and LOX-3 indicated a common pattern of five common interacting groups. In the same way, comparison of AA binding sites was done pair wise and by multiple alignment in various combinations. It has been identified that aliphatic and aromatic interactions are the most common in all the enzymes. In addition interactions unique to each one of these enzymes were identified.

**Conclusion:**

The complete analysis of AA binding sites in the seven enzymes was performed; 120 combinations for the seven enzymes were studied in detail. All the seven enzymes are structurally quite different, yet they share AA as the common binding partner. Comparisons in various combinations showed how they are similar and dissimilar with each other. This information will be helpful in designing specific as well as common inhibitors.

**Electronic supplementary material:**

The online version of this article (doi:10.1186/s13104-015-1101-4) contains supplementary material, which is available to authorized users.

## Background

Arachidonic acid (AA), the major polyunsaturated fatty acid (PUFA) present in mammalian systems, is oxygenated by three important pathways – the cyclooxygenase (COX), the lipoxygenase (LOX) and the epoxygenase, to form biologically active molecules such as prostaglandins (PGs), leukotrienes (LTs), and epoxyeicosatrienoic acids (EETs), collectively called as eicosanoids [1].

COX is a bifunctional heme containing enzyme that catalyzes the biosynthesis of PGs from AA. It is bifunctional enzyme and exhibits cyclooxygenase and peroxidase activities. It introduces two molecules of oxygen into AA to form PGG_2_, a cyclic hydroperoxy endoperoxide, which is subsequently reduced by peroxidase to give hydroxy endoperoxide, PGH_2_ [2]. There are three COX isoforms, COX-1, COX-2, and COX-3 [1-3]. COX-1, constitutively expressed in most tissues synthesizes PGs at low levels, and is presumed to function primarily in the maintenance of physiological functions [4-7]. The inducible isoform of COX, COX-2 is induced by several and plays a direct role in inflammation, cancer and various other diseases [8,9]. COX-3, a product of COX-1 gene has been identified as the alternatively spliced form found mostly in the brain.

The other important group of enzymes involved in AA metabolism, LOXs, are closely related non-heme iron containing dioxygenases which catalyze the addition of molecular oxygen to form hydroperoxy metabolites (HPETEs). LOXs are broadly classified as 5-, 12, and 15-LOXs in animals [10,11] based on regio and stereo specific incorporation of molecular oxygen on AA. In plants, LOXs are classified as 9- and 13-LOXs based on the regio specific incorporation of oxygen on Linoleic (LA) or α–Linolenic acids [12]. Eicosanoids generated by these LOXs in a tissue specific manner play key role in various diseases. In various LOX isoforms the active sites have modifications to effect specific reactions but the basic enzyme structures are conserved [13-16]. Hence it is very important to identify important conserved/non-conserved amino acids at the active sites of these LOXs.

Non steroidal anti-inflammatory drugs (NSAIDs) are the most commonly used remedies for arthritis and some inflammation associated diseases. A most promising approach seemed to be the preparation of novel NSAIDs specific for the COX-2 (COXIBs) which does not inhibit COX-1 effectively hence devoid of gastrointestinal toxicity associated with non-specific COX-1/COX-2 inhibitors. These COXIBs, however were shown to have cardiac side effects when used at high concentrations for a long period. These side effects have been attributed to the shift towards LOX pathway, hence an alternative approach of developing COX-2/5-LOX dual inhibitors (CLOXIBs) is recently being considered [17]. Licofelone, a COX-2/5-LOX dual inhibitor has successfully completed phase III trials and is demonstrated to be safe and efficacious for standard treatments of osteoarthritis [18].

Development of specific inhibitors or dual inhibitors of enzymes involved in AA metabolism is of great challenge. Binding of the same substrate to LOX and COX enzymes implies that there is a common microenvironment within the catalytic sites of these enzymes, hence designing an inhibitor which interacts specifically with one/two enzymes requires a detailed understanding of binding sites of the substrate AA to these enzymes. The main objective of this study is to compare binding site features in AA metabolizing enzymes and to elucidate important amino acids that can be targeted for drug design. In this study five isoforms of LOX and two isoforms of COX enzymes (to generate reliable data enzymes having crystal data alone were considered) collectively were considered for the analysis. Even though there are COX-substrate structures available in Protein Data Bank (PDB), till date there is no crystal structure for LOX-substrate complex. Structural aspects of these enzymes were understood using receptor based pharmacophore models and by comparison of physicochemical properties at binding site were performed. The present study will provide insights into AA binding sites of these enzymes and may form the basis for developing enzyme specific as well as dual/multi enzyme targeting novel drugs. Multitarget drug design is gaining prominence in modern drug discovery and in silico approaches may play a very crucial role in such approaches [19]. In this context this study will aid development of more potential inhibitors of AA metabolizing enzymes.

## Results

### Pair wise alignments

The hydrophobic nature of AA substrate suggests that predominantly hydrophobic residues line the substrate-binding pocket. To understand the AA binding sites of LOXs in comparison with COXs, pair wise alignment studies were performed. The binding sites were initially compared in a pair wise manner. Pair wise surface alignments of the enzymes detect more common features and help in the elucidation of important amino acid similarities between members of same class and also individually with enzymes of another family.

**Figure 1:**
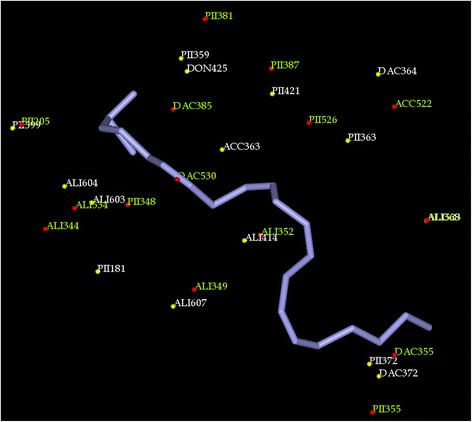
Common physiochemical parameters identified for 5-LOX (white) and COX-2 (green). ALI, PII, ACC, DON, DAC interacting groups at the AA binding site are labeled.
  In comparison, AA binding sites of 12-LOX and 15-LOX were scored 32.68 and 41.89 respectively with COX-2. The 10 common features, detected in pairwise alignment of COX-2/12-LOX, have been dominated by seven ALI features. Only one PII contact was observed different from the case of COX-2/COX-1 and COX-2/5-LOX. Unlike COX binding sites where different amino acids (Val/Ile) share same ALI feature, the COX-2 and 12-LOX share 2 similar features for different amino acids. Histidine in 12-LOX contributes DAC group wherein Serine of COX-2 possesses ACC group; in another feature, Histidine in 12-LOX contributes DAC group wherein Glutamine in COX-2 supplies ACC property. It is significant that 12-LOX has 2 additional donor properties compared to COX-2 due to the presence of two DAC groups complimentary to the ACC groups of COX-2.
  The alignment between the AA binding sites of 15**-**LOX and COX-2 revealed that all amino acids are different, sharing the same property except one. Glycine possesses same property in both enzymes. The amino acids contributing for the same physicochemical properties are shown in Additional file 1. As can be seen from the Table 1, the binding site of 15-LOX shares more similarity with COX-2 than the other two LOXs based on the score. But interestingly, 5-LOX has more common features with COX-2 than the other LOXs.

1. Comparison of the COXs : COX-1 – COX-2
  COX, one of the pathways in AA metabolism is of great therapeutic interest and a simple pubmed search using the keyword ‘COX inhibitor’ retrieves around 110000 articles showing the quantum of research being carried out on these enzymes. As mentioned earlier, COX-1 and COX-2 are members of the same family and they share large similarity with each other in their enzyme reaction with their common substrate AA. Pairwise alignments of the binding sites in MultiBind showed that they have great similarity at the AA binding site. They were scored 90.4106 with 36 similar features. The most common features observed were aliphatic (ALI) and aromatic pi contacts (PII). Apart from these, common hydrogen bond donor (DON), hydrogen bond acceptor (ACC) and mixed hydrogen bond donor and acceptor (e.g. in histidine) (DAC) were also observed. The common physico-chemical properties and the contributing amino acids are shown in Additional file 1: Table S1. There are also different amino acids in COX-1 and COX-2 sharing the same ALI feature. Isoleucine in COX-1 is replaced with Valine in COX-2. Ile/Val are two important amino acids forming ALI site points at the binding site of the COXs, the size difference in these amino acids, resulting in volume differences at the active centre, is the only salient distinction observed. This size difference has been exploited successfully in designing COXIBs. Celecoxib shows 375-fold selectivity towards COX-2 over COX-1 [20]. Studies showed that the primary factor contributing to the COX-2 selectivity of celecoxib and related 1,5-diarylpyrazoles is the substitution of Ile523 in COX-1 for Valine in COX-2 [21]. This shows that the differences and the common features in arachidonic binding site with each one of these enzymes can be exploited in the development of selective as well as dual/multi enzyme inhibitors.
2. Comparison of COX-2 with Animal LOXs
  Despite the relatively safe pharmacological profile of the COXIBs like celecoxib and valdecoxib there is concern regarding their use in patients with emerging news of myocardial infarction on prolonged use. This is due to the shift of the AA towards LOX pathway because of inhibition of COX pathway. Therefore, there is increasing need for development of anti-inflammatory drugs with fewer/no side effects. In this connection, various research groups started working on dual COX-2/LOX inhibitors (CLOXIBs) that would prevent the drift of AA towards the other pathway [22]. In order to design CLOXIBs the information of AA binding sites in these enzymes is very useful. The main focus of the study was identify common features of COX-2 binding site with 5-LOX, 12-LOX and 15-LOX individually. The patterns or important interactions observed will further aid in planning drug design experiments.
  The alignment of AA binding sites of COX-2 and 5-LOX produced a score of 33.43 in MultiBind with 15 common physicochemical properties. Common ALI, PII, ACC and DAC interactions were observed. Three amino acids Tyr, Phe and Leu are commonly observed in AA binding site of both the enzymes. PII interactions are more in number followed by ALI. ACC and DAC features were observed, the ACC of Gln363 in 5-LOX is comparable to Ser530 of COX-2 and DAC of His372 is comparable to Tyr355. Though the hydrogen bond forming functional groups are not of the same amino acids, they can be helpful in the design of common inhibitors. The complete list of amino acids and the common physicochemical features are included in Additional file 1: Table S2 and shown in Figure 1.

**Table 1:** **MultiBind data on pairwise alignments of COX-2 binding site with all six enzymes**

**Compared proteins**	**No. of detected features**	**Score**
COX-2 – COX-1	36	90.4106
COX-2 – 5-LOX	15	33.4303
COX-2 – 12-LOX	10	32.6815
COX-2 – 15-LOX	12	41.8927
COX-2 – LOX-1	8	19.6051
COX-2 – LOX-3	11	35.3228

**Table 2:** **Pair wise alignments of 5-LOX binding site with other LOXs using MultiBind**

**Compared proteins**	**No. of detected features**	**Score**
5-LOX– 12-LOX	10	35.3112
5-LOX– 15-LOX	14	41.9221
5-LOX– LOX-1	8	24.9893
5-LOX– LOX-3	15	36.8885
3. Comparison of COX-2 with Plant LOXs
  The two plant LOXs, soybean LOX-3 and soybean LOX-1 had 11 and 8 common features with AA binding site of COX-2 (Table 1). They are aligned with scores 35.3228 and 19.6051 respectively. It can be seen that animal LOXs are more similar to the inducible COX-2 enzyme than plant LOXs.
4. Comparison of 5-LOX with other animal LOXs
  The binding site of 5-LOX and 12-LOX have five ALI features in common out of the ten common physicochemical properties identified, indicating it to be the most prominent property in the binding site. Two PII interactions are also observed. Interestingly charged amino acid Glu356 of 12-LOX is present corresponding to Gln363 of 5-LOX. Both of them act as ACC. The other prominent amino acid differences are shown in Additional file 1. The 12-LOX was scored 35.31 when compared to 15-LOX which had similarity score of 41.92 with 5-LOX as shown in Table 2. Both 12 and 15-LOX had seven ALI and three PII features in common, the replacement of charged amino acid Glu to Gln is also seen in 15-LOX. The high score of 15-LOX with 5-LOX is because of the presence of seven identical amino acids contributing for common interacting groups.
5. Comparison of 5-LOX with plant LOXs
  In the two plant LOXs considered, Soybean LOX-3 showed 15 common features and Soybean LOX-1 showed 8 common features with the binding site of 5-LOX. They are aligned with scores 36.8885 and 24.9893 respectively. Based on this comparison, it can be seen from the similarity score that AA binding sites in plant LOXs share almost the same similarity with animal 5-LOX and COX-2. Among the two plant LOXs, LOX-3 has higher similarity with 5-LOX and COX-2.
6. Comparison of COX-1 with all LOXs
  Due to the high similarity between COX-2 and COX-1, development of COX-2 inhibitors with less affinity towards COX-1 is a big challenge. The binding site of COX-1 was compared with that of the LOXs to recognise the structurally conserved physicochemical patterns. The animal LOXs, 5-LOX, 12-LOX and 15-LOX showed 14, 10 and 11 common features with scores of 33.04, 31.34 and 31.45 respectively. Soybean LOX-3 and soybean LOX-1 had 12 and 9 common features with scores of 36.54 and 23.20 respectively. The studies showed that the binding sites of COX-1 have less similarity with LOXs when compared with COX-2. COX-1 has highest similarity with soybean LOX-1 followed by 5-LOX whereas COX-2 has maximum similarity with 15-LOX.

### Multiple alignments

Multiple alignment of 3–7 binding sites were performed. The physicochemical similarities observed in various combinations are included in Additional file 1 and some of the alignments are discussed below:

1. All animal LOXs
  The multiple surface alignment of all the three binding sites of animal LOXs indicated a common pattern of eight physiochemical properties, namely one DAC, four ALI interactions, two ACC and one PII contacts. Figure 2 lists the common physiochemical parameters identified for each of the three binding sites. ALI interactions are more in number followed by ACC.

**Figure 2:**
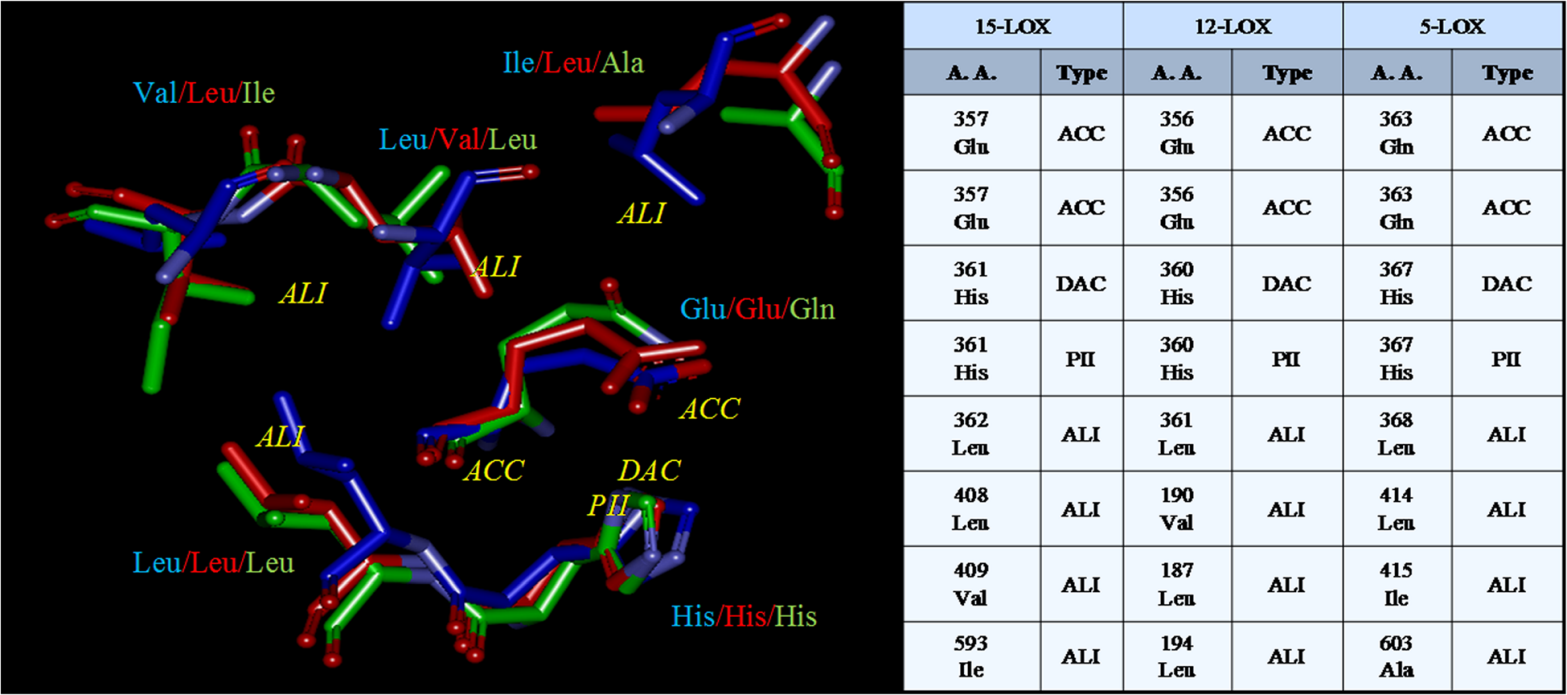
Common physiochemical parameters identified for 5-LOX, 12-LOX and 15-LOX. The amino acids of 5-LOX (green), 12-LOX (red) and 15-LOX (blue) contributing to the common properties are shown.
2. COX-1, COX-2 and individual LOXs
  The comparison of the COXs with individual human LOXs was performed. It has been observed that the number of features range from 6–13. 5-LOX has 13 common physicochemical parameters with COXs where as sLOX-1 has only 6 common features. Human LOXs were scored higher than plant LOXs as seen in Table 3.

**Table 3:** **Alignments of individual LOXs with COXs using MultiBind**

**S. no**	**Compared proteins**	**No. of detected features**	**Score**
1	COX1-COX2-sLOX3	10	40.4966
2	sLOX1-COX1-COX2	6	32.5165
3	15LOX-COX1-COX2	10	47.1464
4	12LOX-COX1-COX2	10	40.7187
5	5LOX-COX1-COX2	13	45.4904
3. COX-1, COX-2 and all human LOXs
  Alignment of the five binding sites of COXs and animal LOXs indicated a common pattern of five physicochemical properties. ALI interactions are more in number in this multiple alignment. The details are enclosed in Table 4.

**Table 4:** **Common physiochemical parameters identified for COX-1, COX-2, 5-LOX, 12-LOX and 15-LOX**

**15-LOX**		**12-LOX**		**5-LOX**		**COX-1**		**COX-2**	
**A. A.**	**Type**	**A. A.**	**Type**	**A. A.**	**Type**	**A. A.**	**Type**	**A. A.**	**Type**
Glu 357	ACC	Glu 356	ACC	Gln 363	ACC	Ser 530	DAC	Ser 530	DAC
His 361	DAC	His 360	DAC	His 367	DAC	Ala 527	DON	Ala 527	DON
His 361	PII	His 360	PII	His 367	PII	Gly 526	PII	Gly 526	PII
Leu 362	ALI	Leu 361	ALI	Leu 368	ALI	Met 522	ALI	Met 522	ALI
Leu 408	ALI	Val190	ALI	Ile 415	ALI	Leu 352	ALI	Leu 352	ALI
4. All 7
  Alignment of all the seven binding sites resulted in a common pattern with five physicochemical properties as shown in Figure 3, namely two ALI, one PII and two ACC. The score obtained when aligned was 21.0266.

**Figure 3:**
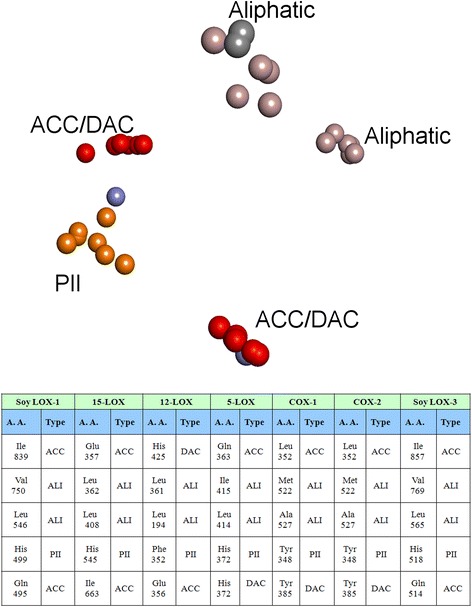
Licofelone in the binding site of **A)** COX-2 and **B)** 5-LOX.

### Validation of COX-2/5-LOX and 5-LOX/15-LOX model generated with docking studies

**Figure 4:**
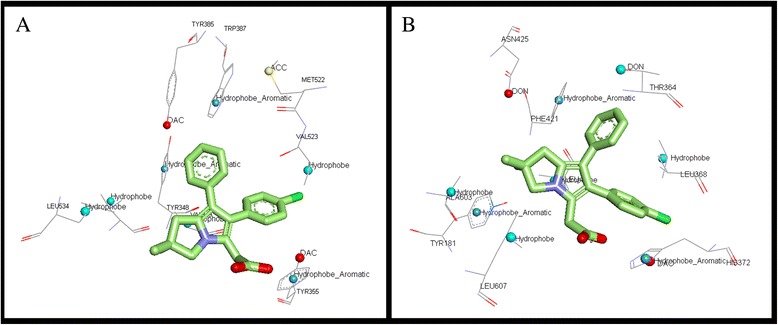
Receptor based pharmacophore of all the LOXs and COXs was studied. Aliphatic groups are shown in grey; Acceptor groups are shown in red; Donor/Acceptor groups are shown in blue; Aromatic pi contacts are shown in orange.

COX-2/5-LOX model generated was further validated by docking studies with a known COX-2/5-LOX dual inhibitor, licofelone. As shown in Figure 4, the compound formed interactions with amino acids that were found to be important. The receptor models generated showed that the amino acids of both the proteins 5-LOX and COX-2 have common interacting groups at the active site. Docking results supplemented the models. The only charge group, the carboxyl moiety of licofelone interacted with common DAC feature (Tyr 355 in COX-2 and His372 in 5-LOX). The hydrophobic Cl group aligned at the common ALI feature (Val 523 in COX-2 and Leu 368 in 5-LOX). The aromatic ring of licofelone formed strong PII interactions with Trp 387 in COX-2 and Phe 421 in 5-LOX, in correlation with the common PII feature observed in the model. The dimethylcyclopentane moiety formed hydrophobic interactions with few common ALI features observed. Hence, the docking results showed that the model generated can be used to elucidate the common features and can be further used in the design of dual inhibitors. There is a common hydrogen bond feature close to the vicinity of licofelone corresponding to Tyr 385 in COX-2 and Thr 364 in 5-LOX. This site point can be further used for lead optimization employing site point connection method.

**Figure 5:**
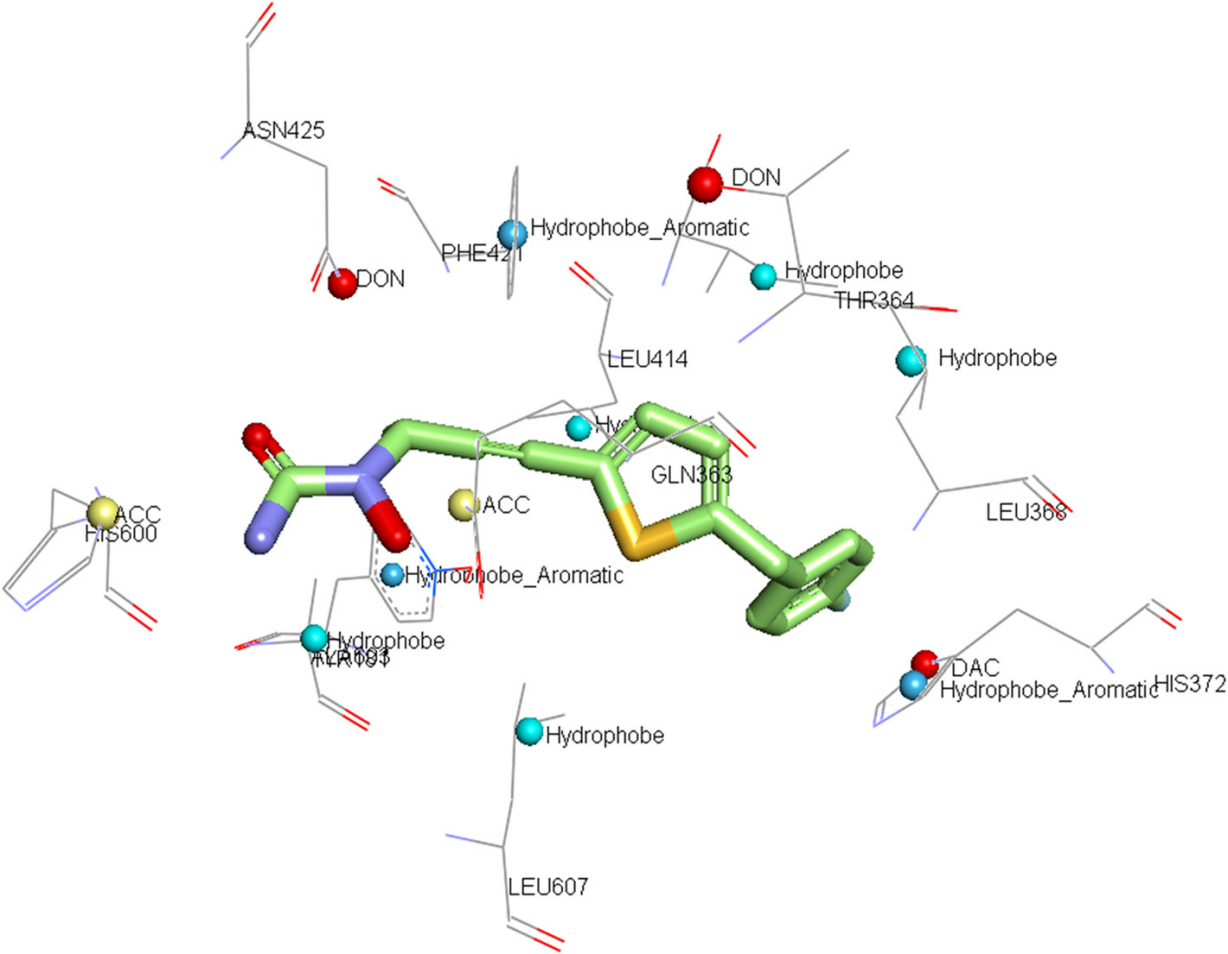
ABT-761 in the binding site of 5-LOX.

As discussed earlier, the 5-LOX and 15-LOX model generated showed similar features at the binding site. One of the binding features, ACC was contributed by two different amino acids in both the enzymes, Glu in 15-LOX and Gln in 5-LOX respectively. Hence, it can be mentioned that a functional group which interacts with amide group of 5-LOX and repels with carboxylic group in 15-LOX can be used to develop specific 5-LOX inhibitor. To support this docking was performed with ABT-761, a specific 5-LOX inhibitor. ABT-761 was docked into the binding site of 5-LOX and 15-LOX and its interactions were studied. As shown in the Figure 5, ABT-761 aligns in the center of the binding site. The hydroxyl group of hydroxyurea formed strong hydrogen bond with the amide group of Gln 363. In 15-LOX, the inhibitor did not bind at the binding site this may be due to repulsion between carboxyl group of Glu 357 and the inhibitor.

## Discussion

The goal of drug discovery is to design exquisitely selective ligands that act on a single disease target. The current approach is that safer, more effective drugs will result from designing very selective ligands where undesirable and potential side effects have been removed [19]. Multitarget strategies have also gained importance in the recent time. To design a specific or multitarget inhibitor it is very important to know the binding site patterns in closely related pathways. In a recent study, Doble et al. have done a comparative study of PGH_2_ binding site in prostaglandin synthases [23]. Such studies would provide insights into the chemical features which can be exploited for selective/ multitemplate inhibitor design.

In the present study we aimed to understand the physicochemical features unique or similar in AA metabolizing enzymes. It has been identified that in the COXs there are 36 common features at the AA binding site, the only difference at the binding sites was Val substitution in COX-2 for Ile in COX-1 at position 523. Previous studies suggested that this small change in amino acid provides additional space in COX-2 binding pocket. The sulfonamoylphenyl or methylsulfonylphenyl moieties of COXIBs can therefore be accommodated only in the active site of COX-2. This supports that amino acid differences at the common physicochemical parameters studied can contribute for the development of specific/selective inhibitors.

The comparative study of human 5-LOX, 12-LOX and 15-LOX has shown that there are 8 common features, two common ACC, one DAC, one PI and four ALI interaction groups. This is in correlation with the previous reports on ligand based pharmacophore model of 5-LOX developed by Aparoy et al. [14] and Charlier et al. [24] where the significance of these four types of interactions has been explained. The results obtained in this study on multiple alignments of the binding sites reveal that soybean LOX-1 is less similar with other binding sites in other enzymes and its inclusion reduces the score in all the alignments. COXs, however, have very common interacting groups at the AA binding site. Human LOXs have 10–15 common interacting groups. 5-LOX has maximum similarity score of 41.92 with 15-LOX and has 14 common features. In the pair wise comparison of COX-2 with all LOXs individually, it has been observed that COX-2 is more similar to human LOXs than the plant isoforms. The benefits of developing CLOXIBs have been discussed earlier. To develop a COX-2 inhibitor which would also bind to 5-LOX specifically is a great challenge. The pairwise studies of all human LOXs individually with COX-2 showed that 15-LOX has maximum complimentary score followed by 5-LOX. COX-2/5-LOX has 15 common features compared to 12 common features in COX-2/15-LOX. The less similarity of COX-2 and 5-LOX at binding site and almost equivalent features among COX-2/5-LOX and COX-2/15-LOX makes the design of dual inhibitors concept more challenging. In our study, which is aimed to provide insights into development of specific dual inhibitors, various important differences/similarities at binding sites have been identified. COX-2/5-LOX pharmacophore model showed 15 common interactions when compared to 12 interactions in COX-2/15-LOX and 10 interactions in COX-2/12-LOX. COX-2/5-LOX model has more PI interactions (6) in common followed by ALI (5). The COX-2/15-LOX and COX-2/12-LOX models have fewer PI interactions (1 and 2 respectively), hence the amino acids at 5-LOX binding site Tyr181, Phe359, Phe421, Trp599 can be targeted for aromatic interactions which may increase specificity towards COX-2/5-LOX.

The differences observed in the AA binding site of the LOXs could be exploited for the development of potential dual/multi inhibitors. A major difference that has been observed in the receptor based pharmacophore models of the human LOXs is that, amino acid Glu with acidic side chain is replaced with Gln in 5-LOX. Rational design of compounds which would interact effectively with Gln than Glu can form potential specific 5-LOX inhibitors. Even bulkier amino acid Ile 593 in 15-LOX is replaced with smaller amino acid Ala in 5-LOX. A bulky hydrophobic group in the ligand can occupy the corresponding space in 5-LOX. These differences can be taken advantage of in the development of specific 5-LOX inhibitors as in the case of COX-2 inhibitors.

## Conclusions

The complete analysis of AA binding sites in the seven enzymes was performed; 120 combinations for the seven enzymes were studied in detail. All the seven enzymes are structurally quite different, yet they share AA as the common binding partner. Our comparative study shows that there is no precise or specific pattern observed at AA binding sites of the enzymes studied. It has been observed that the most important features observed at the active site (ALI and PII interactions) are in agreement with the previous reports. Individually, when enzymes were compared to each other, many common features have been observed which can be exploited for design of a molecule which can bind effectively at the interaction points identified and form common class of inhibitors. In this study, differences were also observed at amino acid level i.e. two different amino acids present in two different enzymes share the same physicochemical property. This can be exploited in specific drug design, as was done earlier in the case of COX-2.

## Methods

### Preparation of enzyme-substrate complexes

In this study, AA metabolizing enzymes LOXs and COXs were considered collectively for binding site analysis. Two COX enzymes namely, COX-1 (PDB id. 1DIY) [25] and COX-2 (PDB id. 3HS5) [26] co-crystallised with AA were obtained from PDB (www.rcsb.org/pdb). To date structural information of only five LOXs are available: two isoforms from soybean, LOX-1 (PDB id. 1YGE) [27] and LOX-3 (PDB id. 1IK3) [28]; three animal LOX isoforms rabbit reticulocyte 15-LOX (PDB id. 1LOX) [29] and human 5-LOX (PDB id. 3O8Y) [30] and 12-LOX (PDB id. 3D3L). All the LOX crystal structures were not co-crystallized with the substrate, hence to obtain protein-AA complex docking was performed.

GOLD (Genetic Optimization of Ligand Docking), a docking program based on genetic algorithm [31] was used to dock the substrate i.e. AA. The structure of AA was sketched and minimized using cerius2 and structure with cis double bonds is obtained in conformational analysis. During docking the default algorithm speed was selected. The number of poses for each inhibitor was set to 100 and early termination was allowed if the top three bound conformations of a ligand were within 1.5 Å RMSD. Input parameters of the GOLD were set to allow octahedral coordination geometry to iron. After docking, the individual binding poses of each ligand were observed and their interactions with the protein were studied. The best and the most energetically favorable conformation of each ligand was selected. A four-stage protocol was set up for energy minimizations of the protein-inhibitor complex [15]. Minimization at each stage was performed using 100 steps of steepest descent and 1500 steps of conjugate gradient algorithms for minimization. In the first stage, water molecules were minimized keeping the inhibitor and protein atoms fixed. This will relieve any bad contacts involving water molecules in the initially solvated system. In the second stage the hydrogen atoms of the whole system were allowed to relax. This step relaxes the hydrogen atoms prior to relaxing heavy atoms. In the third stage all the atoms of the inhibitor and the solvent are allowed to move during optimization. This stage establishes the preferred interactions. In the fourth and final stage, all the protein atoms within 15 Å from the center of the inhibitor (water molecules, protein atoms and the ligand) were allowed to relax. After optimization of the protein-ligand complexes, they were visualized and studied using InsightII.

### Comparative analysis of binding sites using MultiBind

After obtaining the LOX-AA complexes, to compare the binding pockets and to determine the common features which facilitate the binding of the same substrate, the program MultiBind was used [32]. MultiBind reveals the common physico-chemical patterns (receptor based pharmacophore) that may be responsible for the binding of a small molecule in a set of binding sites. Molecular surface of the binding site is determined by solvent accessible surface points that are located from the surface of the bound molecule [33]. The program further performs multiple alignments of binding sites and recognizes conserved physicochemical and geometrical patterns directing the common binding. MultiBind develops receptor based pharmacophore model for the proteins of interest. Each amino acid in a binding site is represented by points in 3D space termed pseudocenters as described by Schmitt et al. [34]. Each pseudocenter represents one of the following physicochemical properties important for protein–ligand interactions: hydrophobic, ALI, PII, DON, ACC and DAC. It will superimpose the binding sites in a manner that will maximize the physicochemical score of the matched properties. High MultiBind score implies high similarities in the physicochemical properties of the binding sites of the proteins compared.

First, the binding sites were compared pair wise. The COX enzymes, COX-1 and COX-2 were compared with each other. Further, AA binding site of COX-2 was compared with all the LOXs. Later multiple binding site studies were performed; comparison of 6 enzymes eliminating one enzyme at a time from the MultiBind during run time. There were six different combinations possible in this stage. Later the alignment between 5 binding sites was performed. This study had 21 possible combinations. In the same way combinations of four, three and two enzyme binding sites were also performed.

### Verification of COX-2/5-LOX and 5-LOX/15-LOX models generated

Prior to the application of the developed models for drug design, validation of the same is very important. The dual inhibitors of COX-2/5-LOX design have great therapeutic importance and hence the common receptor based models obtained for COX-2/5-LOX were further validated by docking studies. A COX-2/5-LOX dual inhibitor, Licofelone was used for docking studies. If the receptor based pharmacophore model generated for COX-2/5-LOX is accurate, Licofelone should form interactions with the common physico chemical features identified. ABT-761, a specific 5-LOX inhibitor was also docked and its interactions with 5-LOX and 15-LOX were compared to the models obtained. The molecules were docked using the methodology explained earlier and their interactions were investigated and visualized using Accelrys Discovery Studio Visualiser 3.0.

## Additional file

Additional file 1:
**Exploration of binding site pattern in arachidonic acid metabolizing enzymes, lipoxygenases and cycloxygenases.**
