# Supplementary material for: Exploration of binding site pattern in arachidonic acid metabolizing enzymes, Cyclooxygenases and Lipoxygenases
Source: BMC Res Notes. 2015 Apr 16;8:152. doi: 10.1186/s13104-015-1101-4 (PMC4416244; doi:10.1186/s13104-015-1101-4)

**EXPLORATION OF BINDING SITE PATTERN IN ARACHIDONIC ACID METABOLIZING ENZYMES, LIPOXYGENASES AND CYCLOXYGENASES**

**
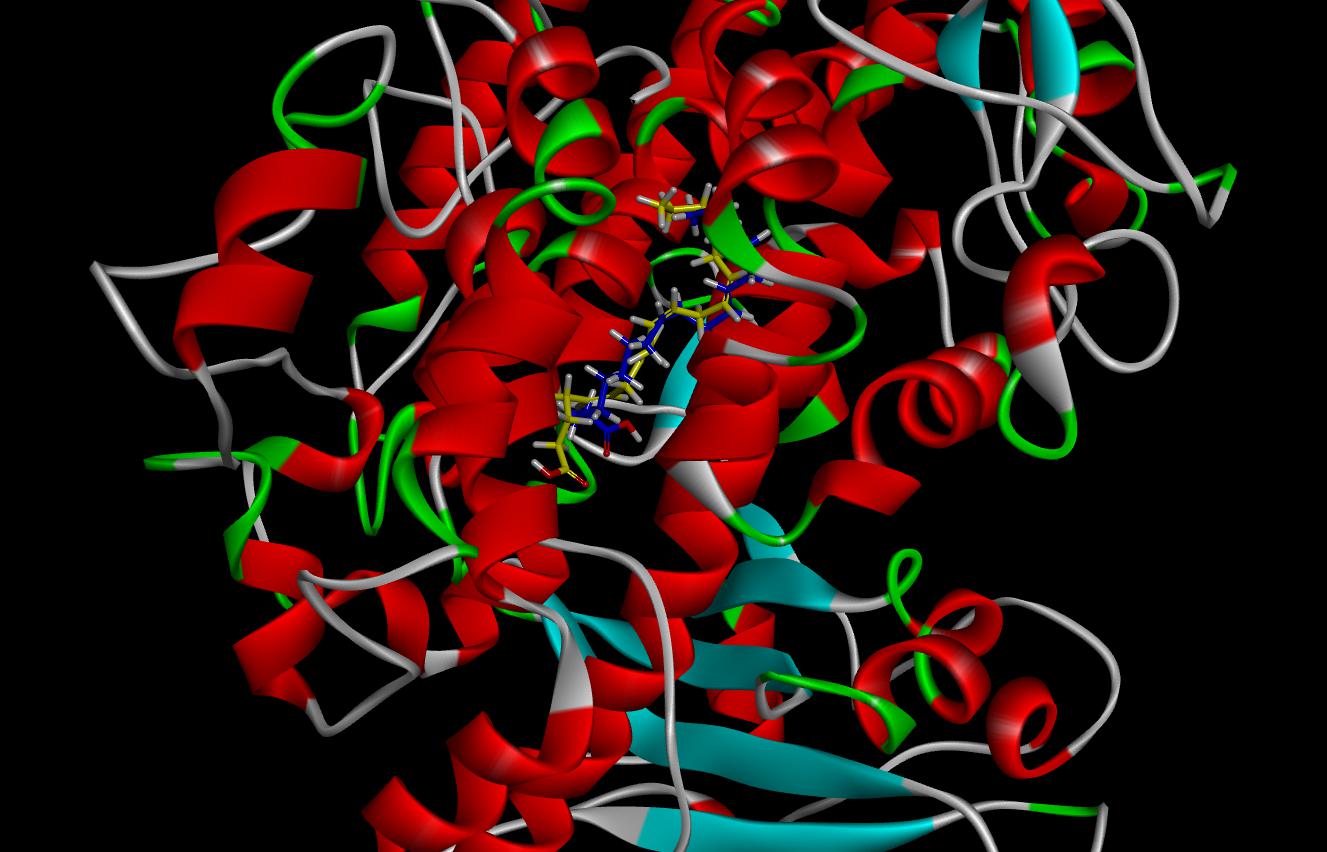
**

**Figure S1:** Docked complex of LA (yellow) and AA (blue) in the active site of soybean LOX-3

| Site 1:Soybeans LOX-1 | | | Site 2: COX2 | | |
| --- | --- | --- | --- | --- | --- |
| **Chain.ID** | **A. A.** | **Type** | **Chain.ID** | **A. A.** | **Type** |
| A.259 | Thr | ALI | A.535 | Met | ALI |
| A.499 | His | PII | A.381 | Phe | PII |
| A.500 | Trp | PII | A.348 | Tyr | PII |
| A.541 | Leu | ALI | A.113 | Met | ALI |
| A.546 | Leu | ALI | A.534 | Leu | ALI |
| A.547 | Ile | ALI | A.344 | Val | ALI |
| A.553 | Ile | ALI | A.228 | Val | ALI |
| A.839 | Ile | ACC | A.527 | Ala | ACC |

| Site 1: COX2 | | | Site 2: Soybeans LOX-3 | | |
| --- | --- | --- | --- | --- | --- |
| **Chain.ID** | **A. A.** | **Type** | **Chain.ID** | **A. A.** | **Type** |
| A.93 | Leu | ALI | A.726 | Arg | ALI |
| A.116 | Val | ALI | A.728 | Thr | ALI |
| A.345 | Ile | ALI | A.769 | Val | ALI |
| A.349 | Val | ALI | A.773 | Leu | ALI |
| A.359 | Leu | ALI | A.770 | Ile | ALI |
| A.381 | Phe | PII | A.519 | Trp | PII |
| A.385 | Tyr | PII | A.523 | His | PII |
| A.527 | Ala | ACC | A.514 | Gln | ACC |
| A.531 | Leu | ALI | A.572 | Ile | ALI |
| A.534 | Leu | ALI | A.565 | Leu | ALI |
| A.535 | Met | ALI | A.571 | Val | ALI |

| Site 1: 5-LOX | | | Site 2: COX2 | | |
| --- | --- | --- | --- | --- | --- |
| **Chain.ID** | **A. A.** | **Type** | **Chain.ID** | **A. A.** | **Type** |
| B.181 | Tyr | PII | A.348 | Tyr | PII |
| B.359 | Phe | PII | A.381 | Phe | PII |
| B.363 | Gln | PII | A.526 | Gly | PII |
| B.363 | Gln | ACC | A.530 | Ser | DAC |
| B.364 | Thr | DAC | A.522 | Met | ACC |
| B.368 | Leu | ALI | A.523 | Val | ALI |
| B.372 | His | DAC | A.355 | Tyr | DAC |
| B.372 | His | PII | A.355 | Tyr | PII |
| B.414 | Leu | ALI | A.352 | Leu | ALI |
| B.421 | Phe | PII | A.387 | Trp | PII |
| B.425 | Asn | DON | A.385 | Tyr | DAC |
| B.599 | Trp | PII | A.205 | Phe | PII |
| B.603 | Ala | ALI | A.344 | Val | ALI |
| B.604 | Val | ALI | A.534 | Leu | ALI |
| B.607 | Leu | ALI | A.349 | Val | ALI |

| Site 1: 12-LOX | | | Site 2: COX2 | | |
| --- | --- | --- | --- | --- | --- |
| **Chain.ID** | **A. A.** | **Type** | **Chain.ID** | **A. A.** | **Type** |
| A.187 | Leu | ALI | A.113 | Met | ALI |
| A.190 | Val | ALI | A.116 | Val | ALI |
| A.194 | Leu | ALI | A.120 | Arg | ALI |
| A.357 | Ile | ALI | A.345 | Ile | ALI |
| A.361 | Leu | ALI | A.359 | Leu | ALI |
| A.365 | His | DAC | A.353 | Ser | ACC |
| A.365 | His | PII | A.355 | Tyr | PII |
| A.540 | His | DAC | A.192 | Gln | ACC |
| A.593 | Ile | ALI | A.527 | Ala | ALI |
| A.597 | Leu | ALI | A.523 | Val | ALI |

| Site 1: 15-LOX | | | Site 2: COX2 | | |
| --- | --- | --- | --- | --- | --- |
| **Chain.ID** | **A. A.** | **Type** | **Chain.ID** | **A. A.** | **Type** |
| A.366 | His | PII | A.355 | Tyr | PII |
| A.403 | Arg | ACC | A.522 | Met | ACC |
| A.404 | Ala | ALI | A.523 | Val | ALI |
| A.407 | Gly | PII | A.526 | Gly | PII |
| A.408 | Leu | DON | A.527 | Ala | DON |
| A.408 | Leu | ALI | A.527 | Ala | ALI |
| A.409 | Val | ALI | A.120 | Arg | ALI |
| A.419 | Met | ALI | A.531 | Leu | ALI |
| A.593 | Ile | ALI | A.534 | Leu | ALI |
| A.594 | Val | ALI | A.345 | Ile | ALI |
| A.597 | Leu | ALI | A.349 | Val | ALI |
| A.663 | Ile | ACC | A.353 | Ser | ACC |

| **S.NO** | **COX-1** | | | **COX-2** | | |
| --- | --- | --- | --- | --- | --- | --- |
|  | **Chain.ID** | **A. A.** | **Type** | **Chain.ID** | **A. A.** | **Type** |
|  | A.90 | His | DAC | A.90 | His | DAC |
|  | A.90 | His | PII | A.90 | His | PII |
|  | A.93 | Leu | ALI | A.93 | Leu | ALI |
|  | A.116 | Val | ALI | A.116 | Val | ALI |
|  | A.120 | Arg | DON | A.120 | Arg | DON |
|  | A.120 | Arg | DON | A.120 | Arg | DON |
|  | A.192 | Gln | ACC | A.192 | Gln | ACC |
|  | A.205 | Phe | PII | A.205 | Phe | PII |
|  | A.209 | Phe | PII | A.209 | Phe | PII |
|  | A.228 | Val | ALI | A.228 | Val | ALI |
|  | A.344 | Val | ALI | A.344 | Val | ALI |
|  | A.348 | Tyr | PII | A.348 | Tyr | PII |
|  | A.349 | Val | ALI | A.349 | Val | ALI |
|  | A.352 | Leu | ACC | A.352 | Leu | ACC |
|  | A.352 | Leu | ALI | A.352 | Leu | ALI |
|  | A.353 | Ser | ACC | A.353 | Ser | ACC |
|  | A.355 | Tyr | DAC | A.355 | Tyr | DAC |
|  | A.355 | Tyr | PII | A.355 | Tyr | PII |
|  | A.359 | Leu | ALI | A.359 | Leu | ALI |
|  | A.377 | Ile | ALI | A.377 | Ile | ALI |
|  | A.381 | Phe | PII | A.381 | Phe | PII |
|  | A.384 | Leu | ALI | A.384 | Leu | ALI |
|  | A.385 | Tyr | DAC | A.385 | Tyr | DAC |
|  | A.385 | Tyr | PII | A.385 | Tyr | PII |
|  | A.387 | Trp | PII | A.387 | Trp | PII |
|  | A.517 | Ile | ALI | A.517 | Ile | ALI |
|  | A.518 | Phe | PII | A.518 | Phe | PII |
|  | A.522 | Met | ACC | A.522 | Met | ACC |
|  | A.522 | Met | ALI | A.522 | Met | ALI |
|  | A.523 | Ile | ALI | A.523 | Val | ALI |
|  | A.524 | Glu | ACC | A.524 | Glu | ACC |
|  | A.526 | Gly | PII | A.526 | Gly | PII |
|  | A.527 | Ala | DON | A.527 | Ala | DON |
|  | A.527 | Ala | ALI | A.527 | Ala | ALI |
|  | A.530 | Ser | DAC | A.530 | Ser | DAC |
|  | A.534 | Leu | ALI | A.534 | Leu | ALI |

**MULTIPLE ALIGNMENTS OF 3 BINDING SITES:**

The alignment between 3 binding sites resulted in 35 combinations. Comparison between three predicted binding sites of Arachidonic acid at a time revealed a pattern of between 13 and 3 common physiochemical properties. The 3 binding sites that were recognised to be most similar to each other are those of 5LOX. COX1 and COX2.

| **S.No** | **COMPARED PROTEINS** | **NO. OF DETECTED FEATURES** | **SCORE** |
| --- | --- | --- | --- |
| **1)** | COX1-COX2-sLOX3 | 10 | 40.4966 |
| **2)** | 15LOX-COX1-sLOX3 | 7 | 28.5614 |
| **3)** | 15LOX-COX2-sLOX3 | 10 | 29.6998 |
| **4)** | 15LOX-sLOX1-sLOX3 | 7 | 26.7229 |
| **5)** | 15LOX-sLOX1-12LOX | 5 | 23.7942 |
| **6)** | 15LOX-sLOX1-5LOX | 8 | 25.967 |
| **7)** | 15LOX-sLOX1-COX1 | 6 | 21.9524 |
| **8)** | 15LOX-sLOX1-COX2 | 6 | 22.0494 |
| **9)** | 15LOX-12LOX-sLOX3 | 8 | 28.924 |
| **10)** | 15LOX-12LOX-5LOX | 8 | 31.5129 |
| **11)** | 15LOX-12LOX-COX1 | 7 | 29.7734 |
| **12)** | 15LOX-12LOX-COX2 | 8 | 32.2653 |
| **13)** | 15LOX-5LOX-sLOX3 | 11 | 34.9138 |
| **14)** | 15LOX-5LOX-COX1 | 9 | 31.6645 |
| **15)** | 15LOX-5LOX-COX2 | 7 | 32.1877 |
| **16)** | 15LOX-COX1-COX2 | 10 | 47.1464 |
| **17)** | sLOX1-COX1-sLOX3 | 9 | 26.5486 |
| **18)** | sLOX1-COX2-sLOX3 | 7 | 22.6782 |
| **19)** | sLOX1-12LOX-sLOX3 | 4 | 18.2054 |
| **20)** | sLOX1-12LOX-5LOX | 5 | 18.6627 |
| **21)** | sLOX1-12LOX-COX1 | 3 | 19.4546 |
| **22)** | sLOX1-12LOX-COX2 | 4 | 17.623 |
| **23)** | sLOX1-5LOX-sLOX3 | 9 | 28.2963 |
| **24)** | sLOX1-5LOX-COX1 | 7 | 22.318 |
| **25)** | sLOX1-5LOX-COX2 | 6 | 21.2019 |
| **26)** | sLOX1-COX1-COX2 | 6 | 32.5165 |
| **27)** | 12LOX-COX1-sLOX3 | 7 | 27.4447 |
| **28)** | 12LOX-COX2-sLOX3 | 8 | 27.1933 |
| **29)** | 12LOX-5LOX-sLOX3 | 9 | 30.6208 |
| **30)** | 12LOX-5LOX-COX1 | 6 | 27.756 |
| **31)** | 12LOX-5LOX-COX2 | 7 | 29.4465 |
| **32)** | 12LOX-COX1-COX2 | 10 | 40.7187 |
| **33)** | 5LOX-COX1-sLOX3 | 10 | 28.882 |
| **34)** | 5LOX-COX2-sLOX3 | 8 | 28.3092 |
| **35)** | 5LOX-COX1-COX2 | 13 | 45.4904 |

**1) COX1-COX2-sLOX3**


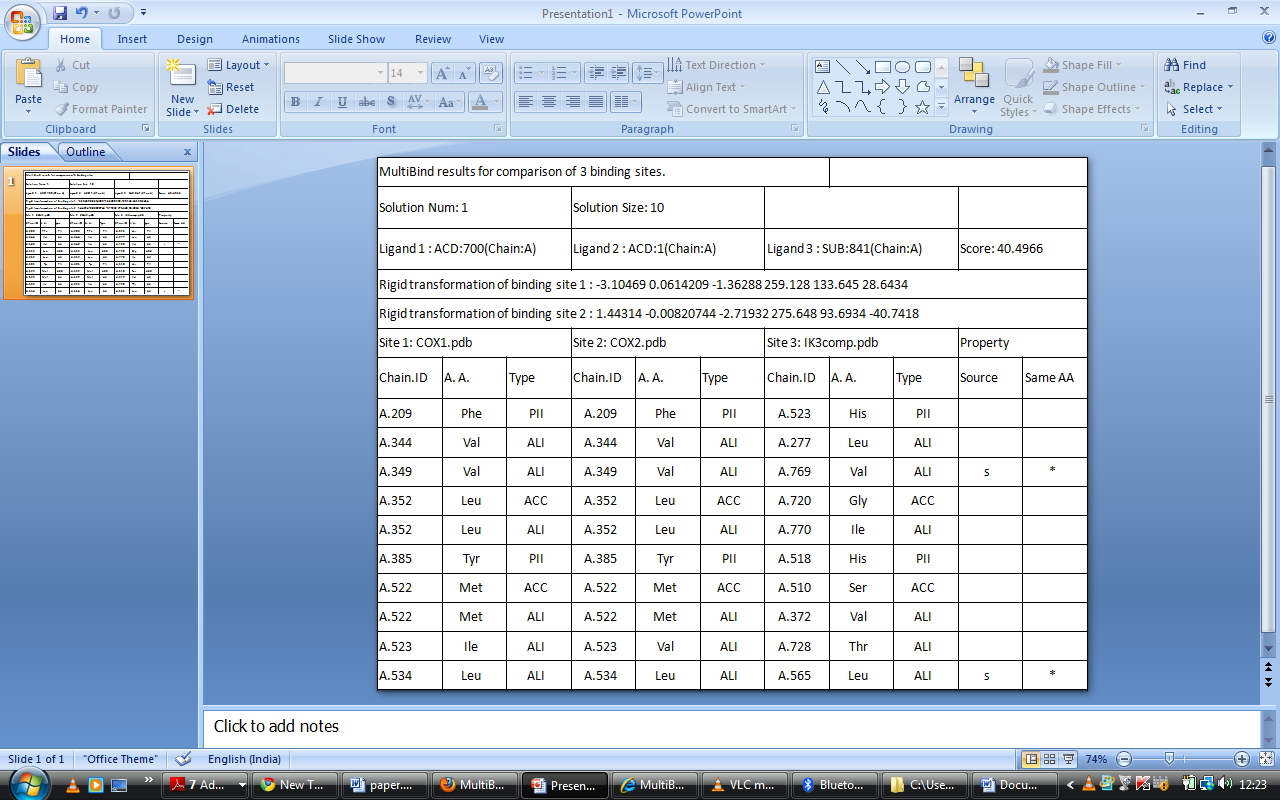


**2)15LOX-COX1-sLOX3**


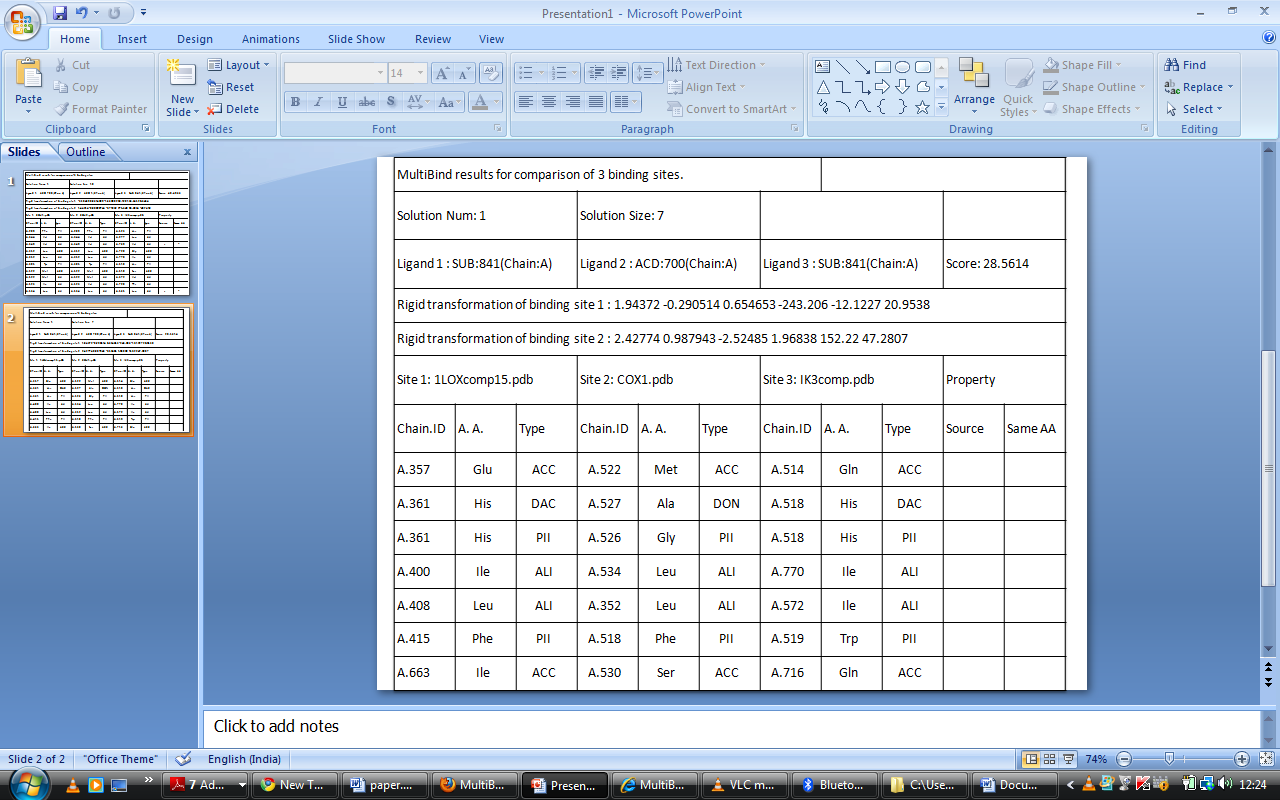


**3)15LOX-COX2-sLOX3**


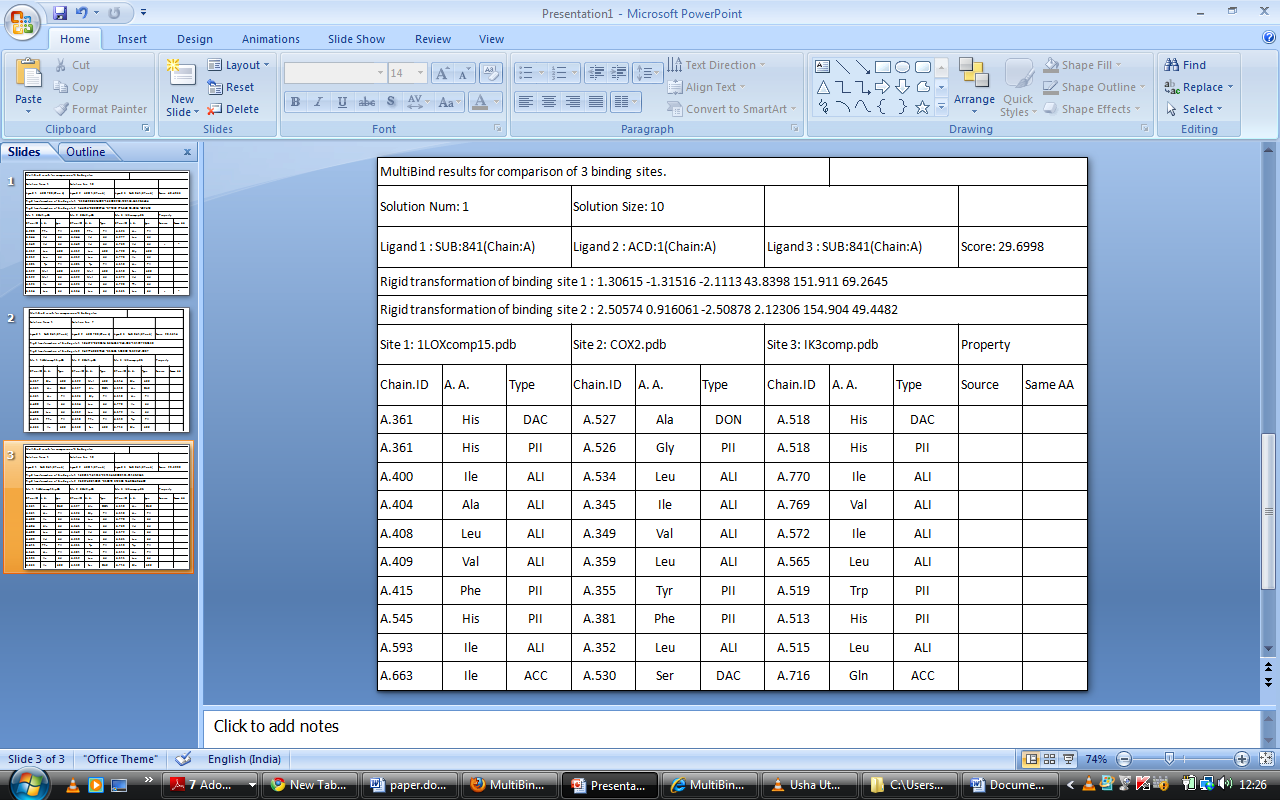


**4)15LOX-sLOX1-sLOX3**


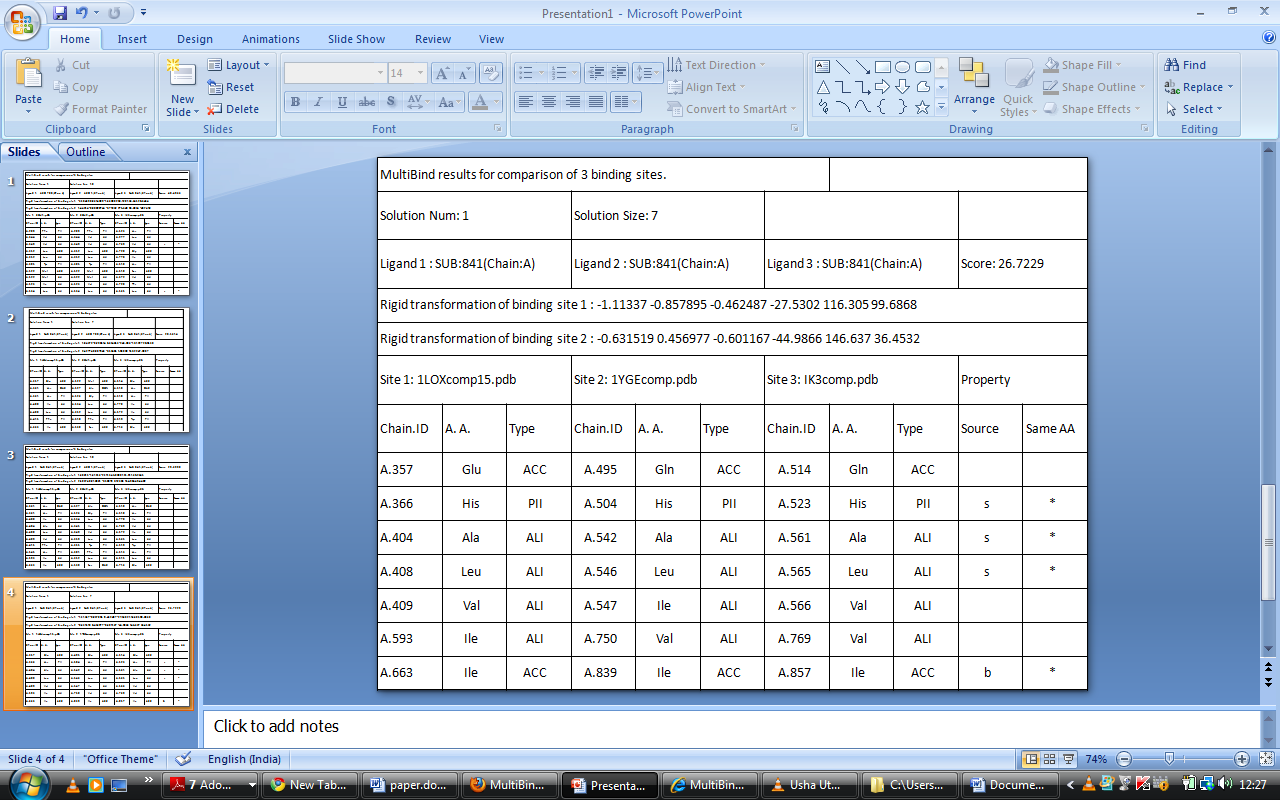


**5)15LOX-sLOX1-12LOX**


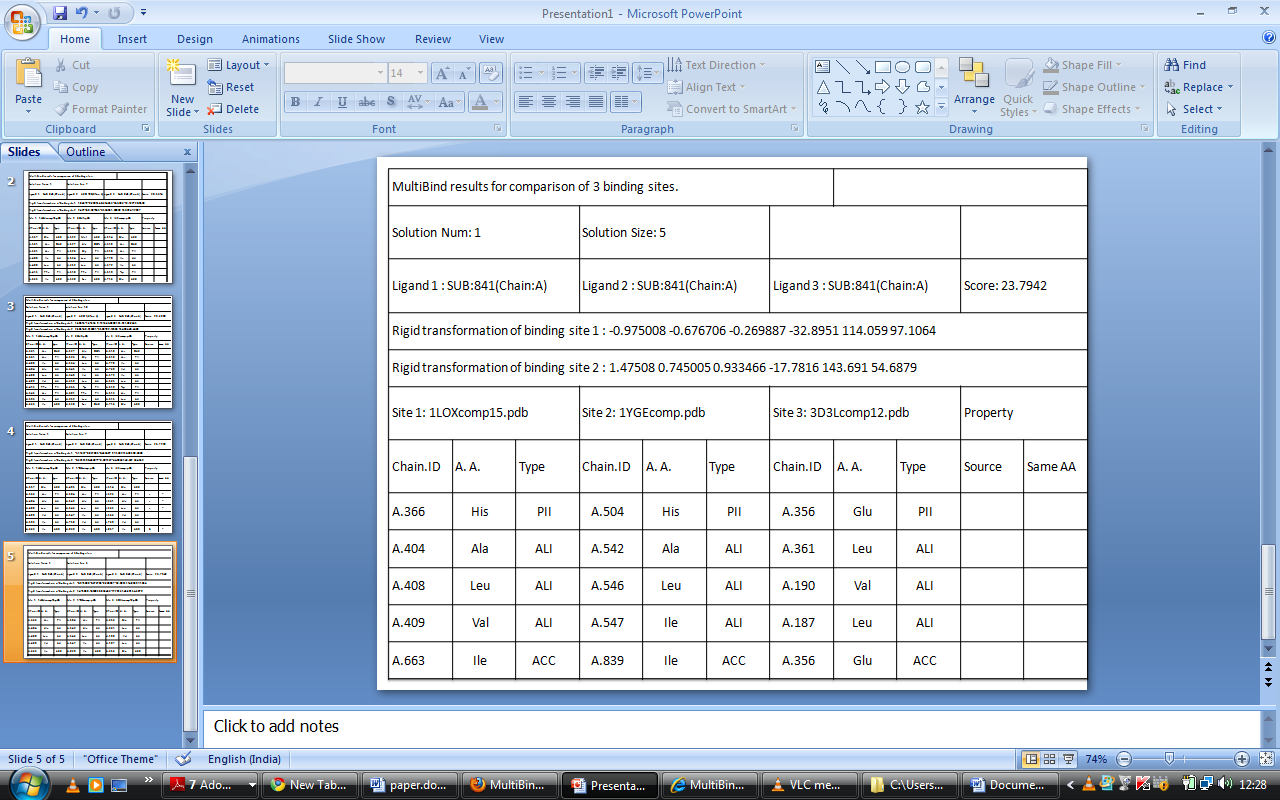


**6)15LOX-sLOX1-5LOX**


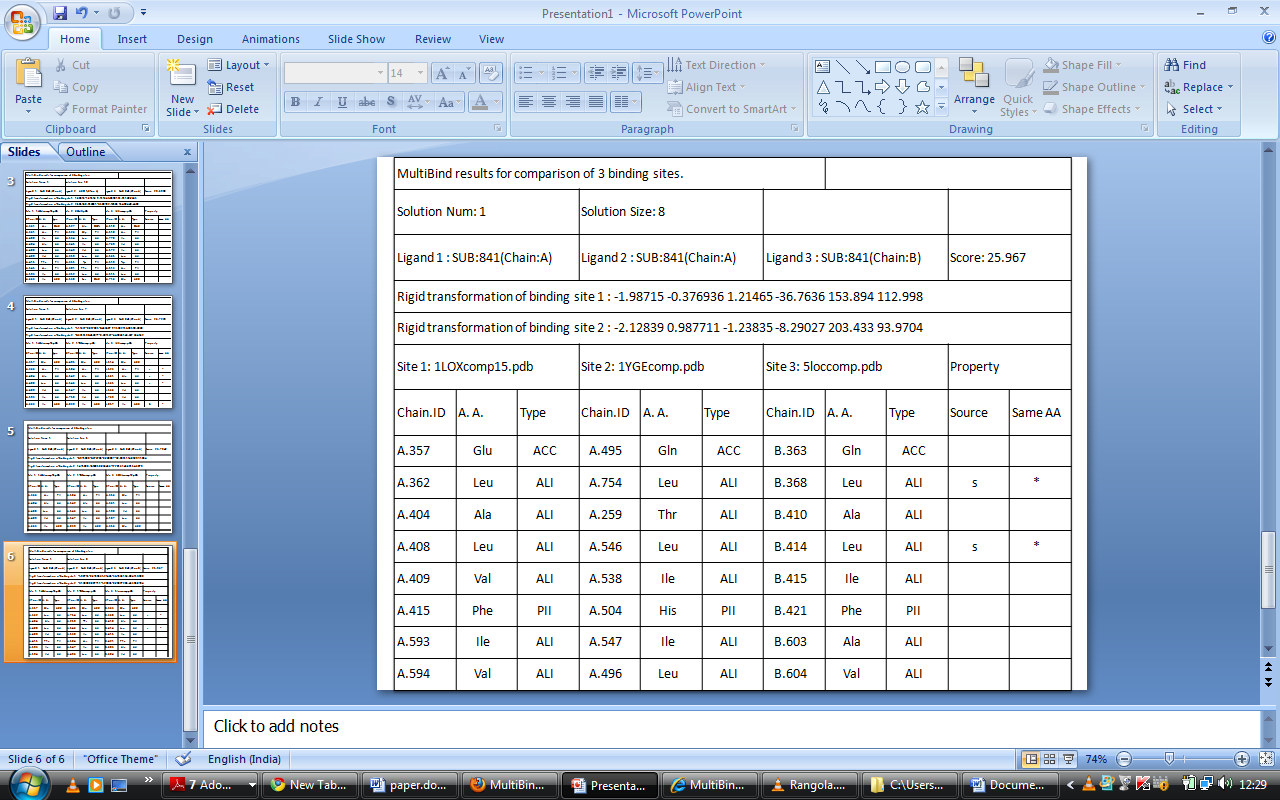


**7)15LOX-sLOX1-COX1**


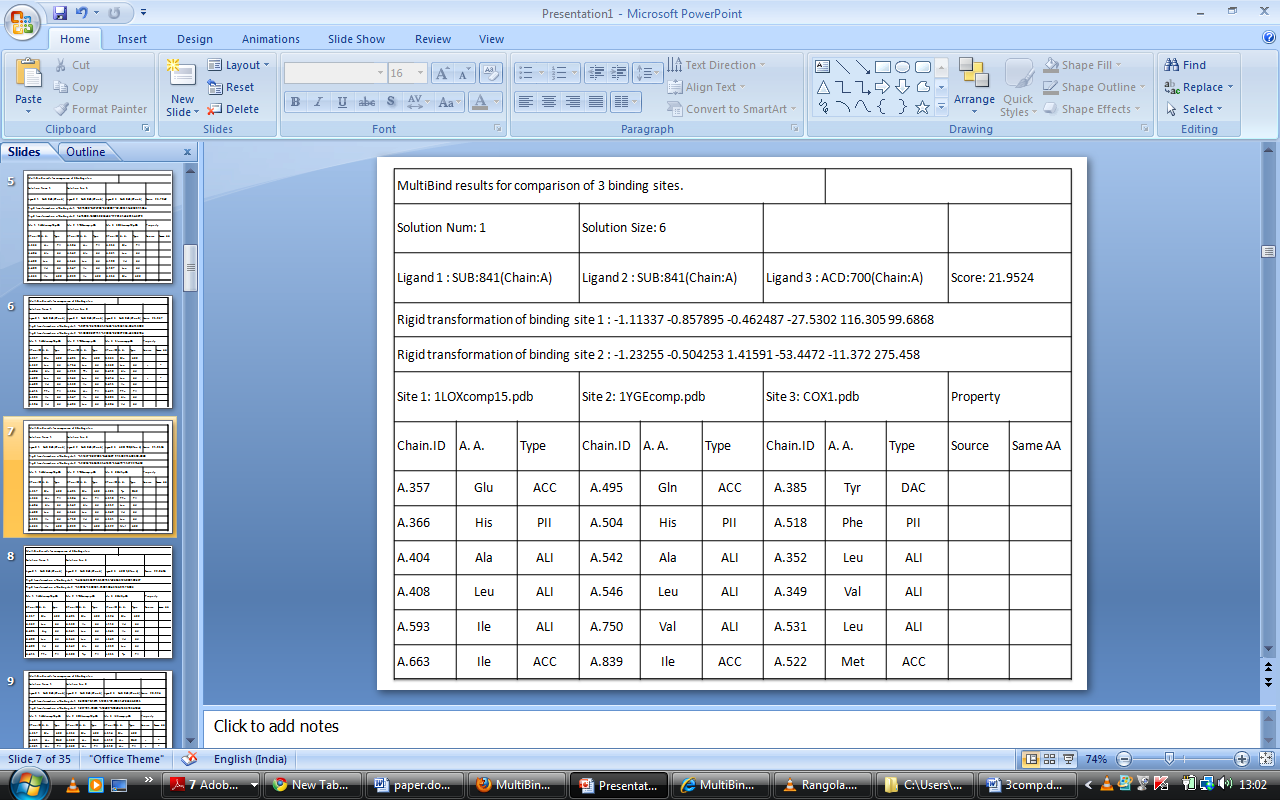


**8)15LOX-sLOX1-COX2**


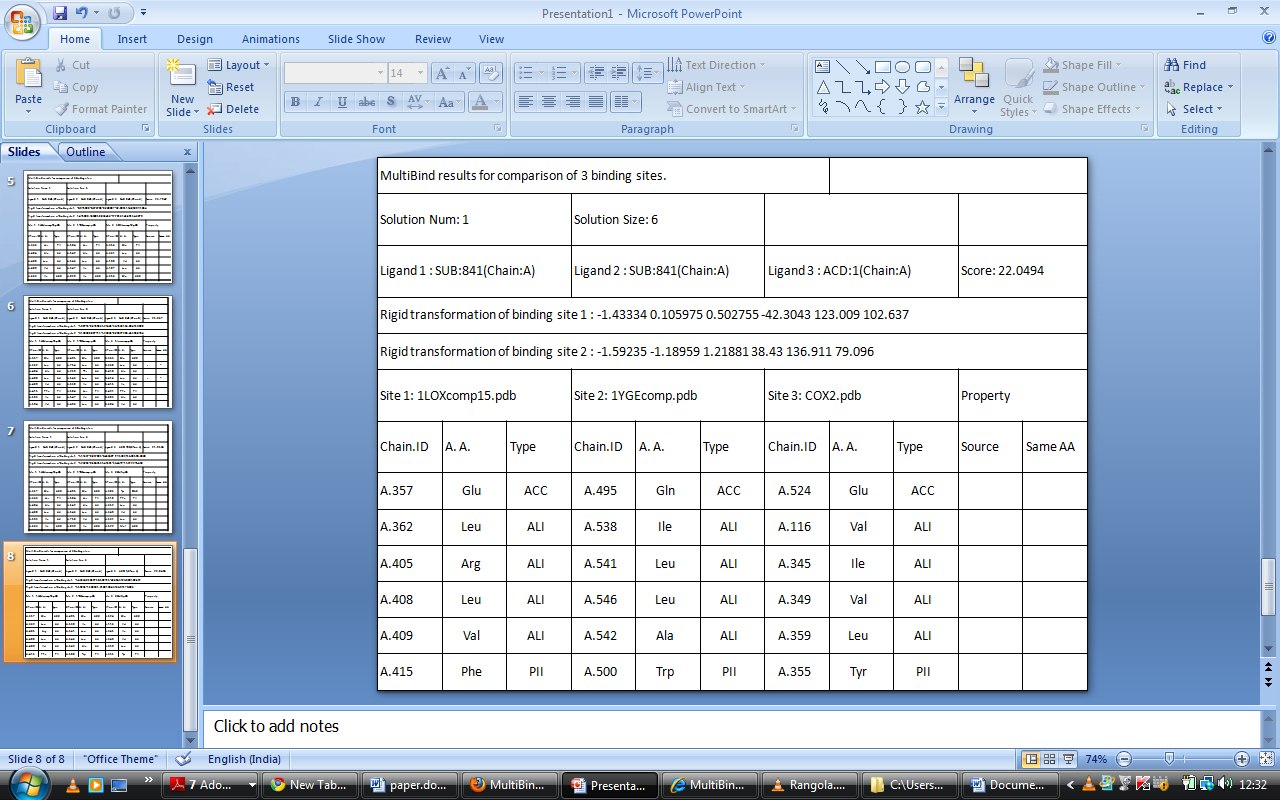


**9) 15LOX-12LOX-sLOX3**


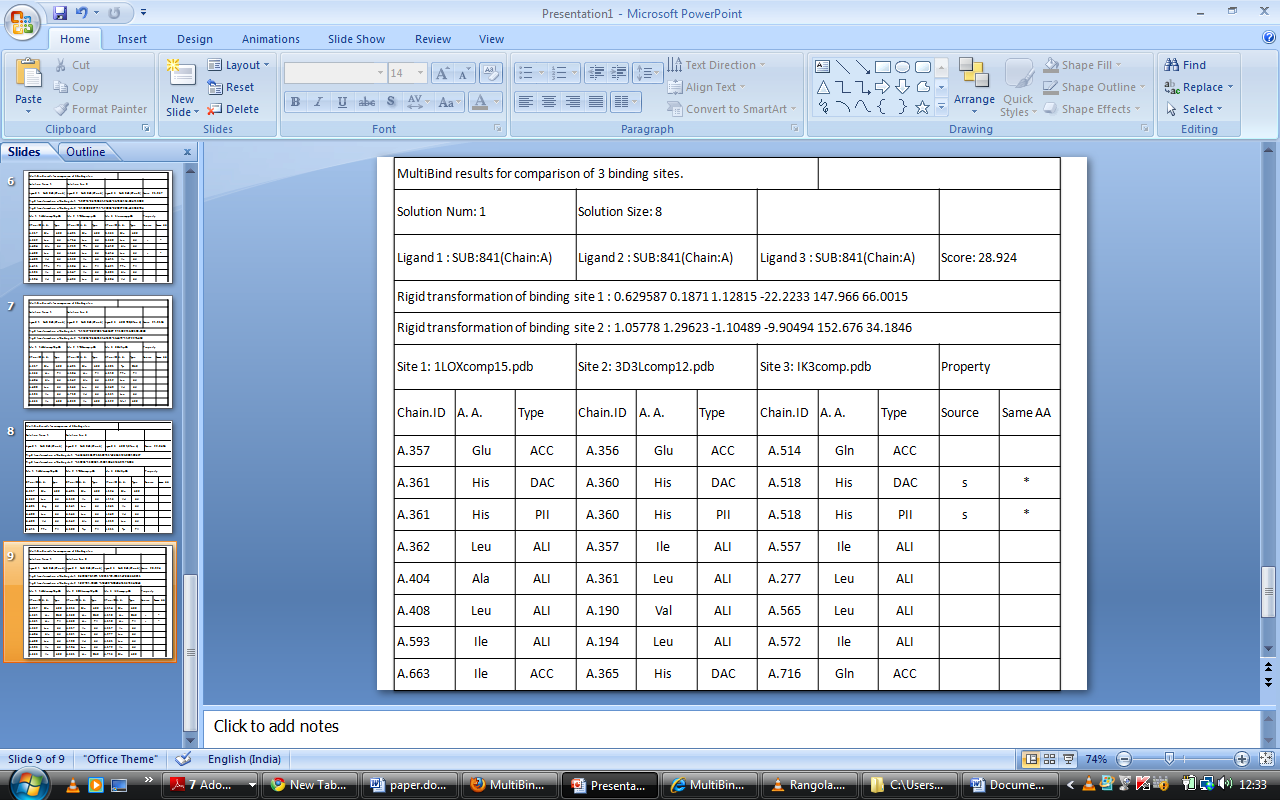


**10)15LOX-12LOX-5LOX**


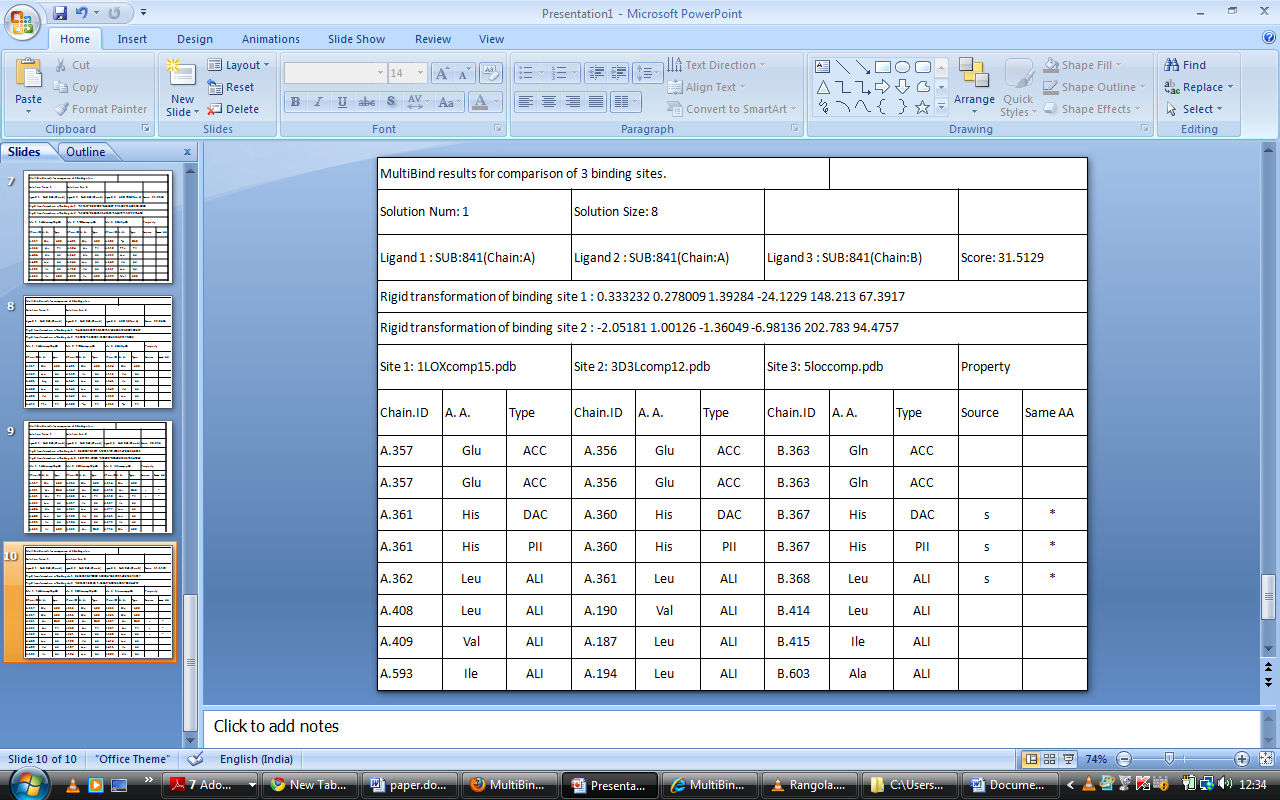


**11)15LOX-12LOX-COX1**


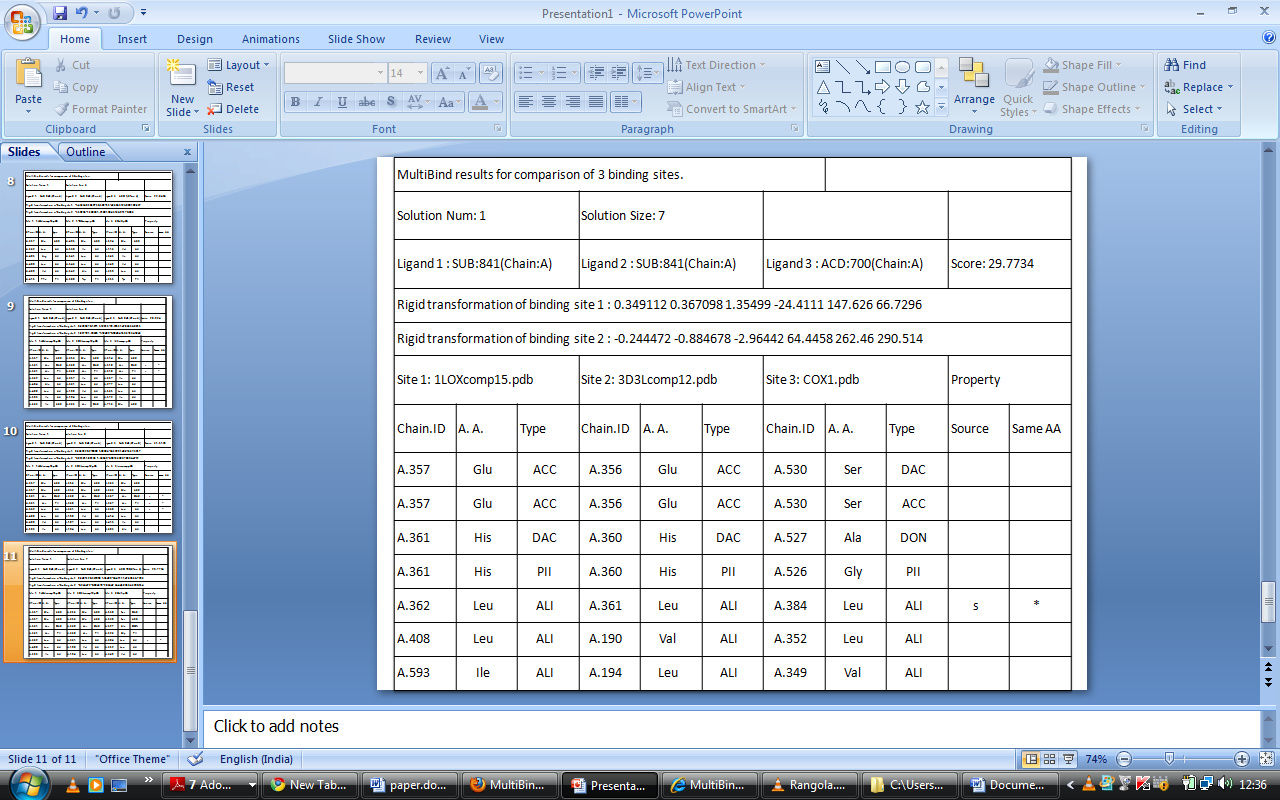


**12)15LOX-12LOX-COX2**


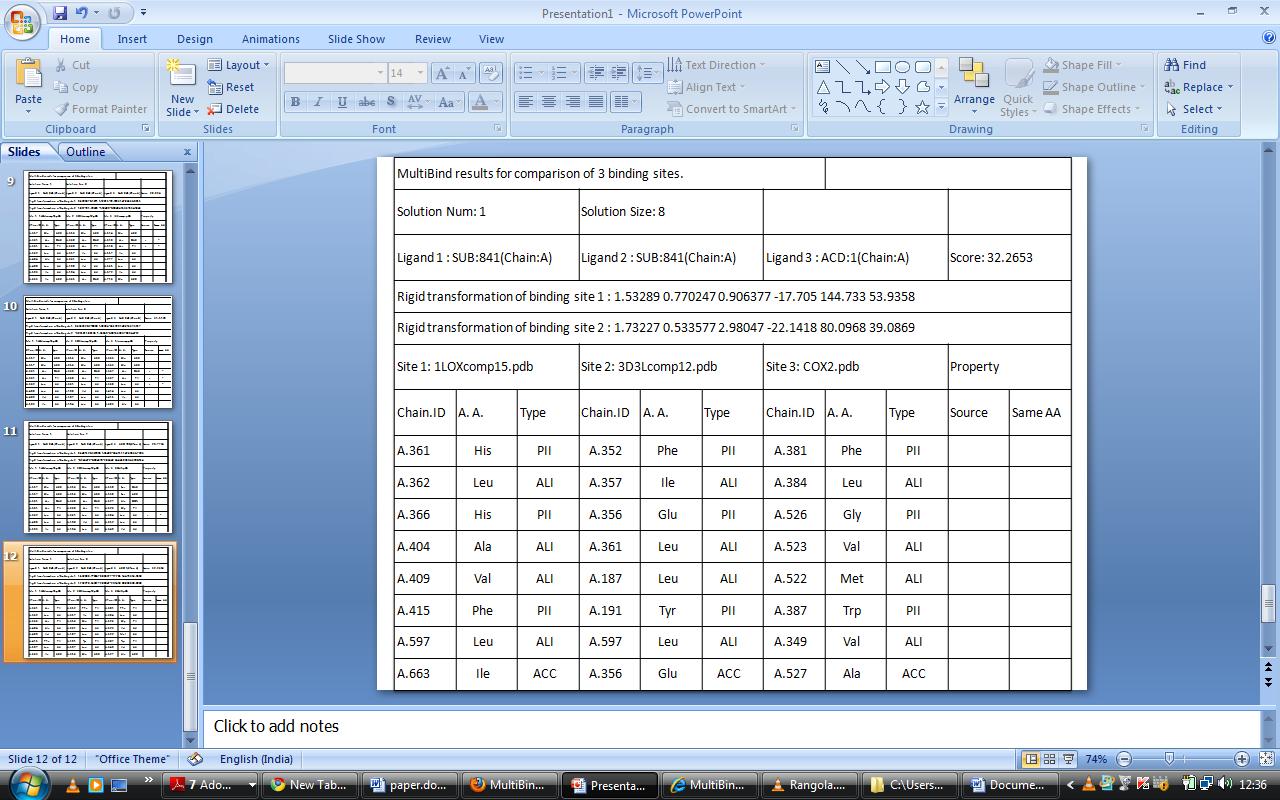


**13)15LOX-5LOX-sLOX3**


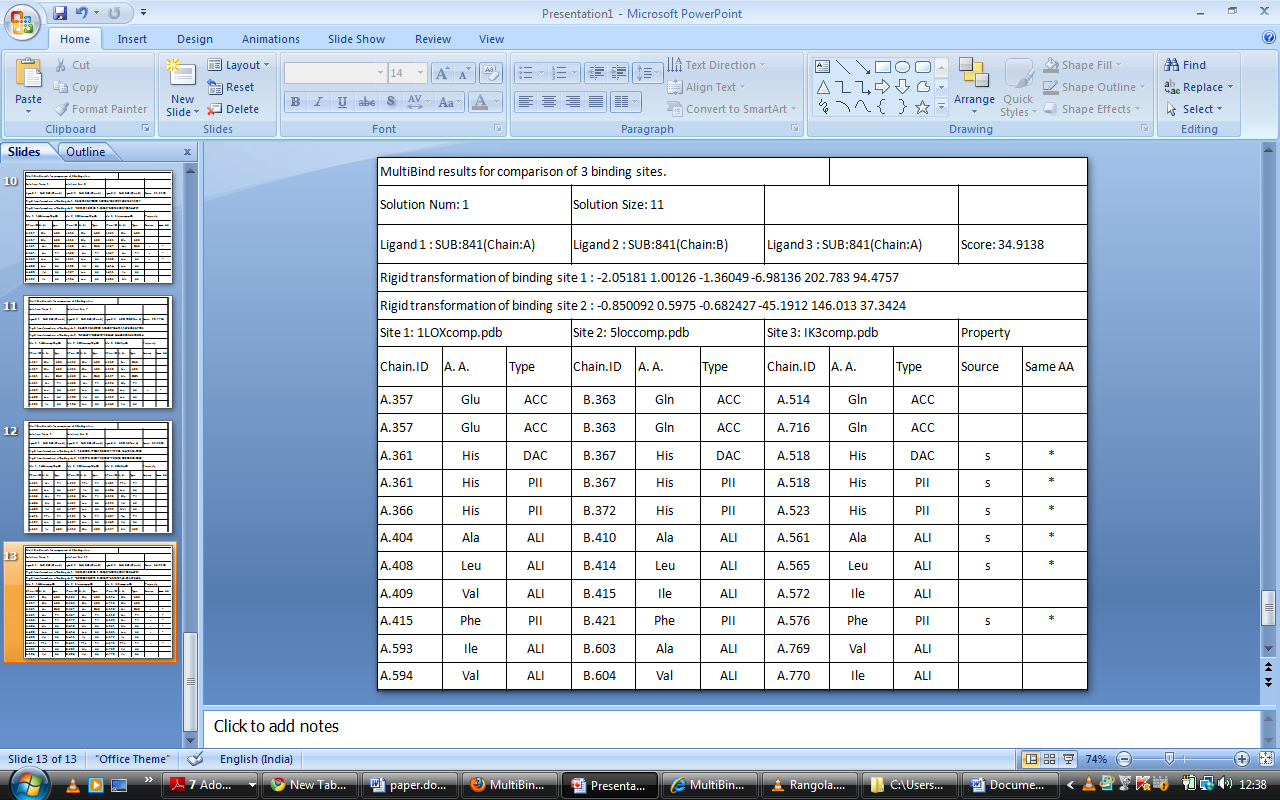


**14)15LOX-5LOX-COX1**


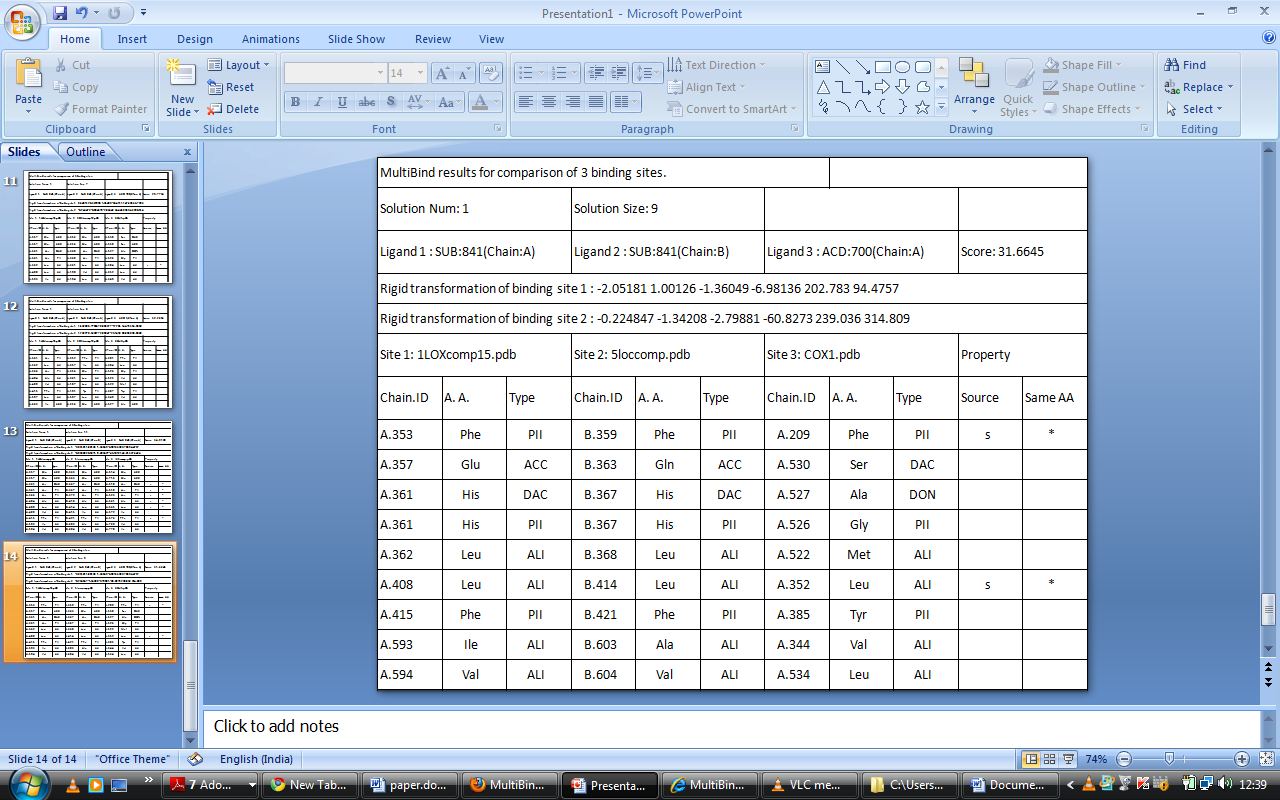


**15)15LOX-5LOX-COX2**


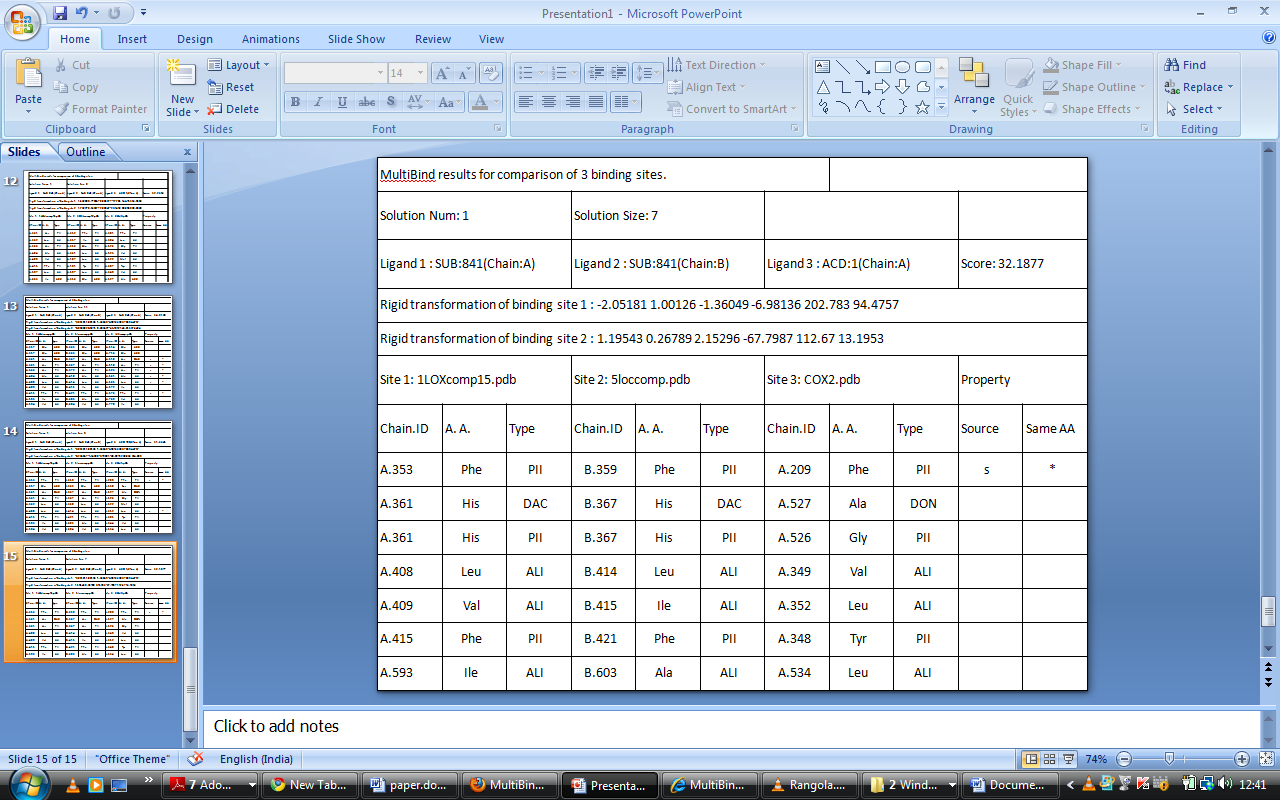


**16)15LOX-COX1-COX2**


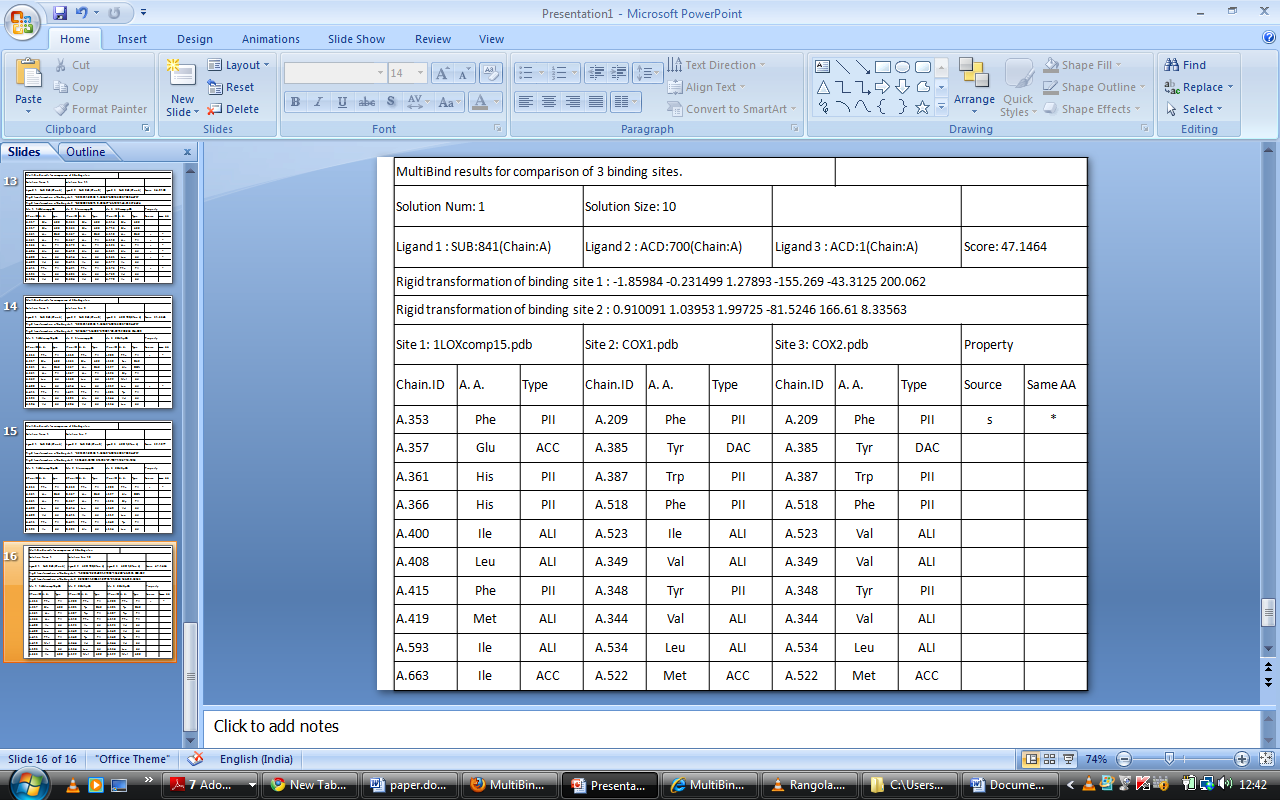


**17)sLOX1-COX1-sLOX3**


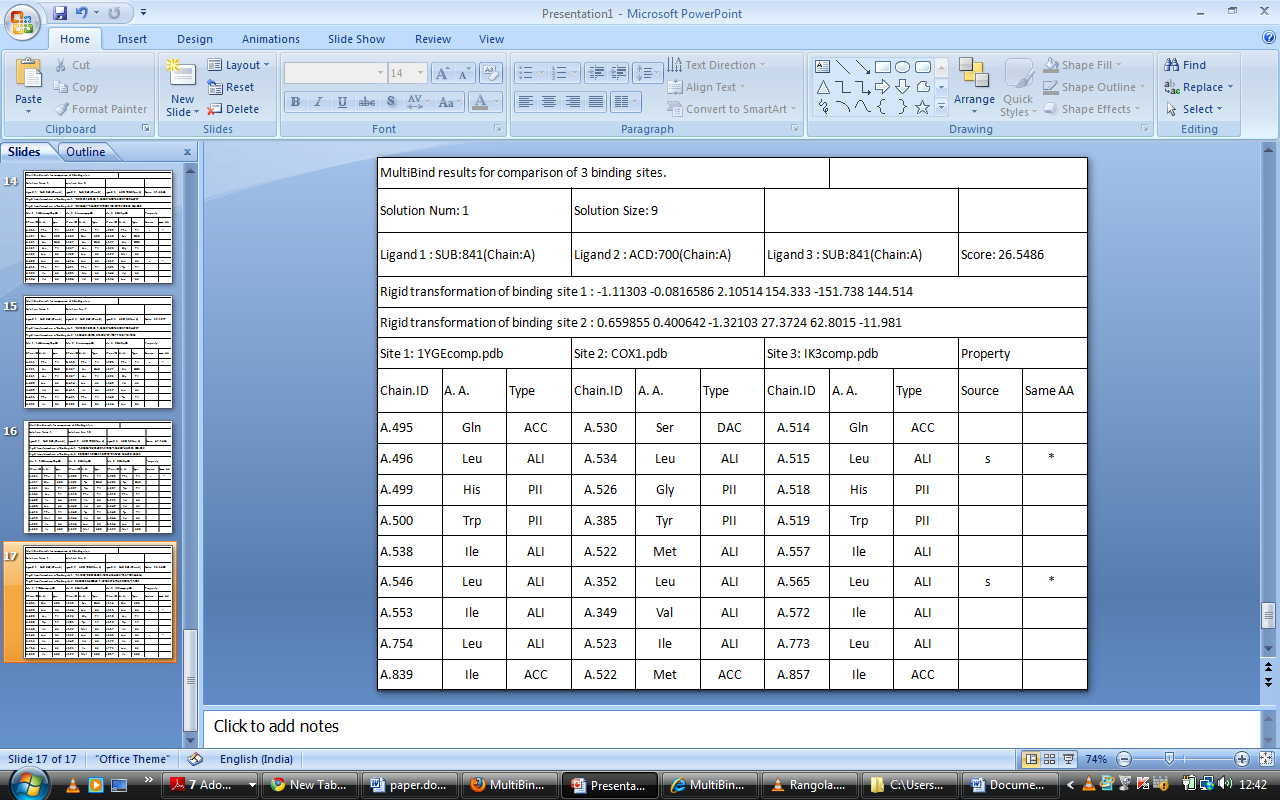


**18)sLOX1-COX2-sLOX3**


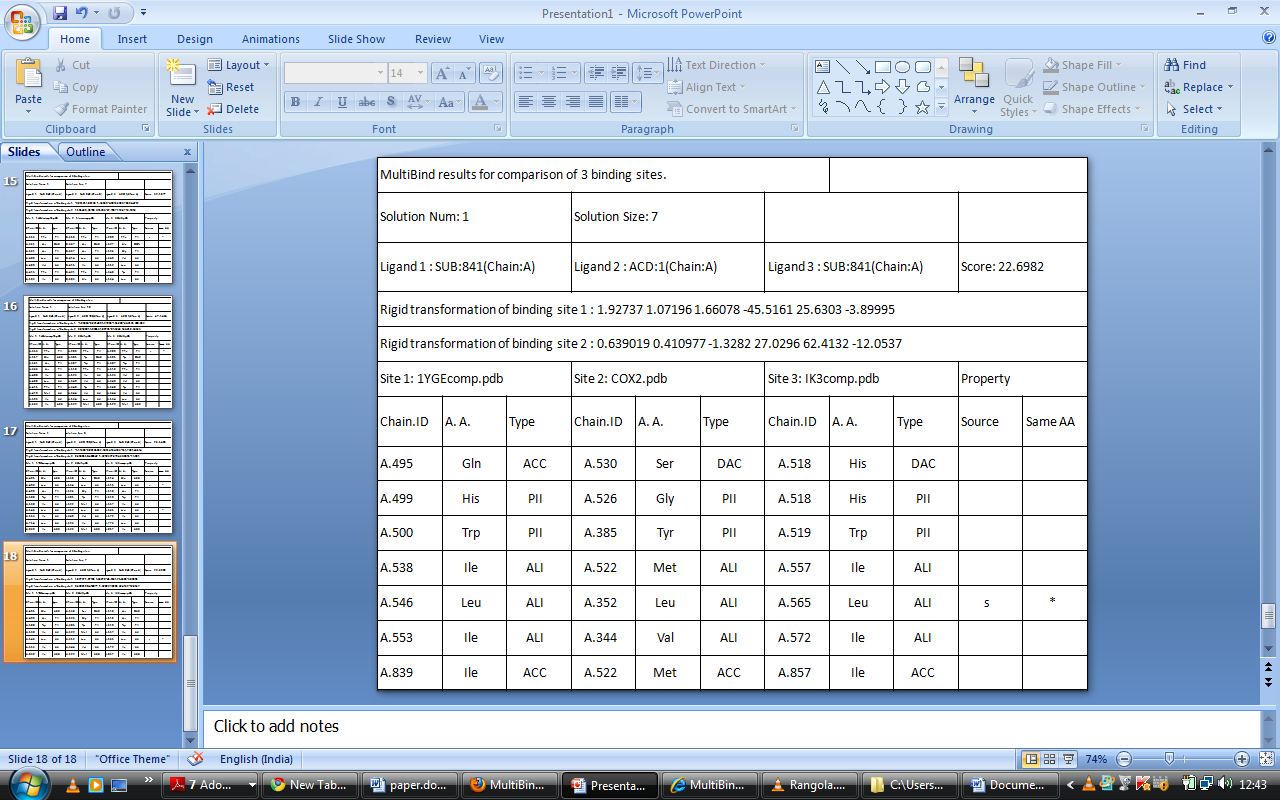


**19)sLOX1-12LOX-sLOX3**


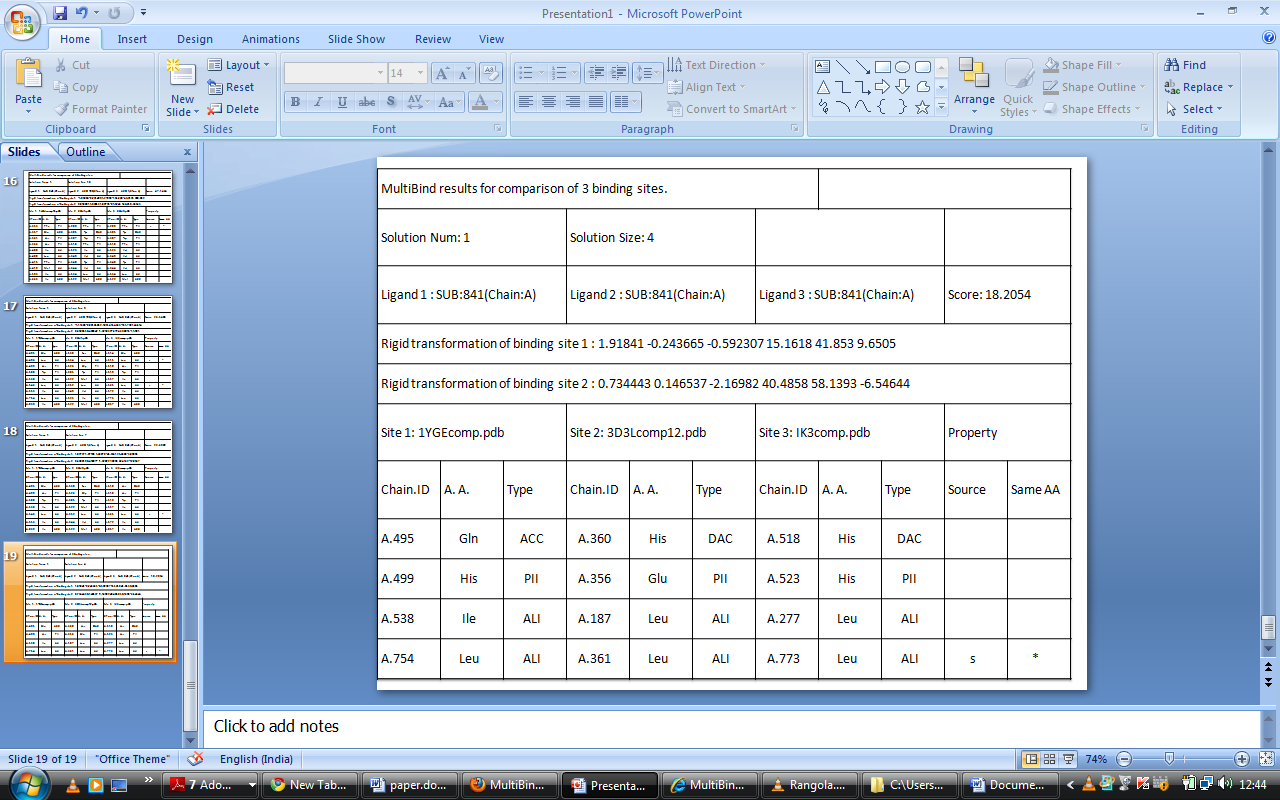


**20)sLOX1-12LOX-5LOX**


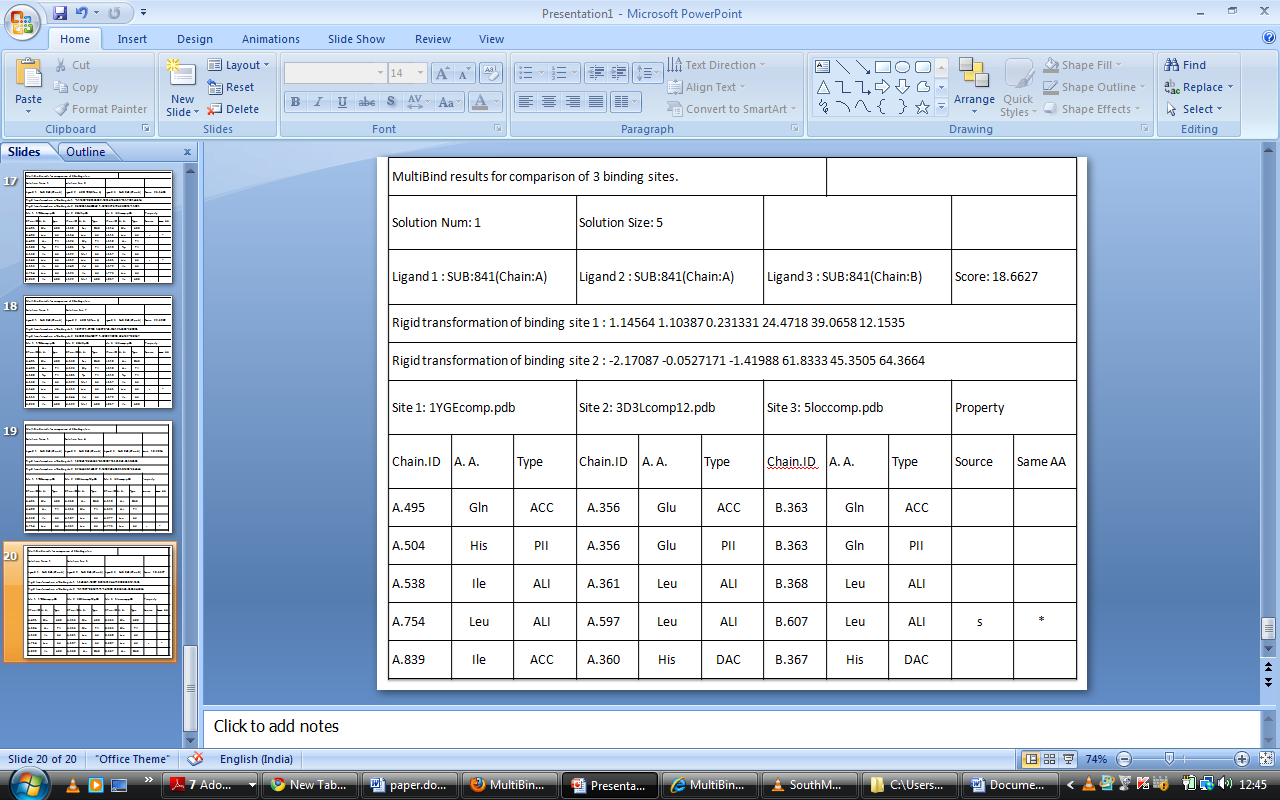


**21)sLOX1-12LOX-COX1**


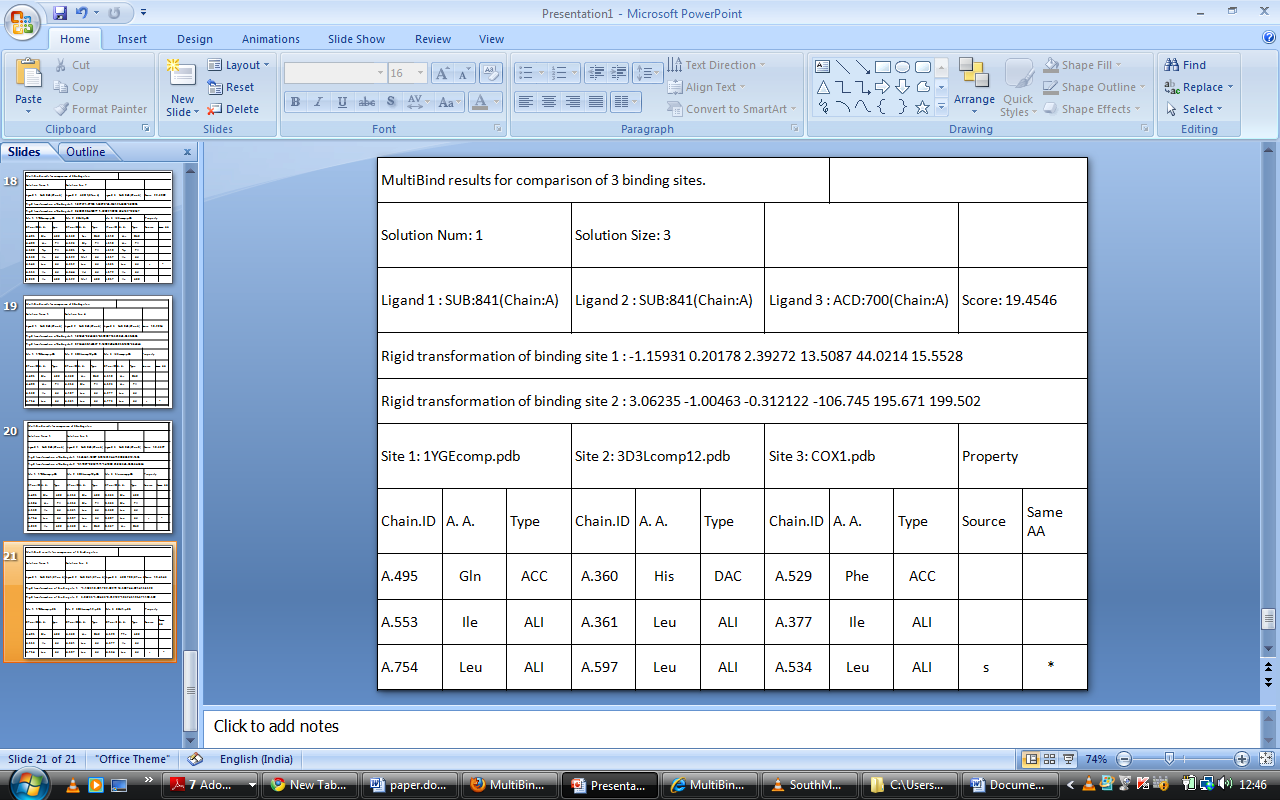


**22)sLOX1-12LOX-COX2**


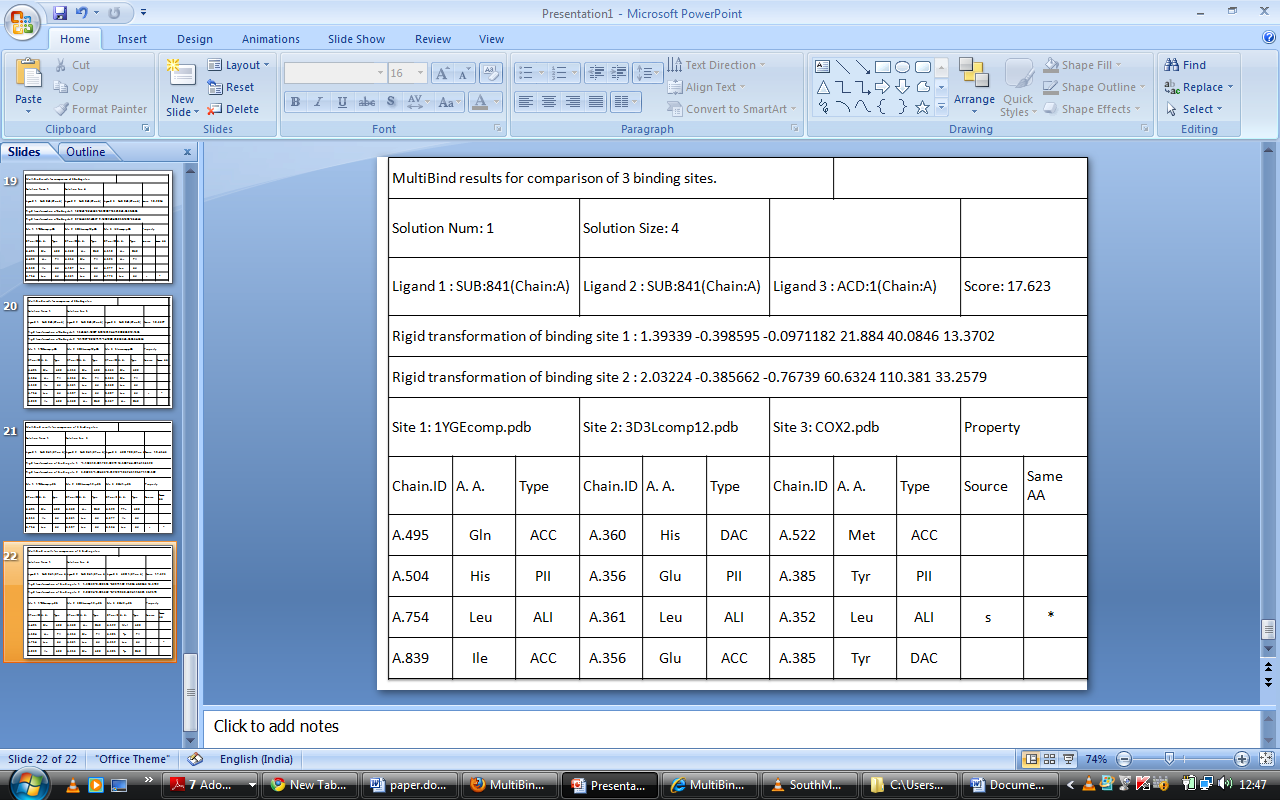


**23)sLOX1-5LOX-sLOX3**


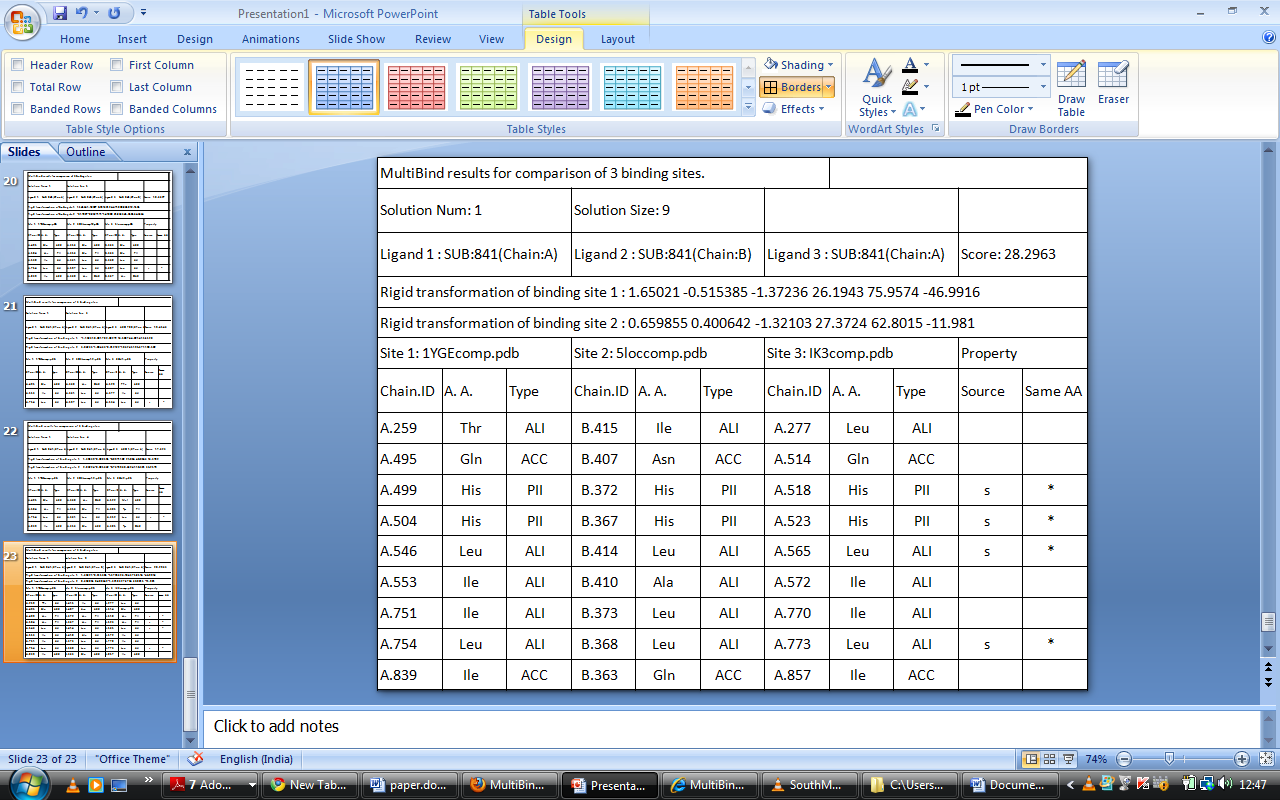


**24) sLOX1-5LOX-COX1**


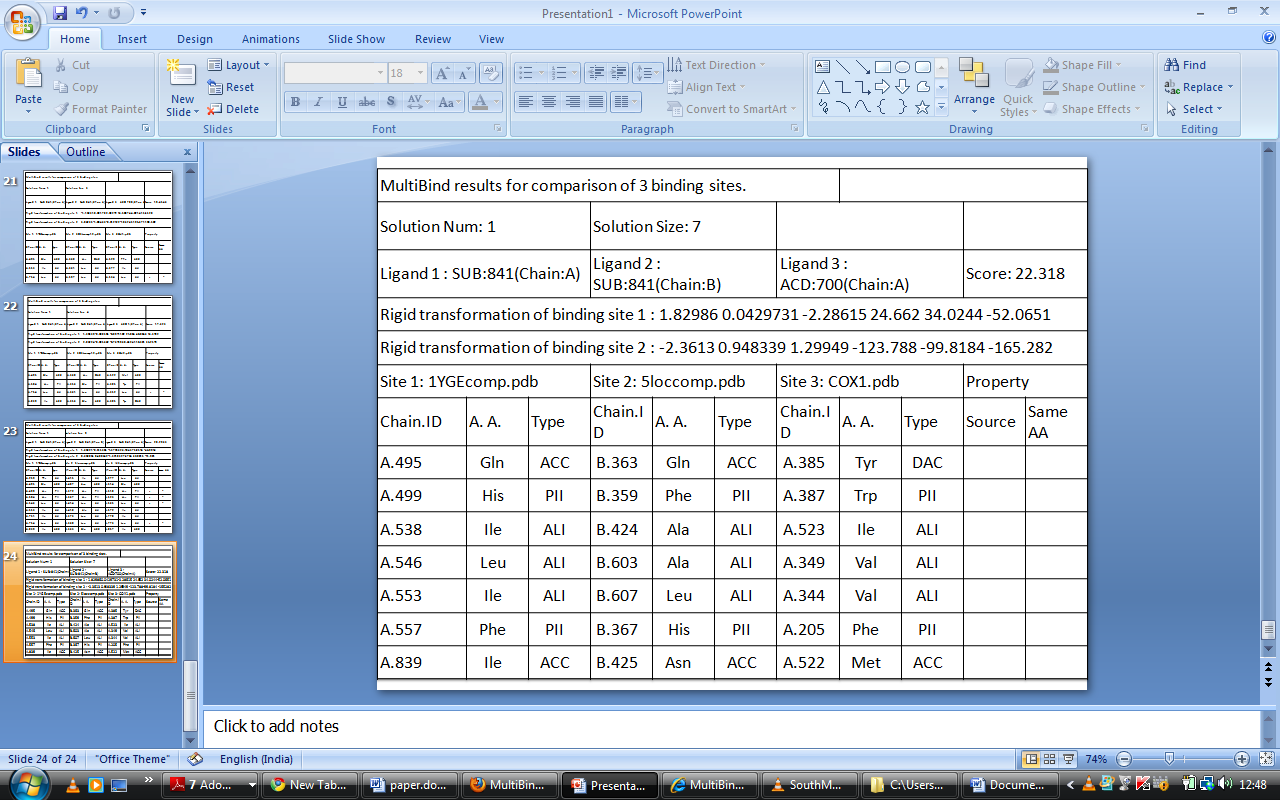


**25)sLOX1-5LOX-COX2**


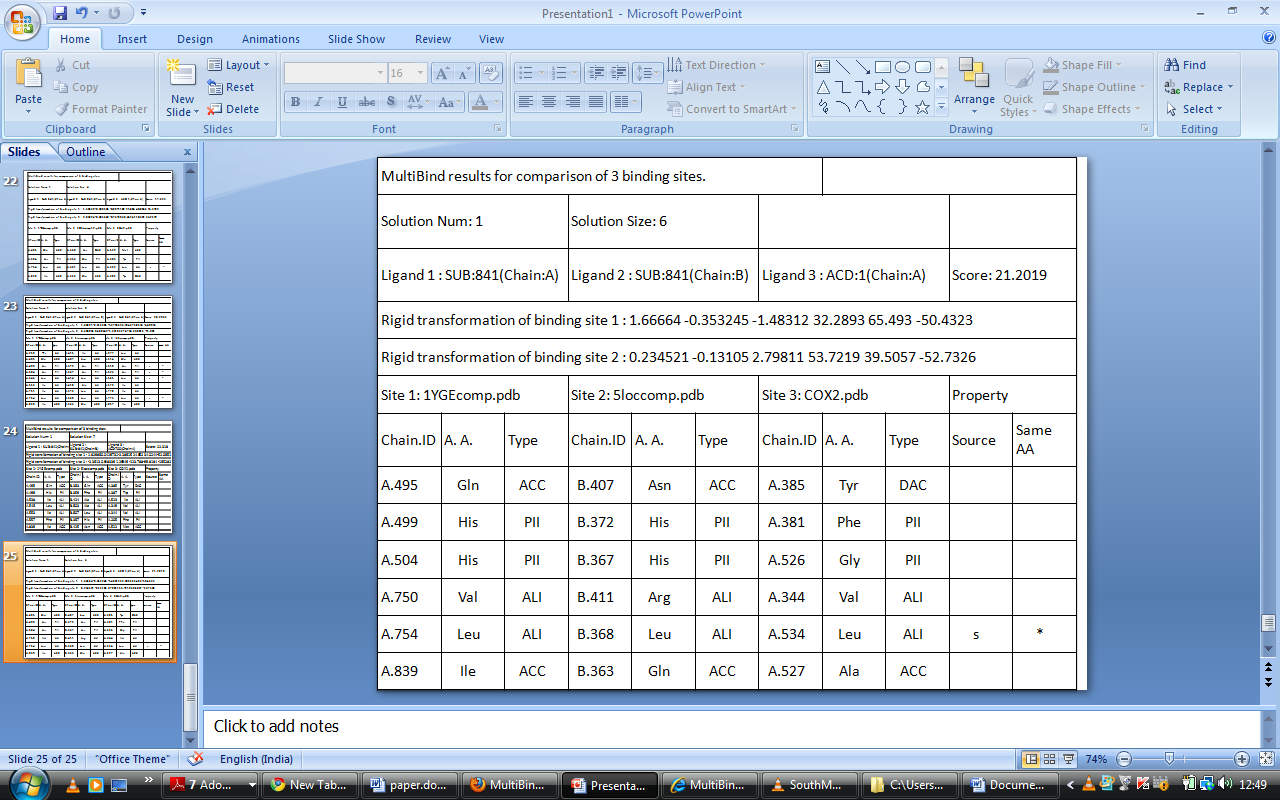


**26)sLOX1-COX1-COX2**


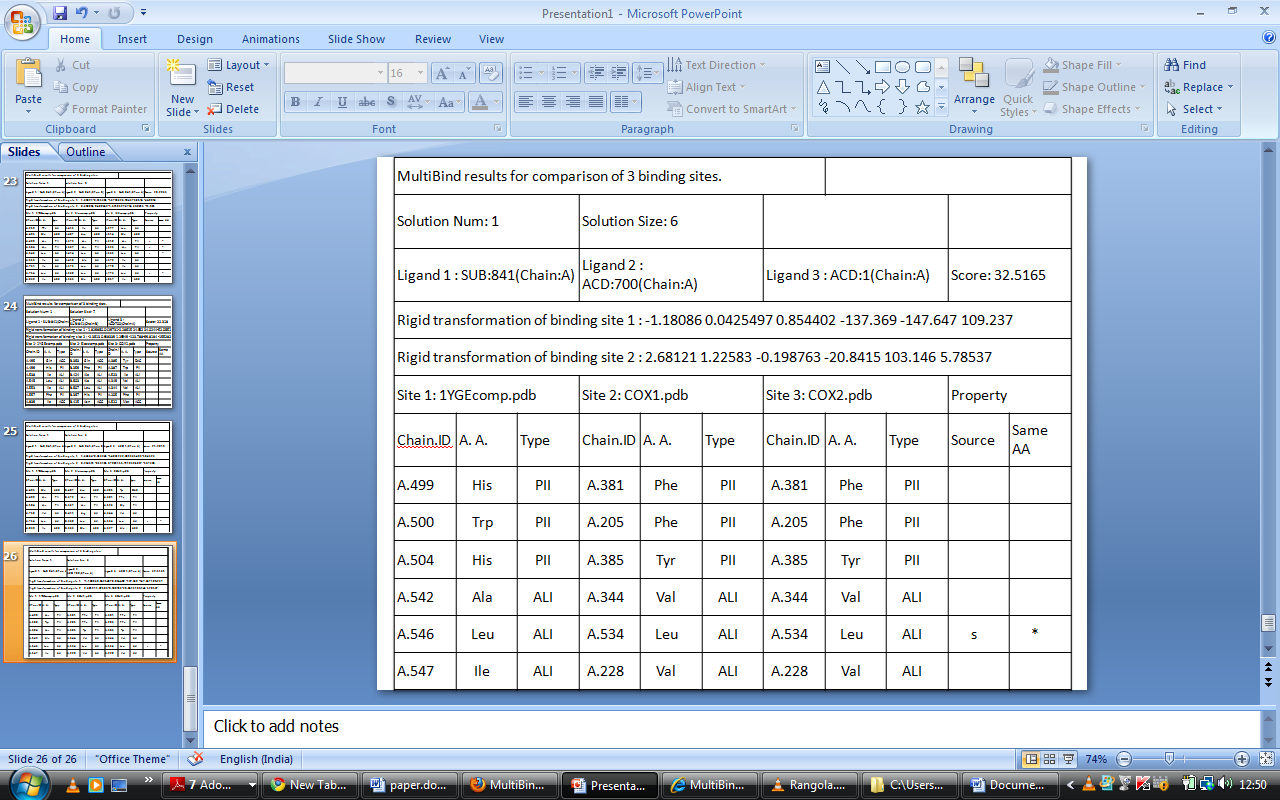


**27)12LOX-COX1-sLOX3**


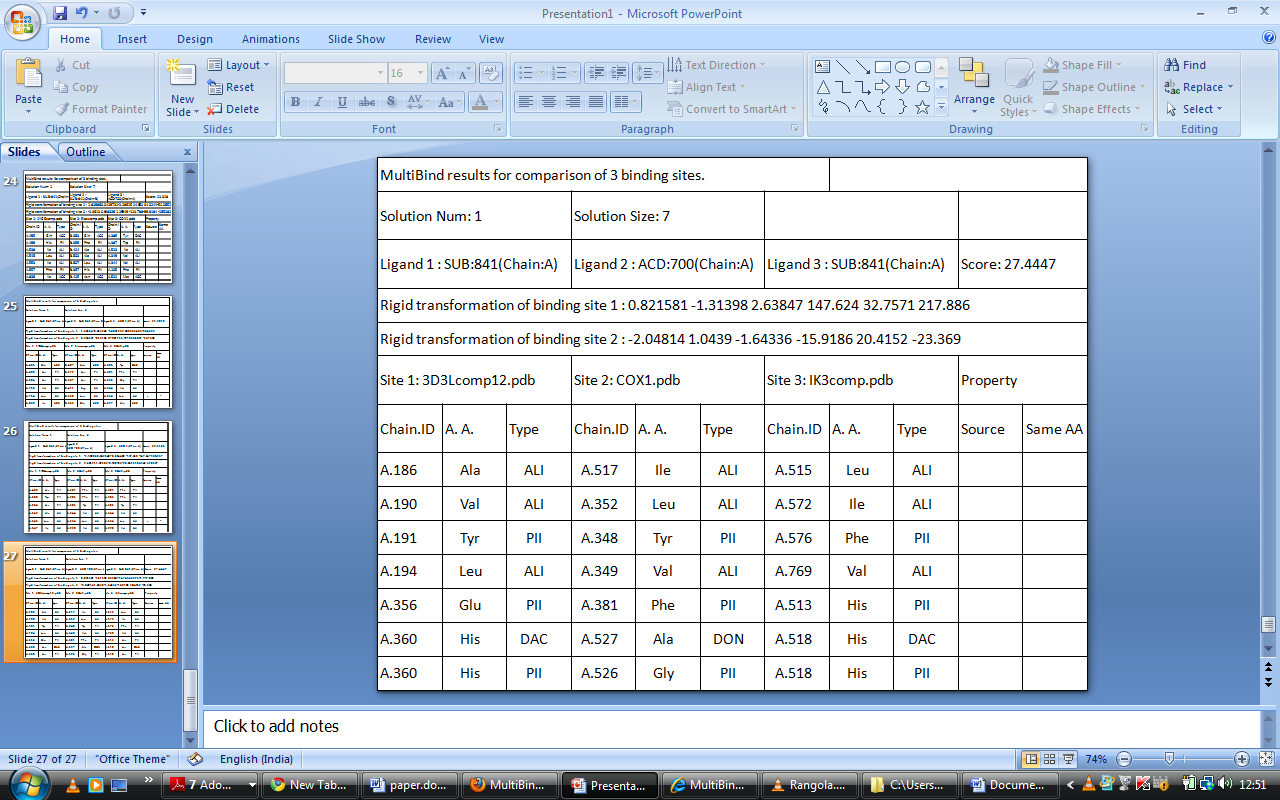


**28)12LOX-COX2-sLOX3**


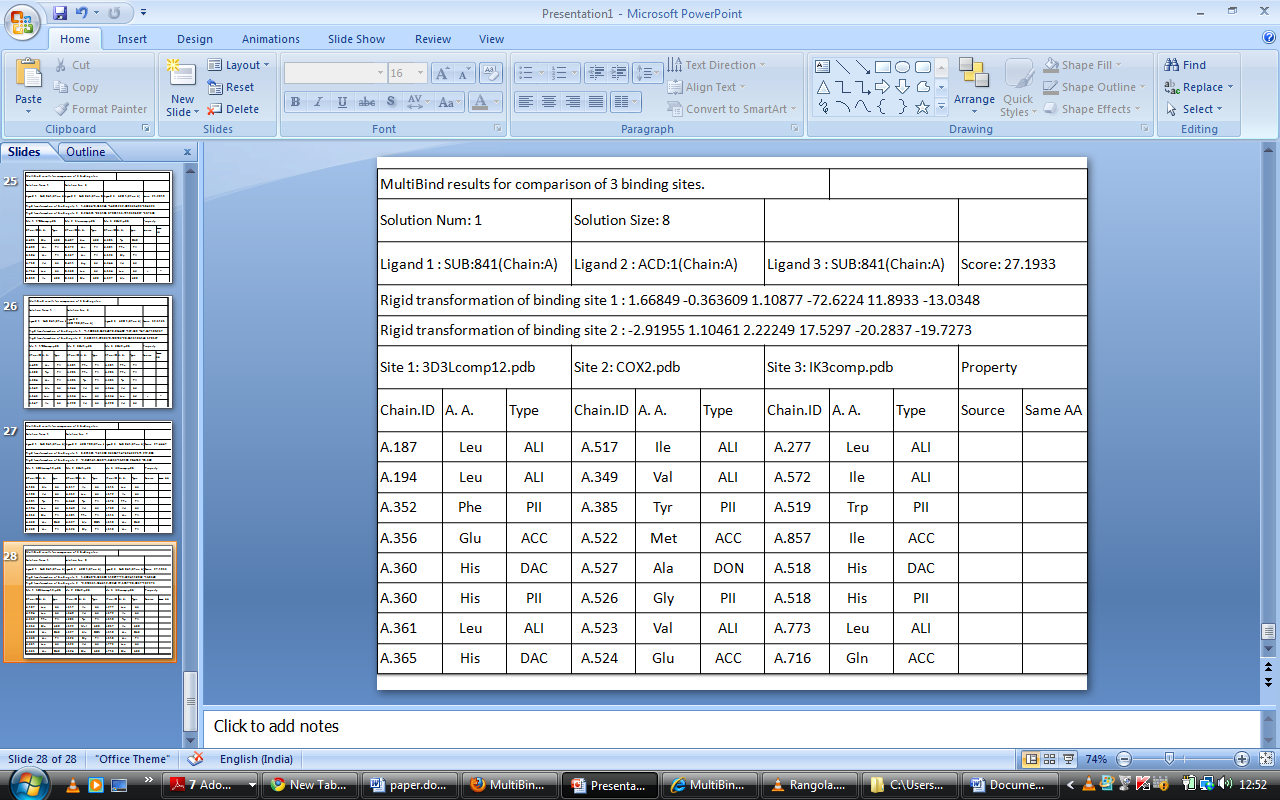


**29)12LOX-5LOX-sLOX3**


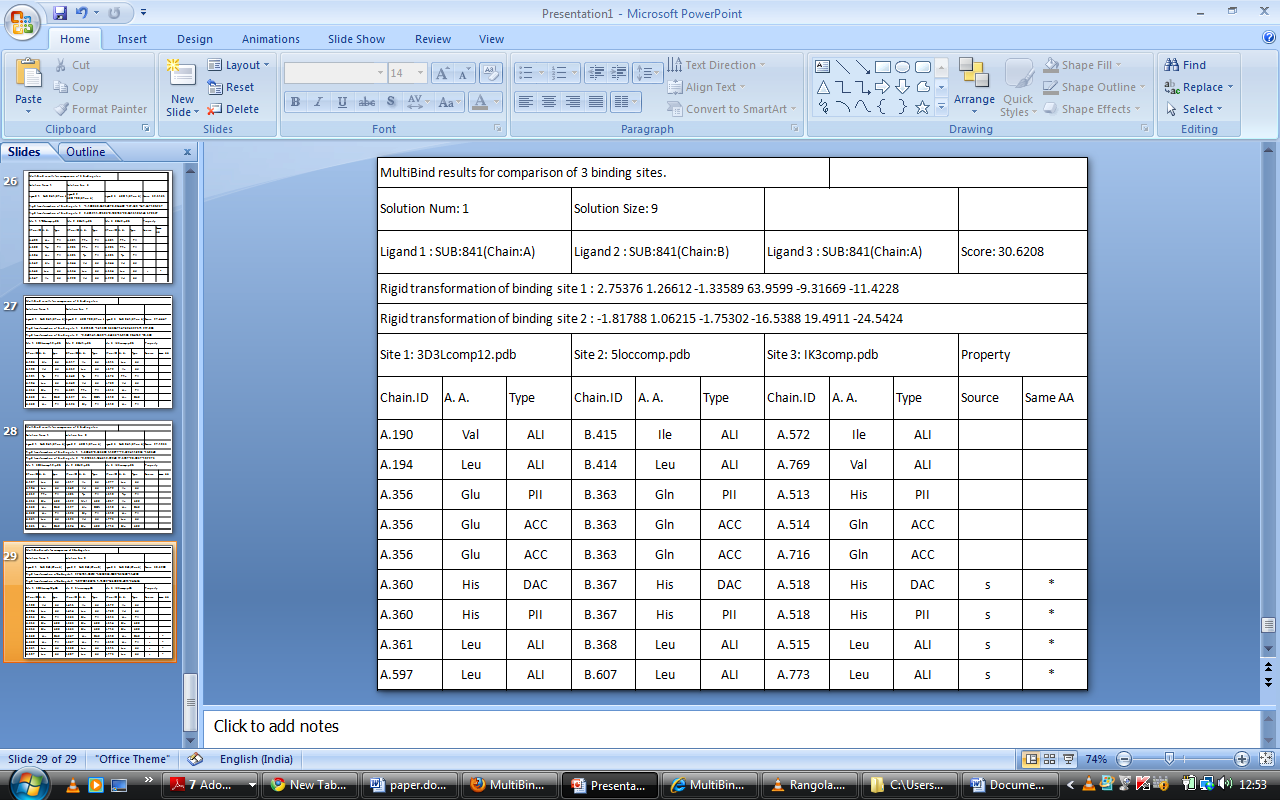


**30)12LOX-5LOX-COX1**


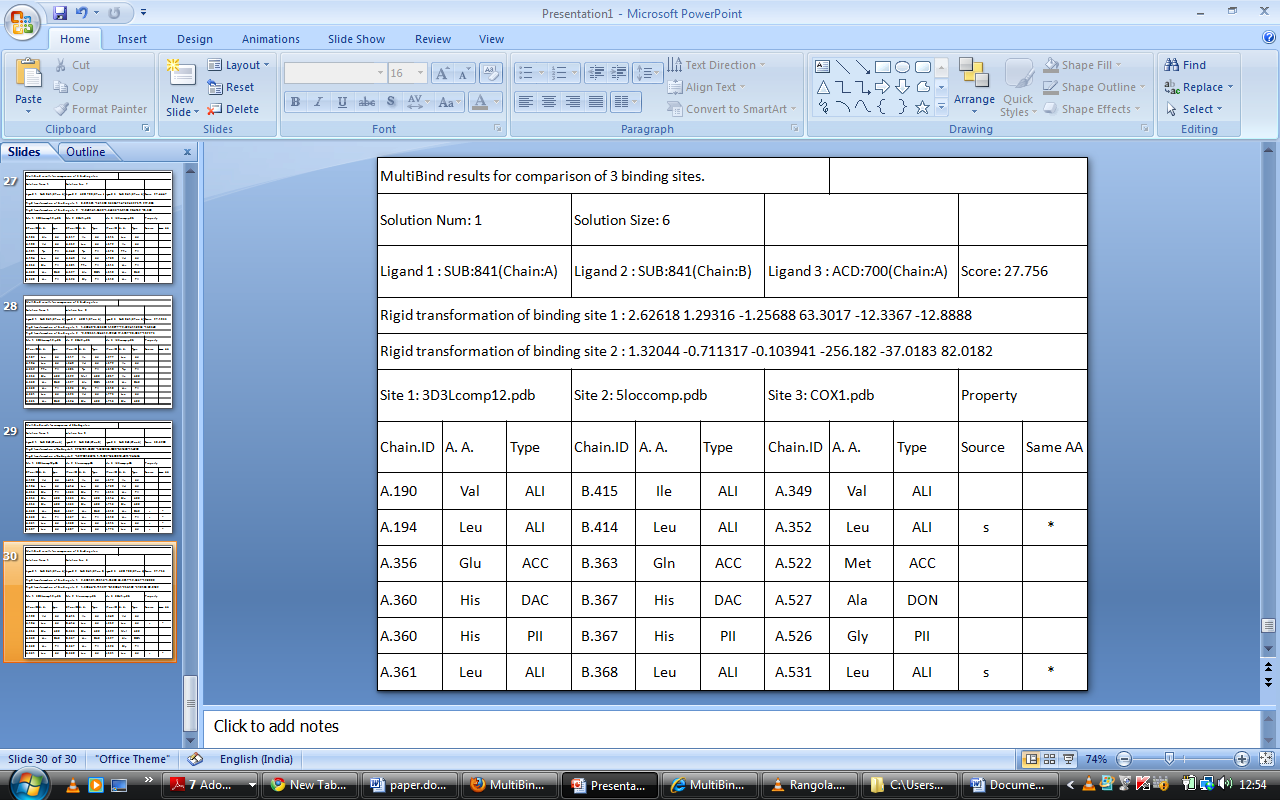


**31)12LOX-5LOX-COX2**


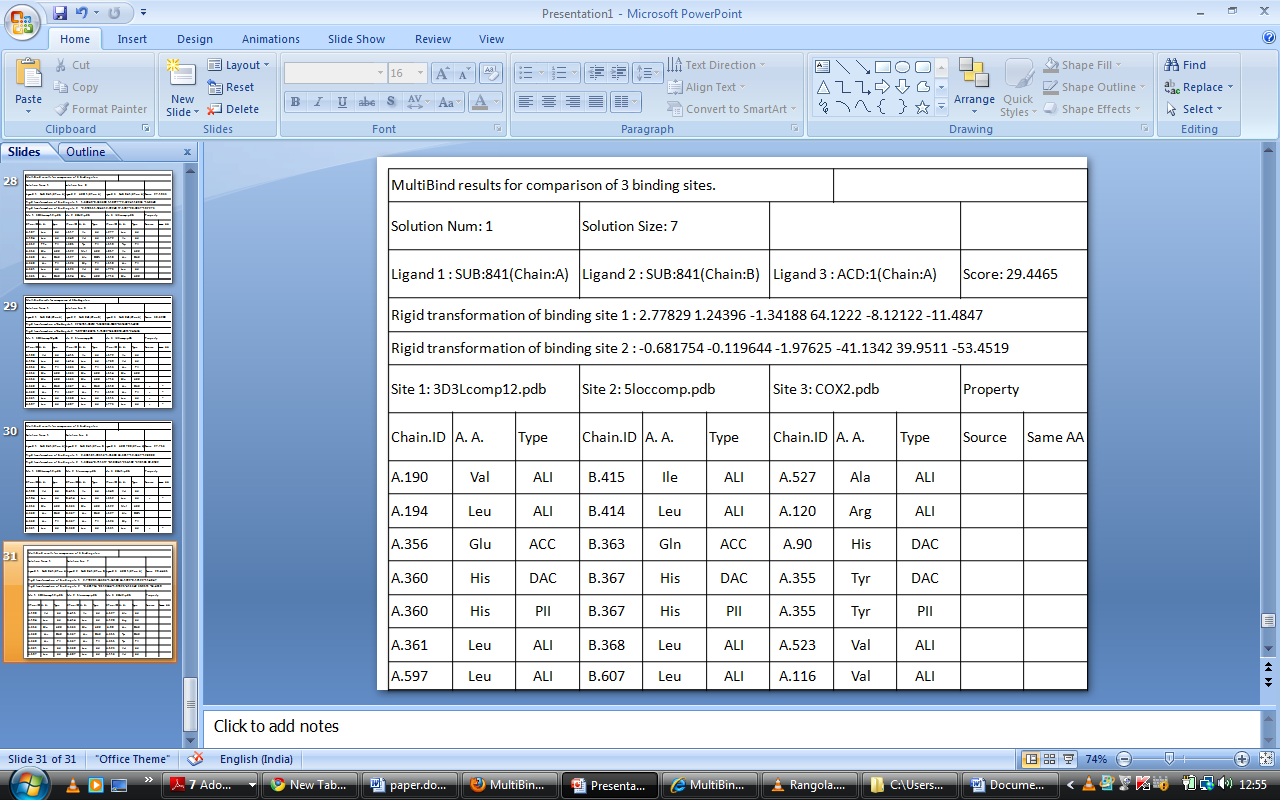


**32)12LOX-COX1-COX2**


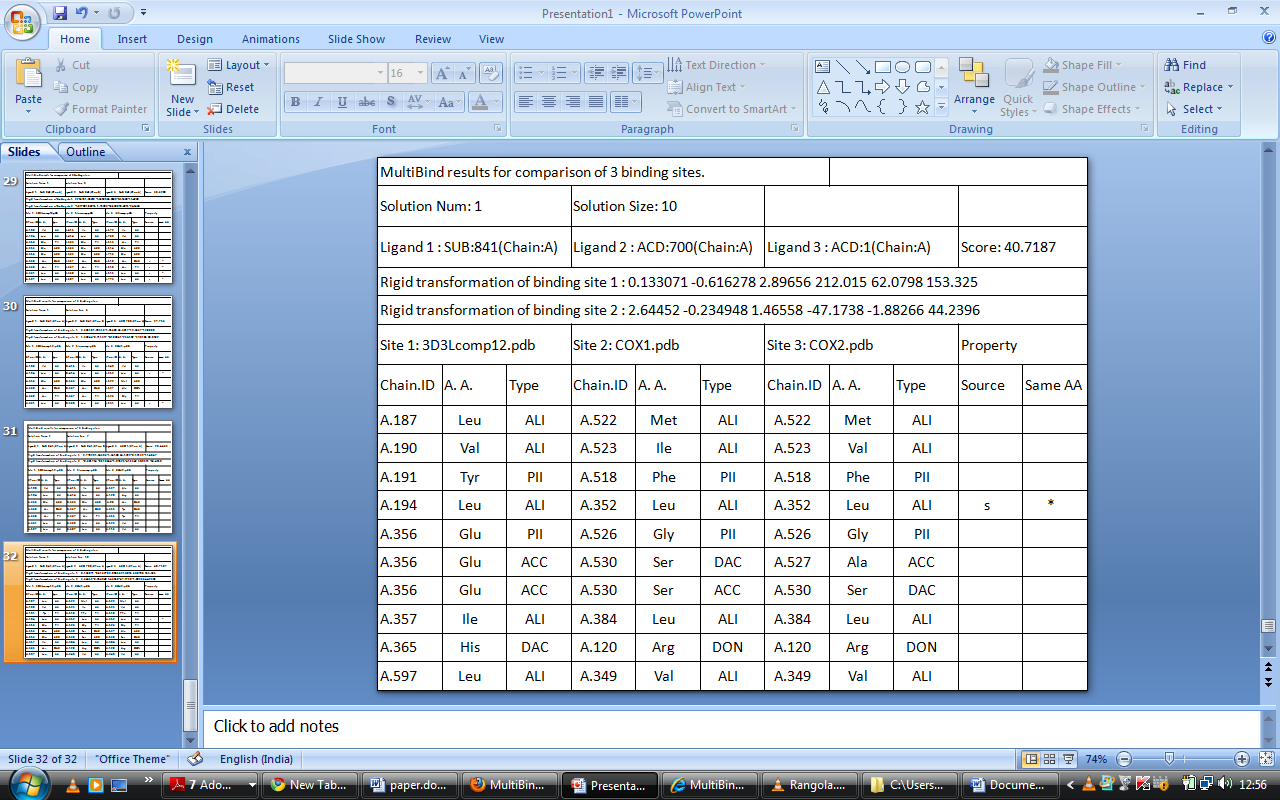


**33)5LOX-COX1-sLOX3**


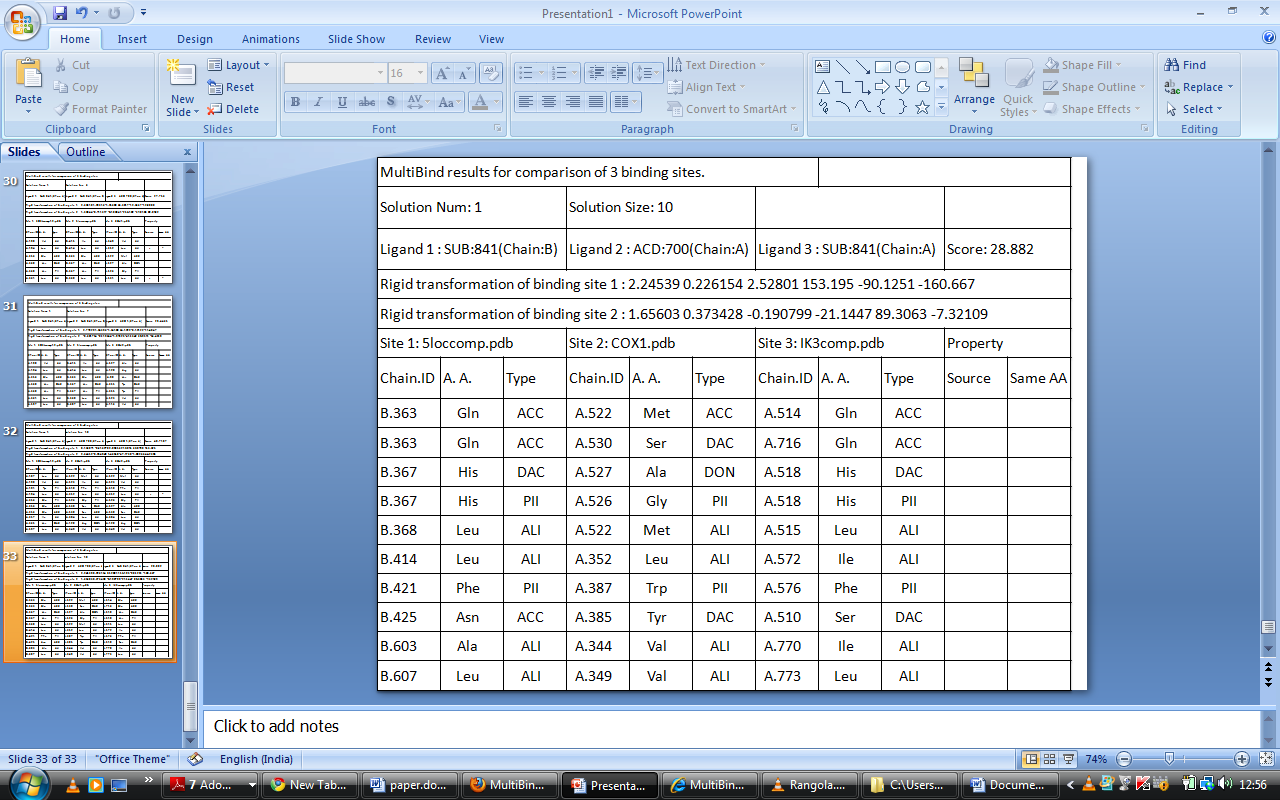


**34)5LOX-COX2-sLOX3**


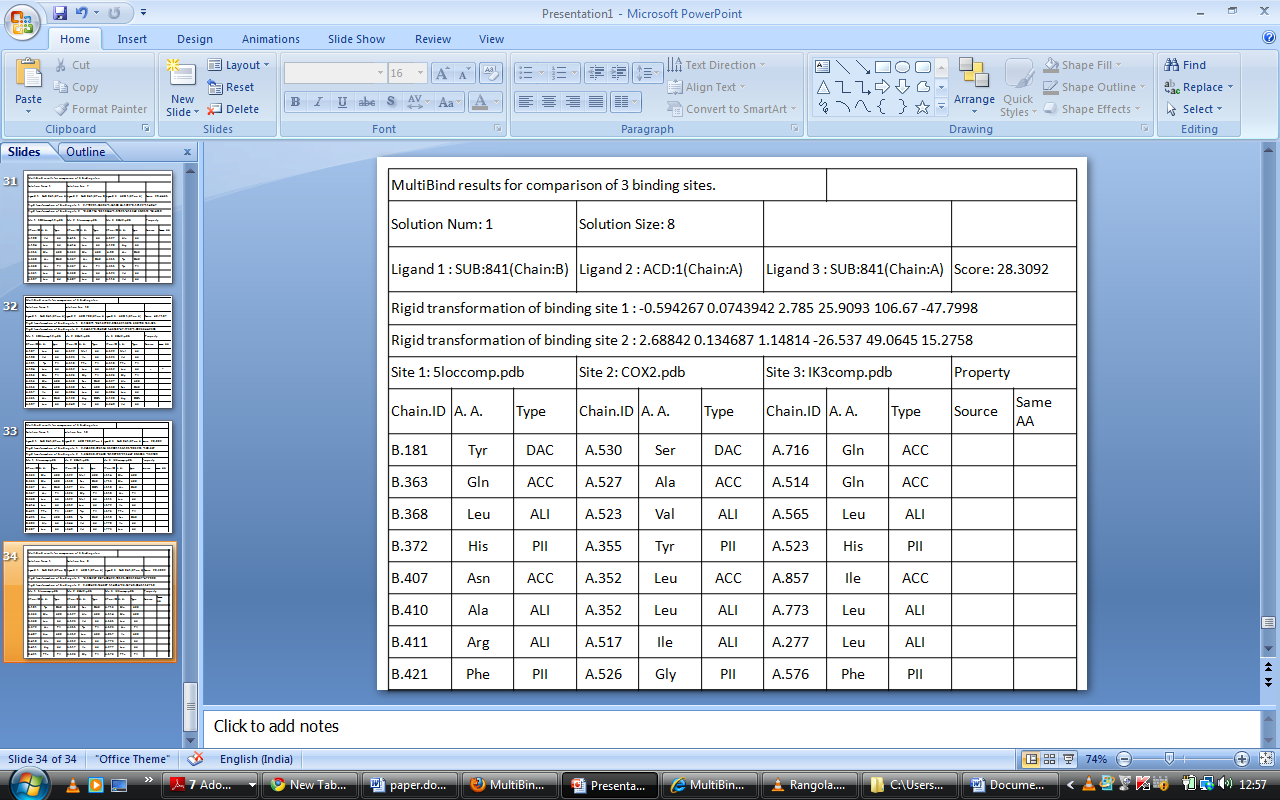


**35)5LOX-COX1-COX2**


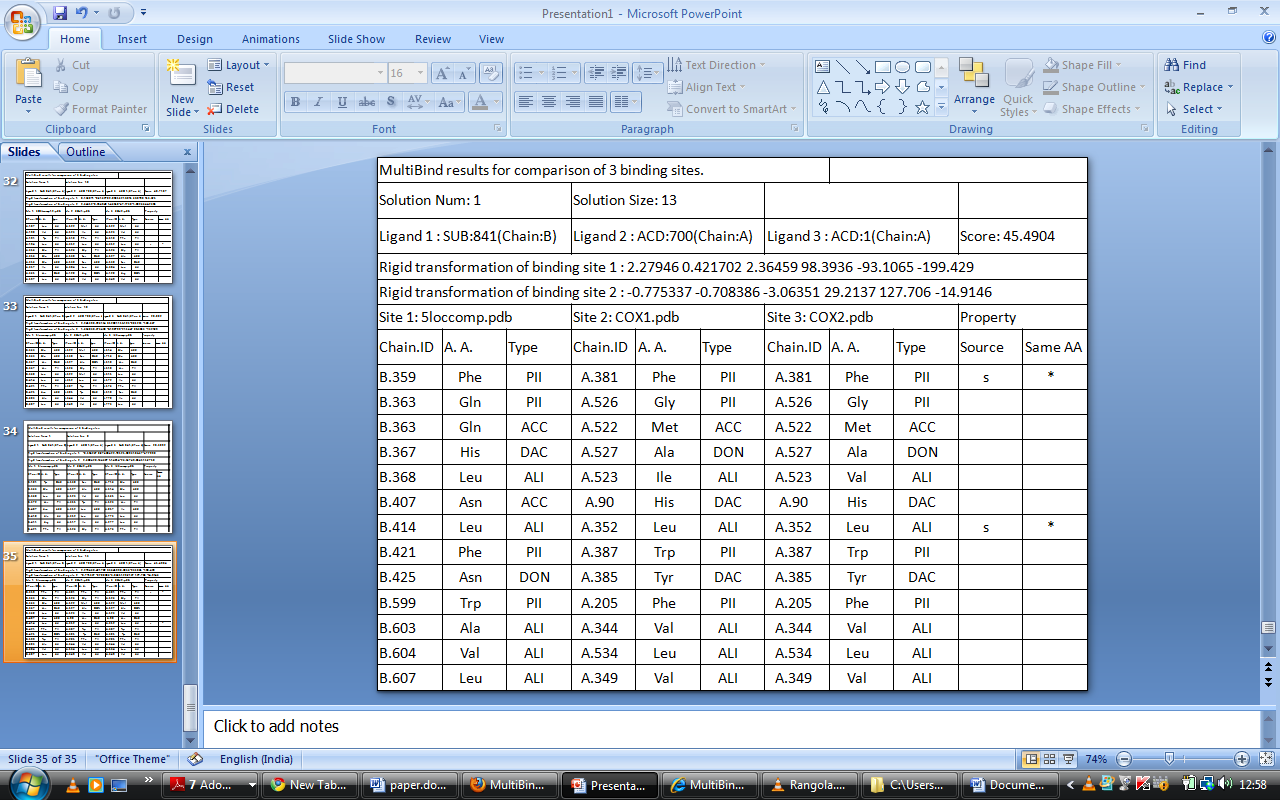


**MULTIPLE ALIGNMENTS OF 4 BINDING SITES:**

The alignment between 4 binding sites produced a total of 35 combinations. The number of common properties varies between 7 and 3. As the binding sites were aligned, eliminating one enzyme at a time from the MULTIBIND during run time, the variation in the number of common physiochemical properties increased.

|  | **COMPARED PROTEINS** | **NO. OF DETECTED FEATURES** | **SCORE** |
| --- | --- | --- | --- |
| **1)** | 15LOX-12LOX-COX1-COX2 | 6 | 32.1992 |
| **2)** | 15LOX-sLOX1-COX2-sLOX3 | 4 | 21.0419 |
| **3)** | 15LOX-sLOX1-12LOX-5LOX | 6 | 22.426 |
| **4)** | 15LOX-sLOX1-12LOX-COX1 | 7 | 21.5979 |
| **5)** | 15LOX-sLOX1-12LOX-COX2 | 4 | 22.9849 |
| **6)** | 15LOX-sLOX1-5LOX-sLOX3 | 6 | 22.8312 |
| **7)** | 15LOX-sLOX1-5LOX-COX1 | 6 | 21.7349 |
| **8)** | 15LOX-sLOX1-5LOX-COX2 | 4 | 20.2278 |
| **9)** | 15LOX-sLOX1-COX1-sLOX3 | 6 | 22.9125 |
| **10)** | 15LOX-sLOX1-COX1-COX2 | 5 | 24.6838 |
| **11)** | 15LOX-12LOX-COX1-sLOX3 | 5 | 26.5233 |
| **12)** | 15LOX-12LOX-5LOX-sLOX3 | 7 | 28.3614 |
| **13)** | 15LOX-12LOX-5LOX-COX1 | 6 | 26.7169 |
| **14)** | 15LOX-sLOX1-5LOX-COX2 | 5 | 27.2331 |
| **15)** | 15LOX-12LOX-COX2-sLOX3 | 6 | 27.527 |
| **16)** | 15LOX-5LOX-COX1-sLOX3 | 6 | 26.197 |
| **17)** | 15LOX-12LOX-5LOX-COX1 | 6 | 26.7169 |
| **18)** | 15LOX-5LOX-COX2-sLOX3 | 8 | 30.4808 |
| **19)** | 15LOX-5LOX-COX1-COX2 | 7 | 32.0864 |
| **20)** | 15LOX-COX1-COX2-sLOX3 | 6 | 30.2231 |
| **21)** | sLOX1-COX2-COX1-sLOX3 | 7 | 28.8762 |
| **22)** | sLOX1-12LOX-5LOX-sLOX3 | 3 | 17.1475 |
| **23)** | sLOX1-12LOX-5LOX-COX1 | 3 | 17.1472 |
| **24)** | sLOX1-12LOX-5LOX-COX2 | 4 | 19.1022 |
| **25)** | sLOX1-12LOX-COX1-sLOX3 | 4 | 18.3007 |
| **26)** | sLOX1-12LOX-COX1-COX2 | 4 | 23.2816 |
| **27)** | sLOX1-12LOX-COX2-sLOX3 | 4 | 18.2793 |
| **28)** | sLOX1-5LOX-COX1-sLOX3 | 6 | 22.4107 |
| **29)** | sLOX1-5LOX-COX1-COX2 | 5 | 24.8696 |
| **30)** | sLOX1-5LOX-COX2-sLOX3 | 7 | 23.563 |
| **31)** | 12LOX-5LOX-COX1-sLOX3 | 7 | 26.8822 |
| **32)** | 12LOX-5LOX-COX2-sLOX3 | 6 | 27.7119 |
| **33)** | 12LOX-COX1-COX2-sLOX3 | 6 | 32.3466 |
| **34)** | 12LOX-5LOX-COX1-COX2 | 5 | 32.4295 |
| **35)** | 5LOX-COX1-COX2-sLOX3 | 5 | 30.0732 |

**1) 15LOX-12LOX-COX1-COX2**


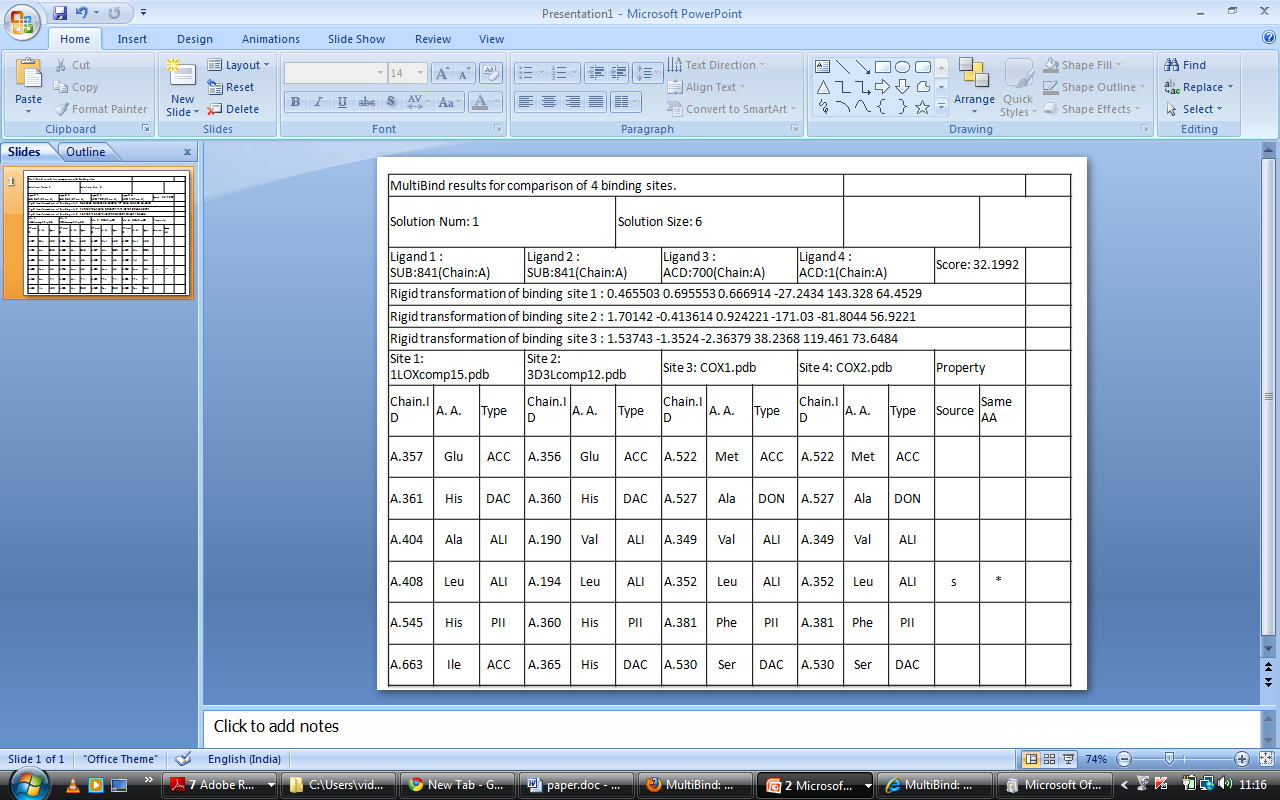


**2)15LOX-sLOX1-COX2-sLOX3**


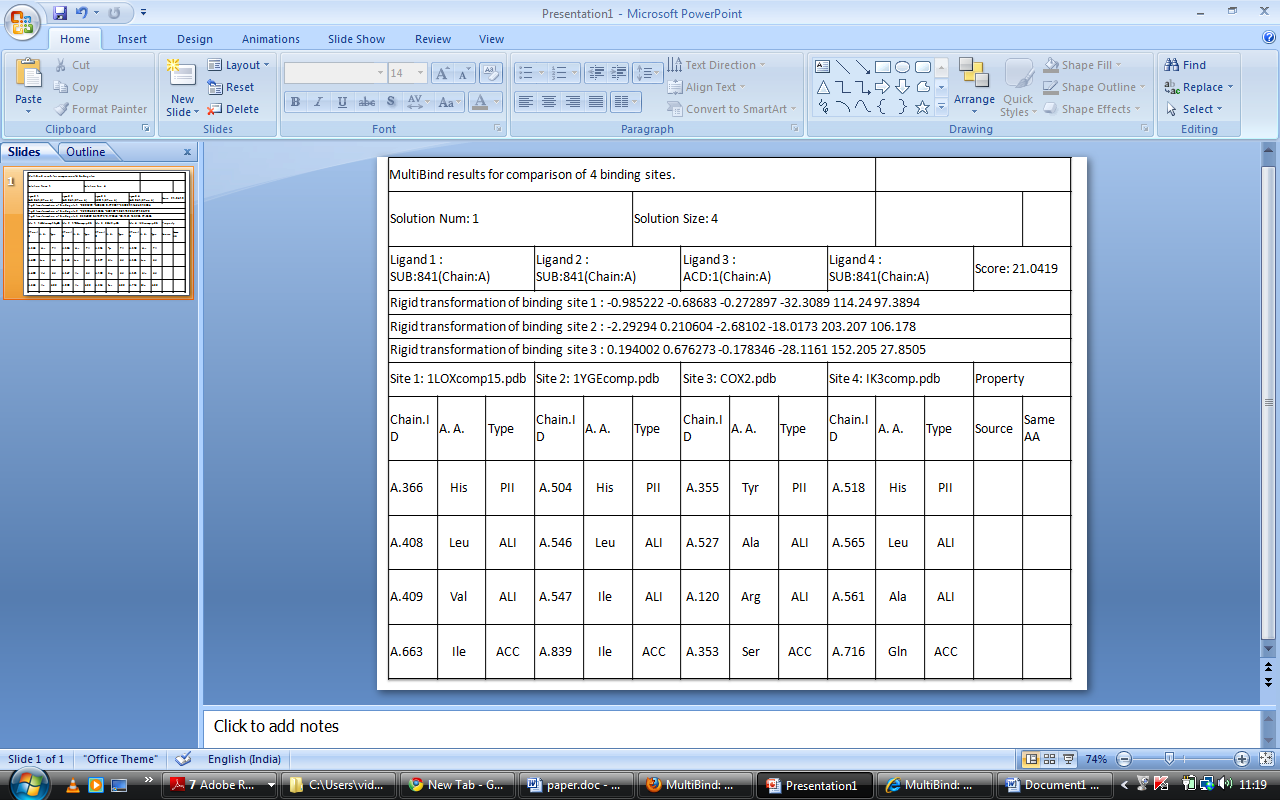


**3)15LOX-sLOX1-12LOX-5LOX**


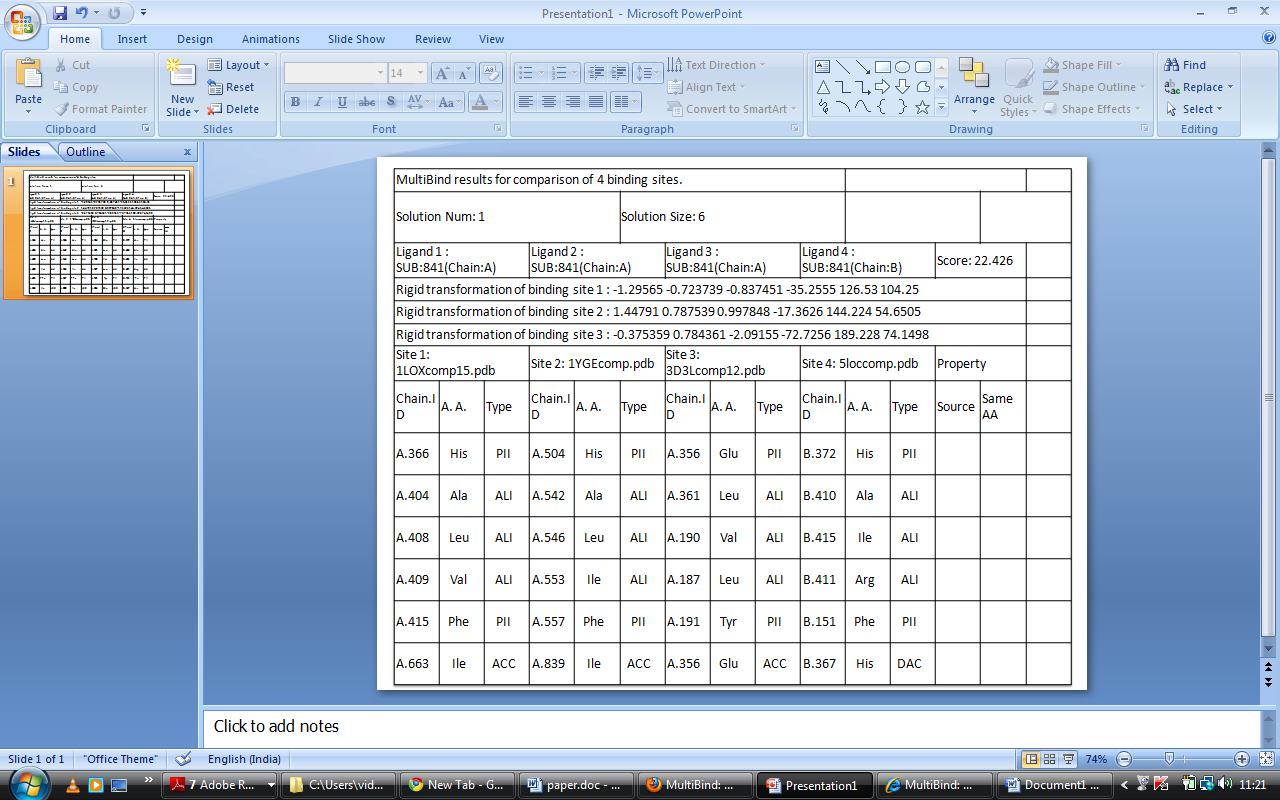


**4) 15LOX-sLOX1-12LOX-5LOX**


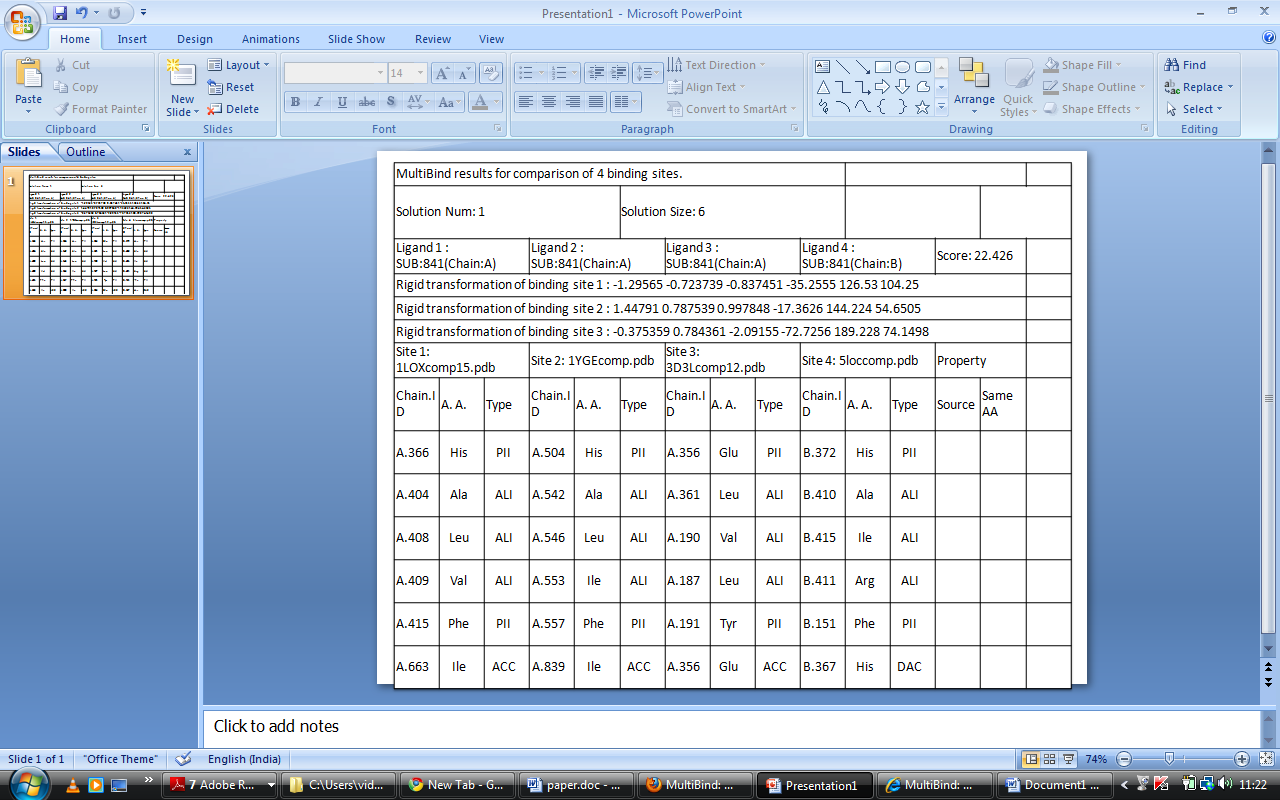


**5)15LOX-sLOX1-12LOX-COX1**


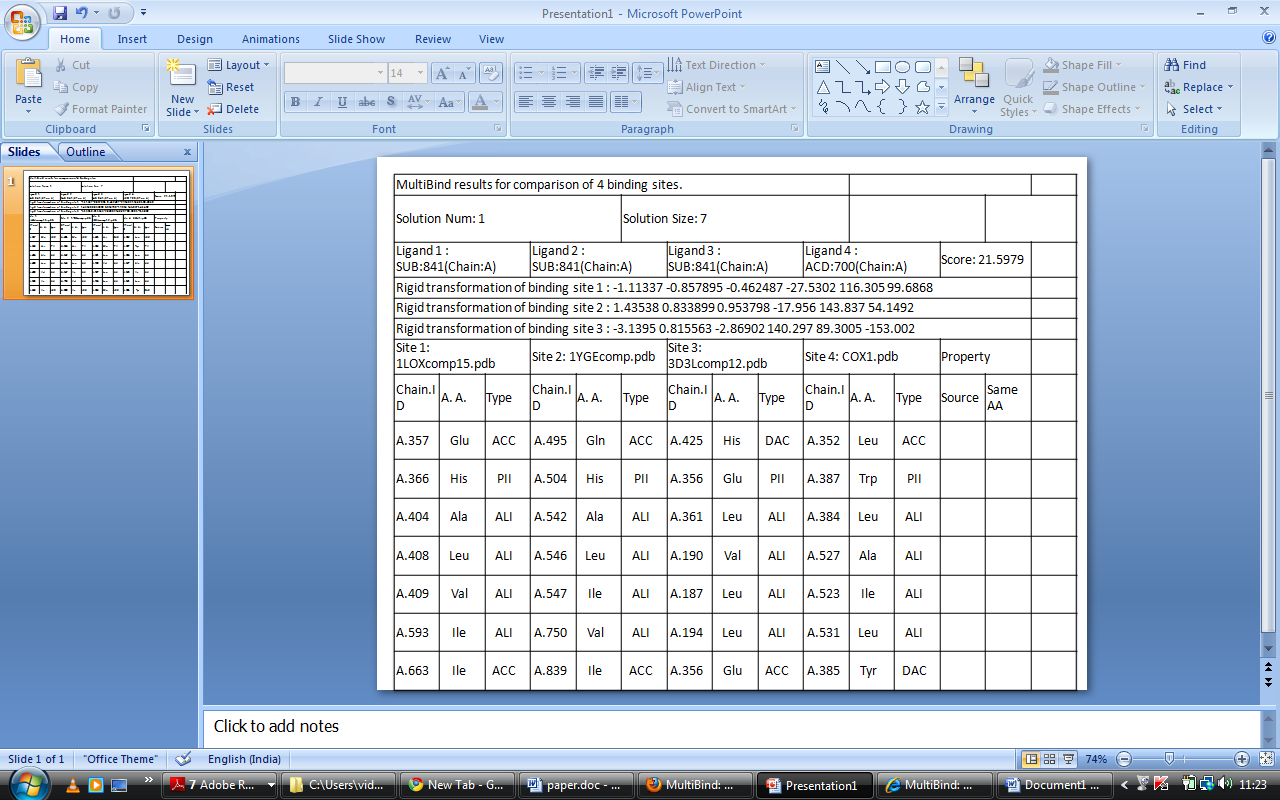


**6) 15LOX-sLOX1-12LOX-COX2**


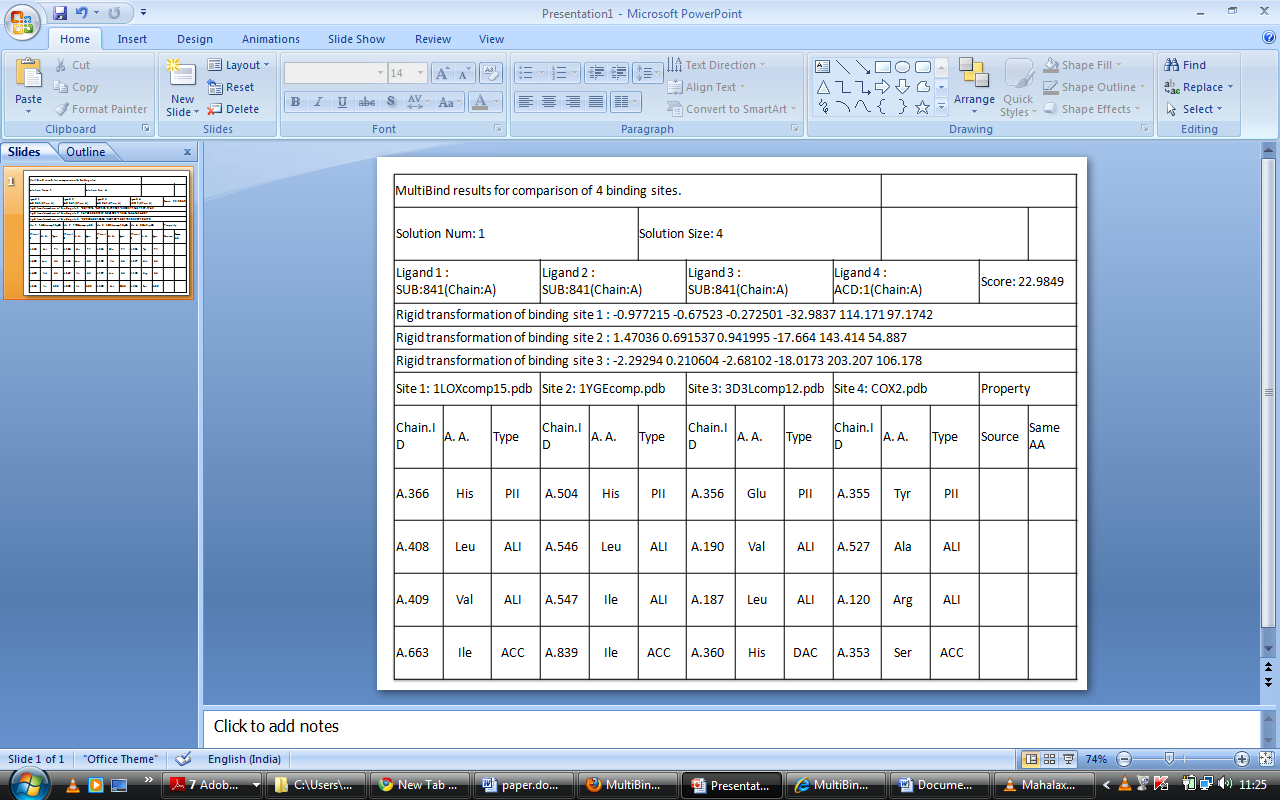


**7)** **15LOX-sLOX1-5LOX-sLOX3**


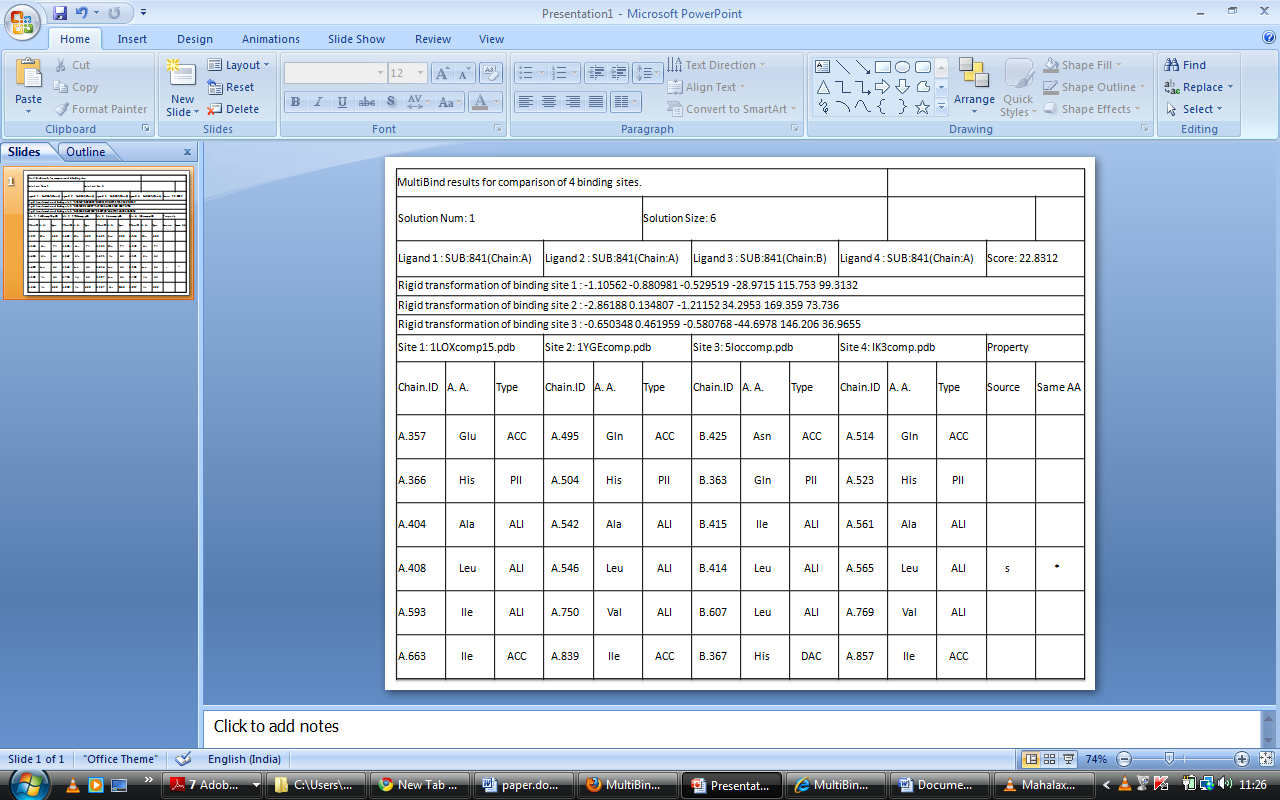


**8) 15LOX-sLOX1-5LOX-COX1**


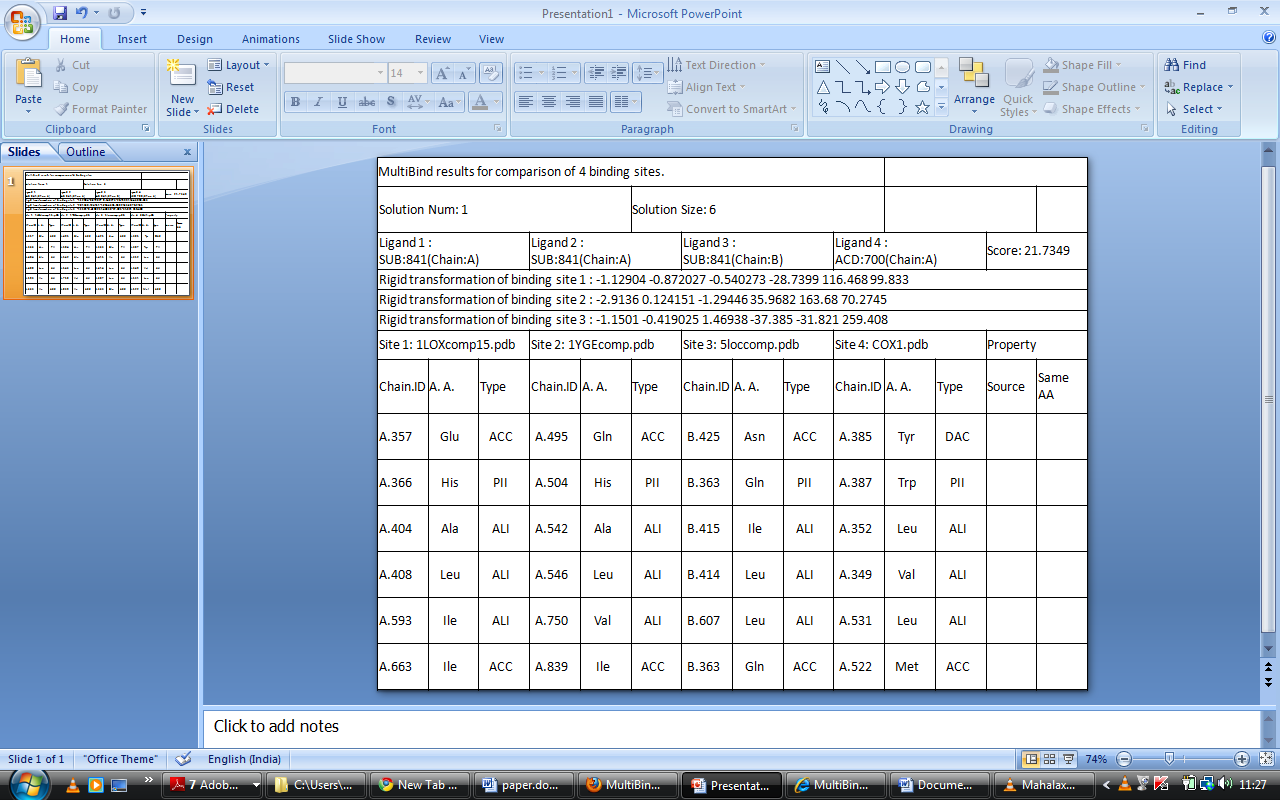


**9) 15LOX-sLOX1-5LOX-COX2**


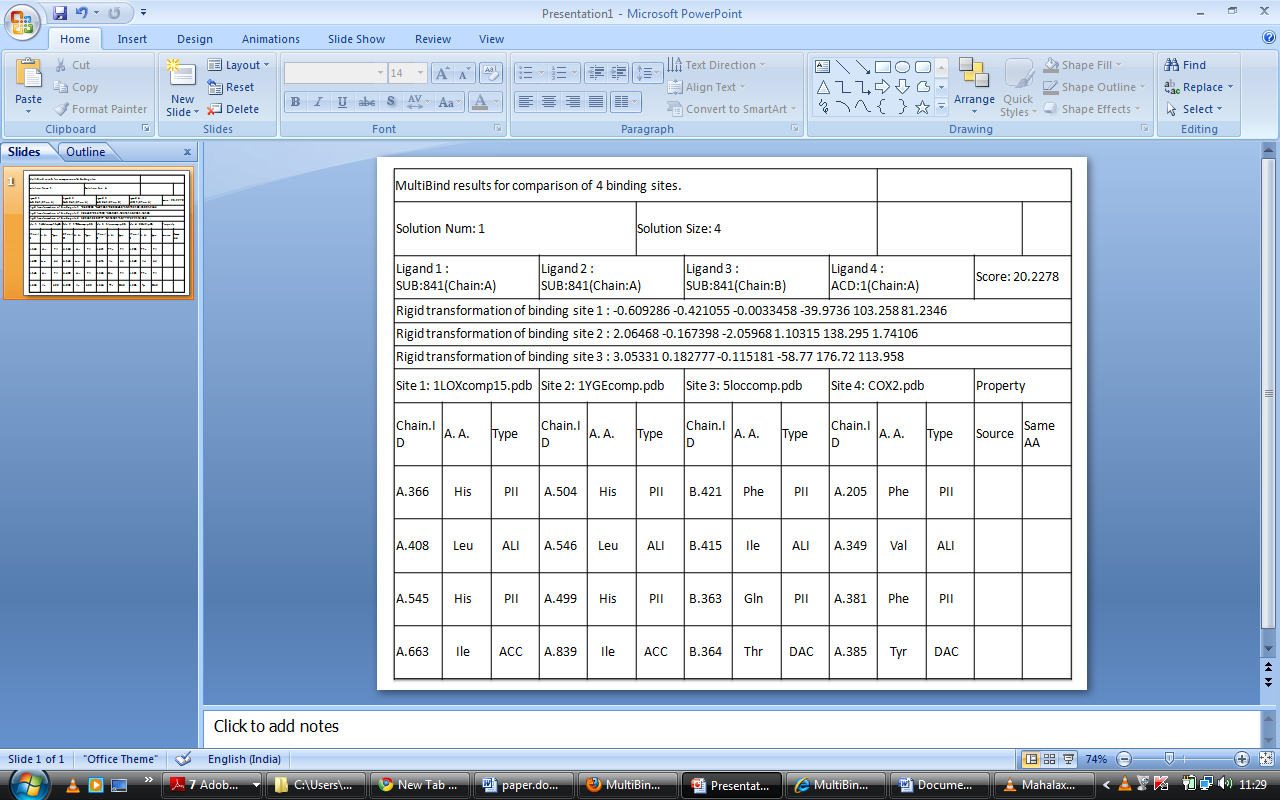


**10) 15LOX-sLOX1-COX1-sLOX3**


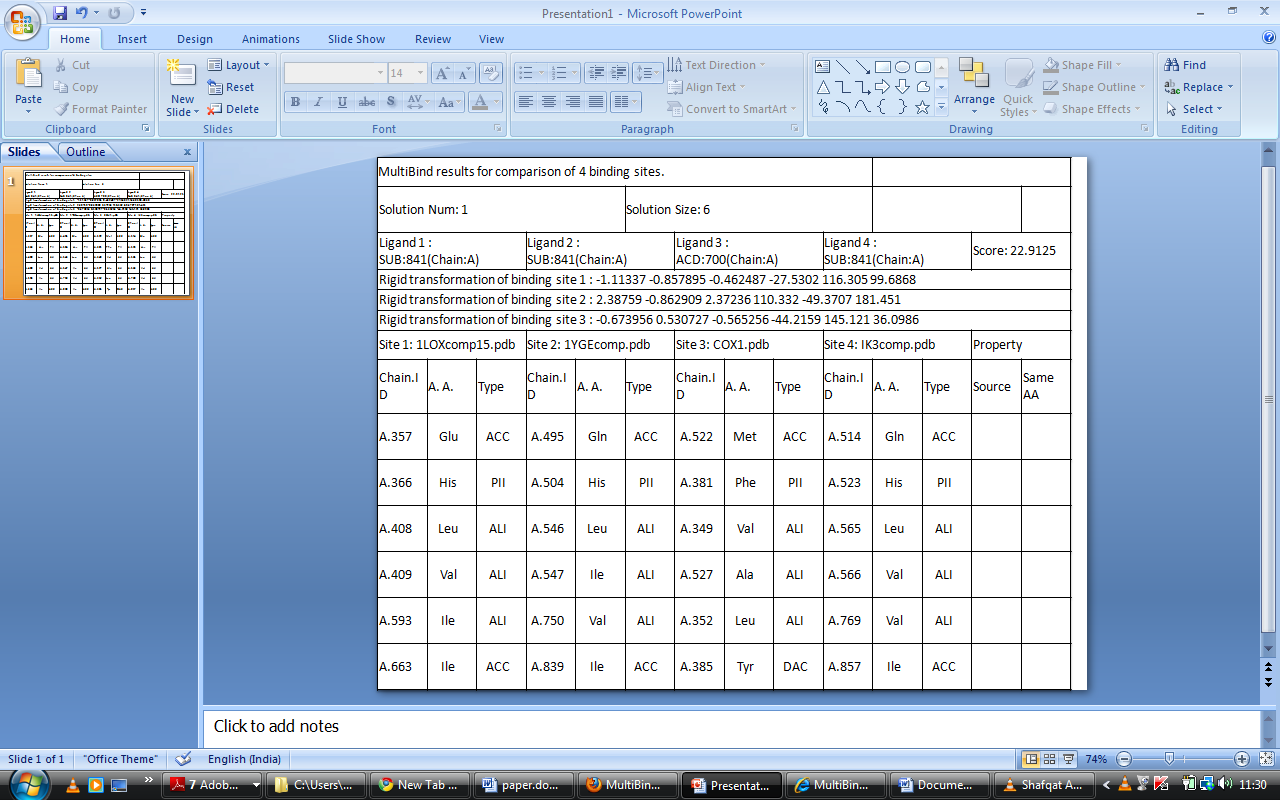


**11) 15LOX-sLOX1-COX1-COX2**


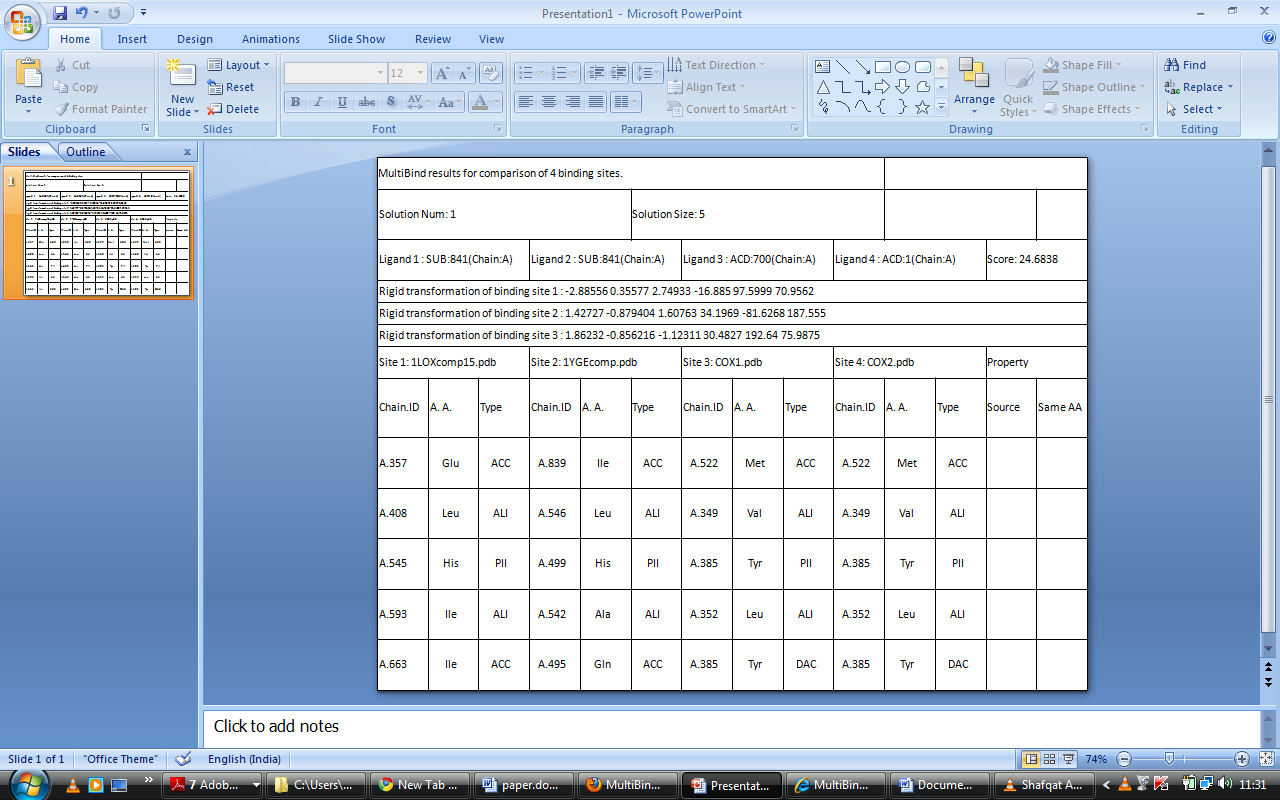


**12) 15LOX-12LOX-COX1-sLOX3**


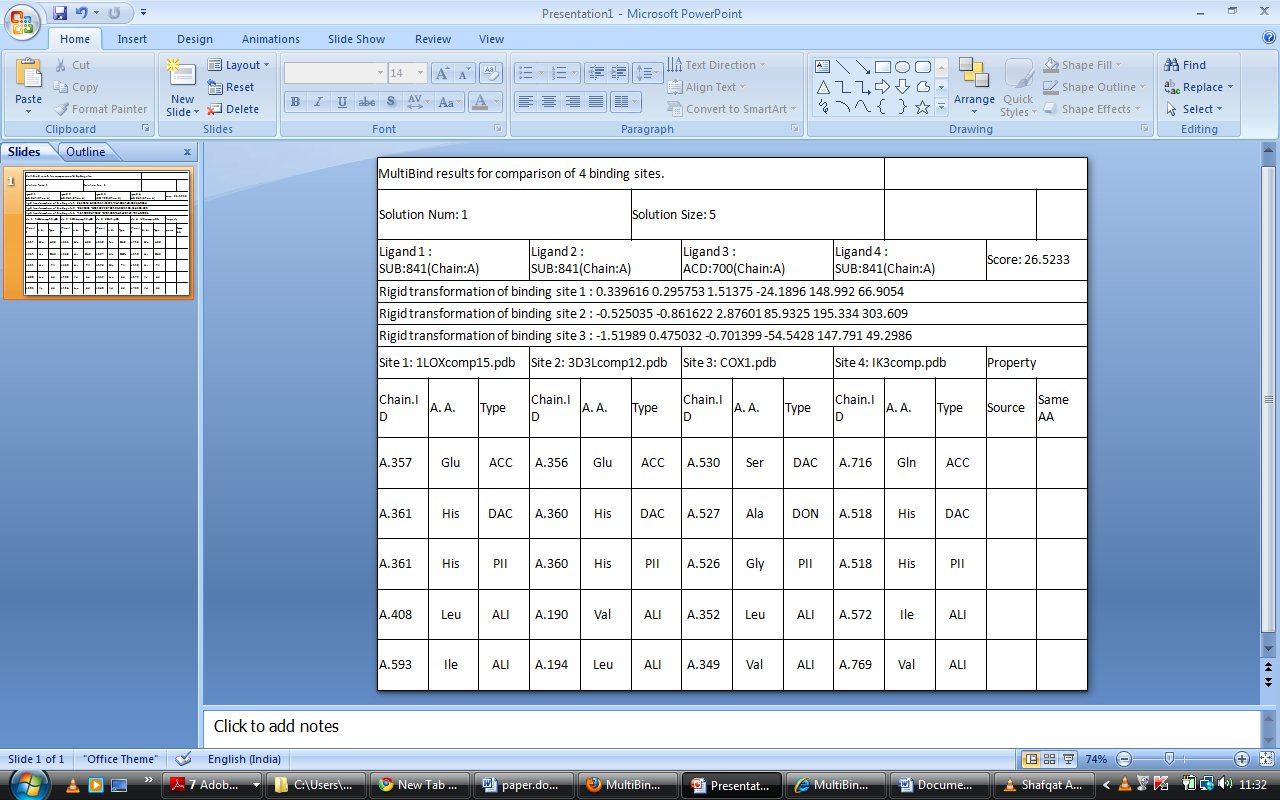


**13)15LOX-12LOX-5LOX-sLOX3**


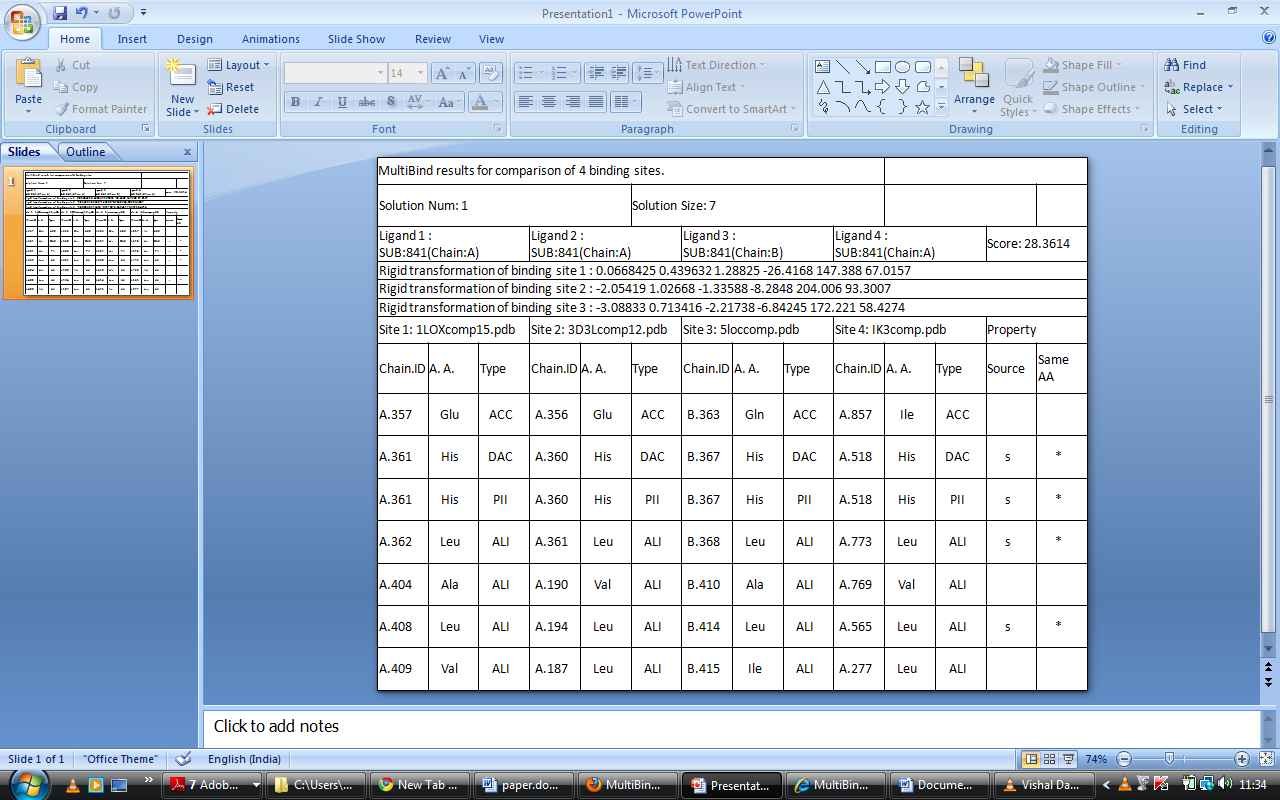


**14) 15LOX-12LOX-5LOX-COX1**


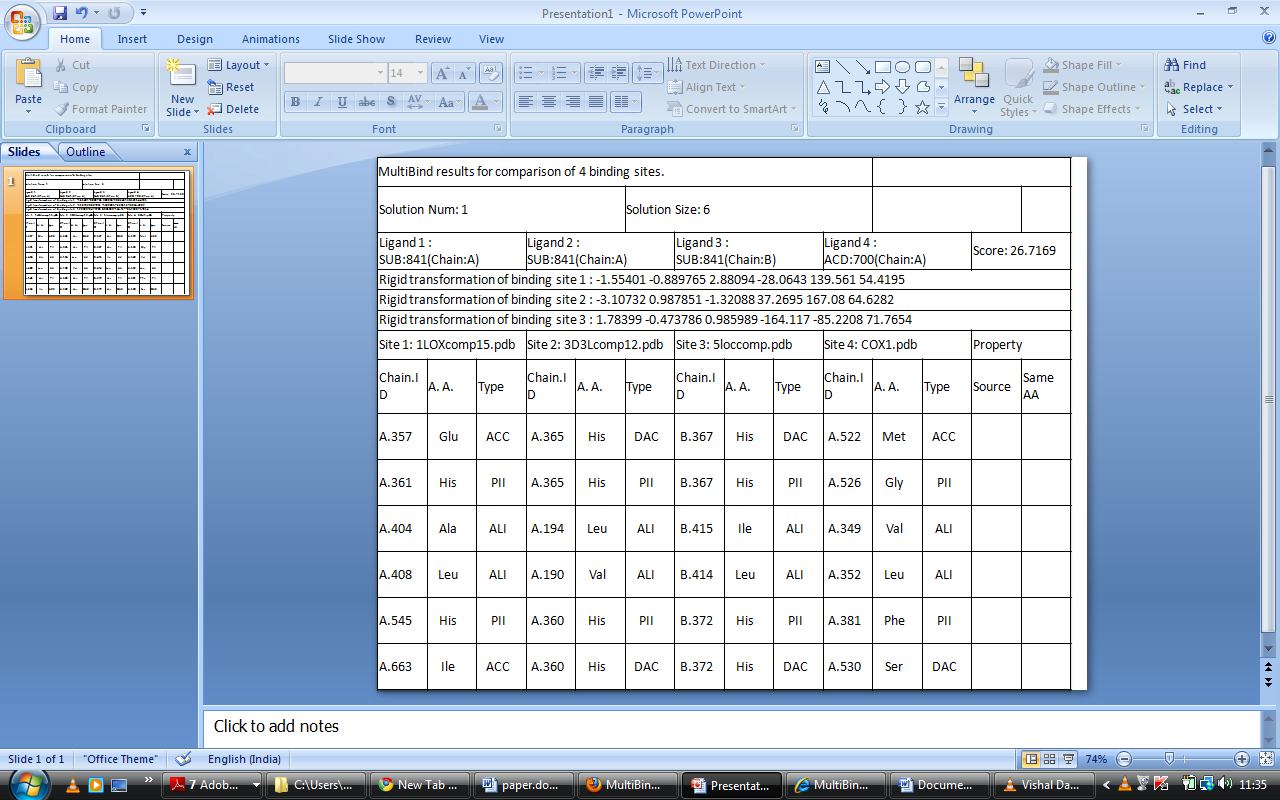


**15) 15LOX-12LOX-5LOX-COX2**


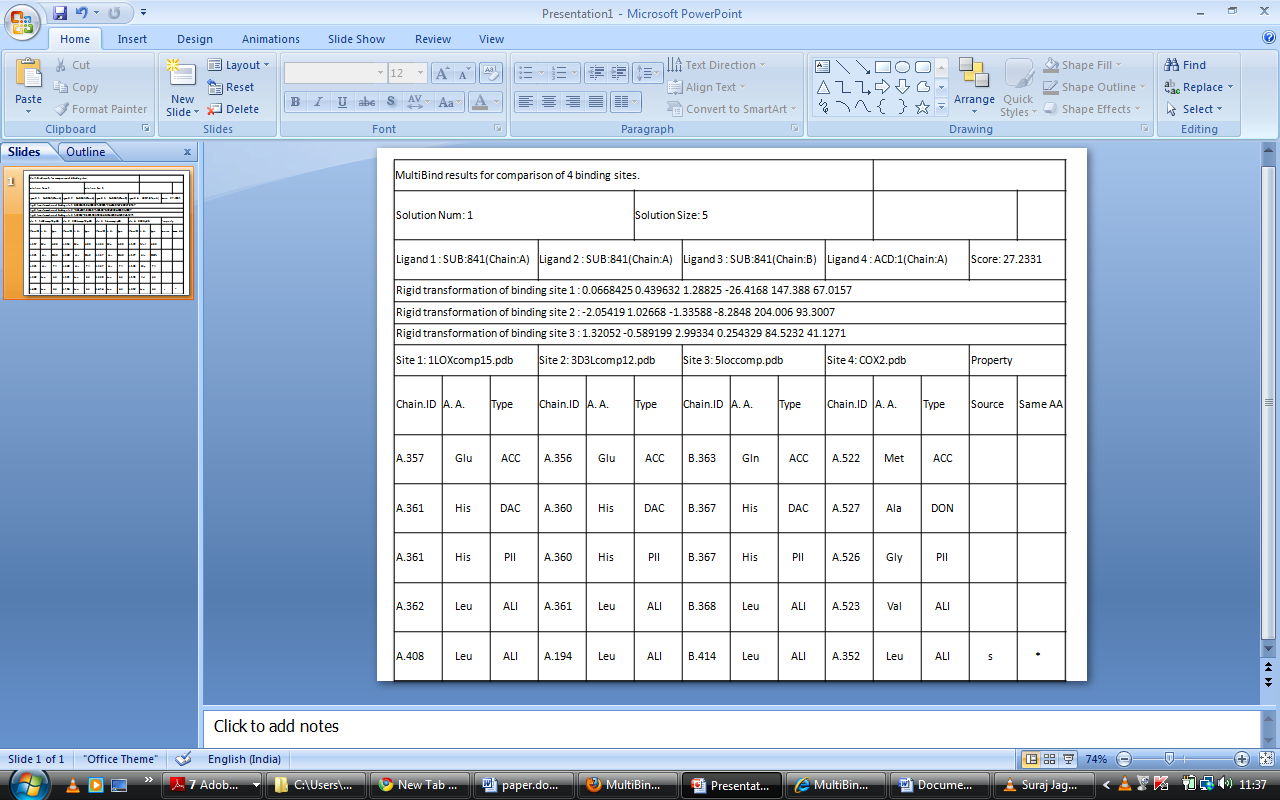


**16)15LOX-12LOX-COX2-sLOX3**


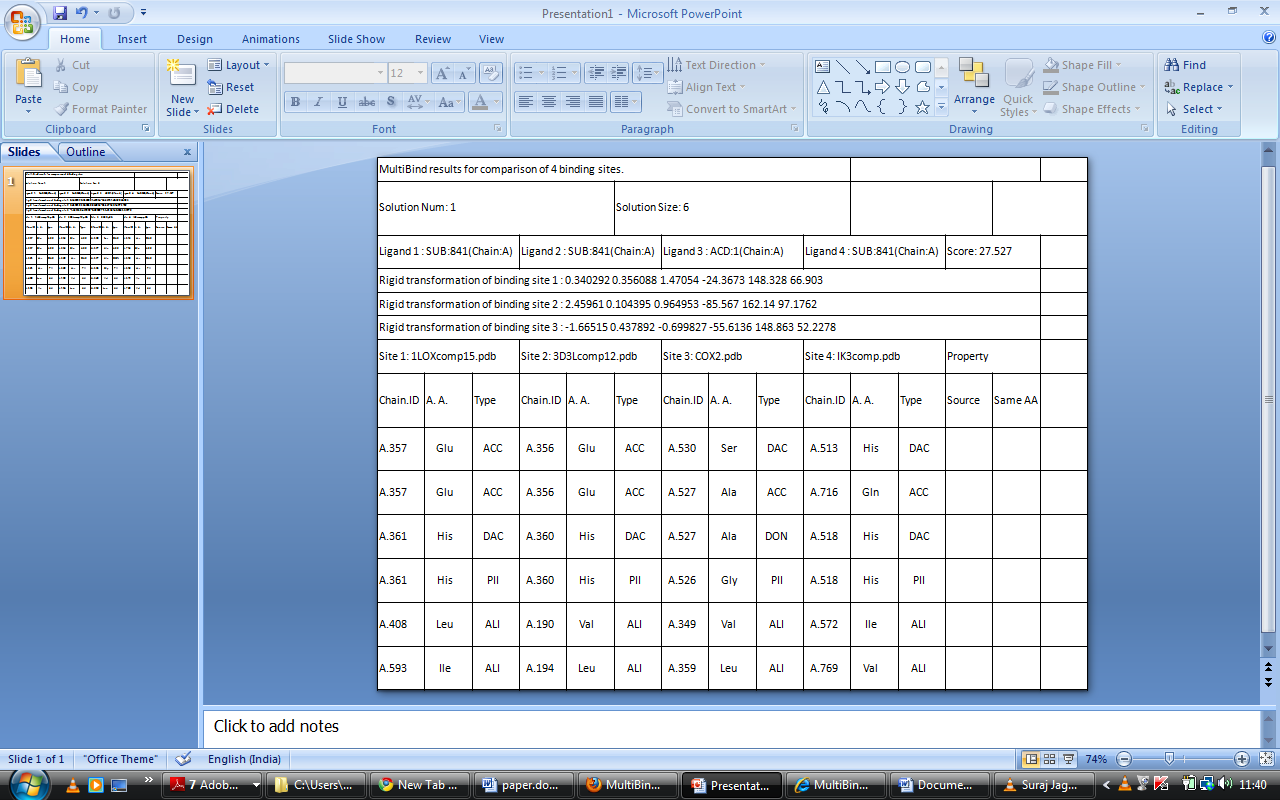


**17)15LOX-5LOX-COX1-sLOX3**


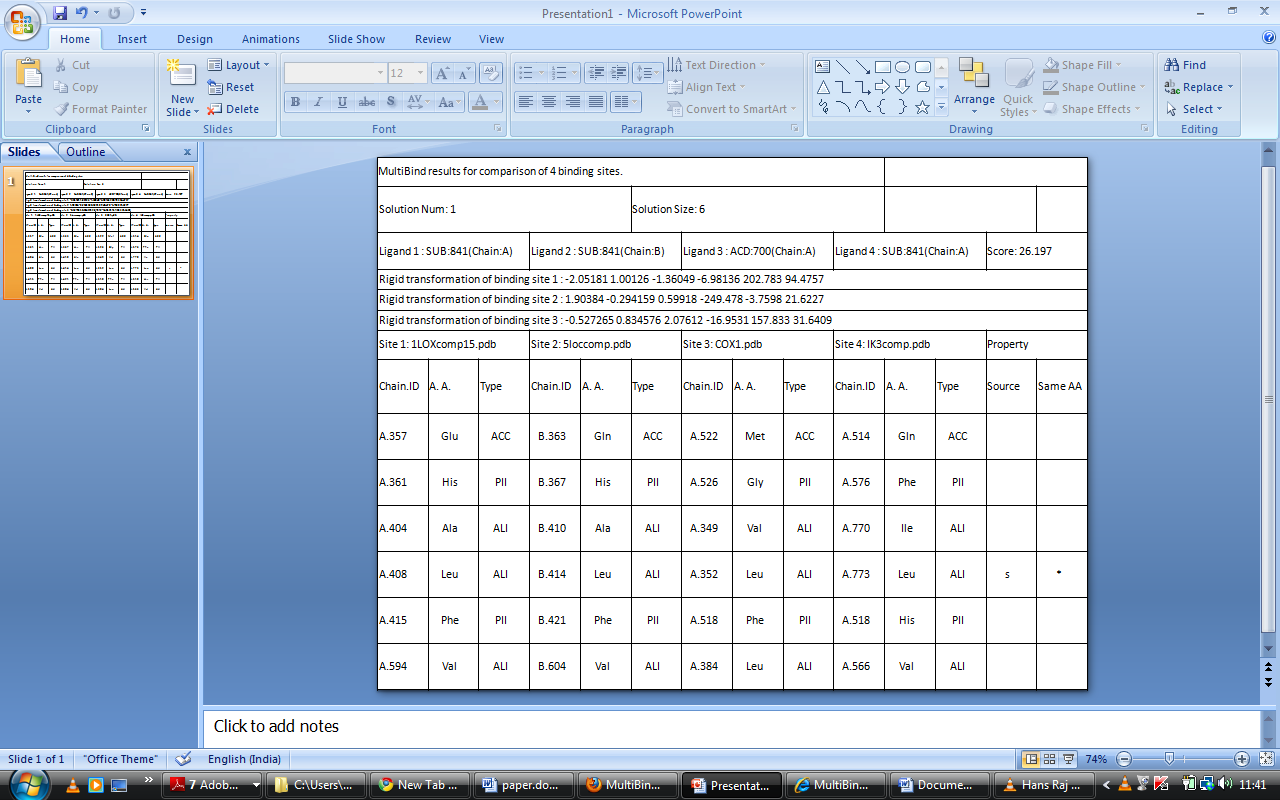


**18)15LOX-12LOX-5LOX-COX1**


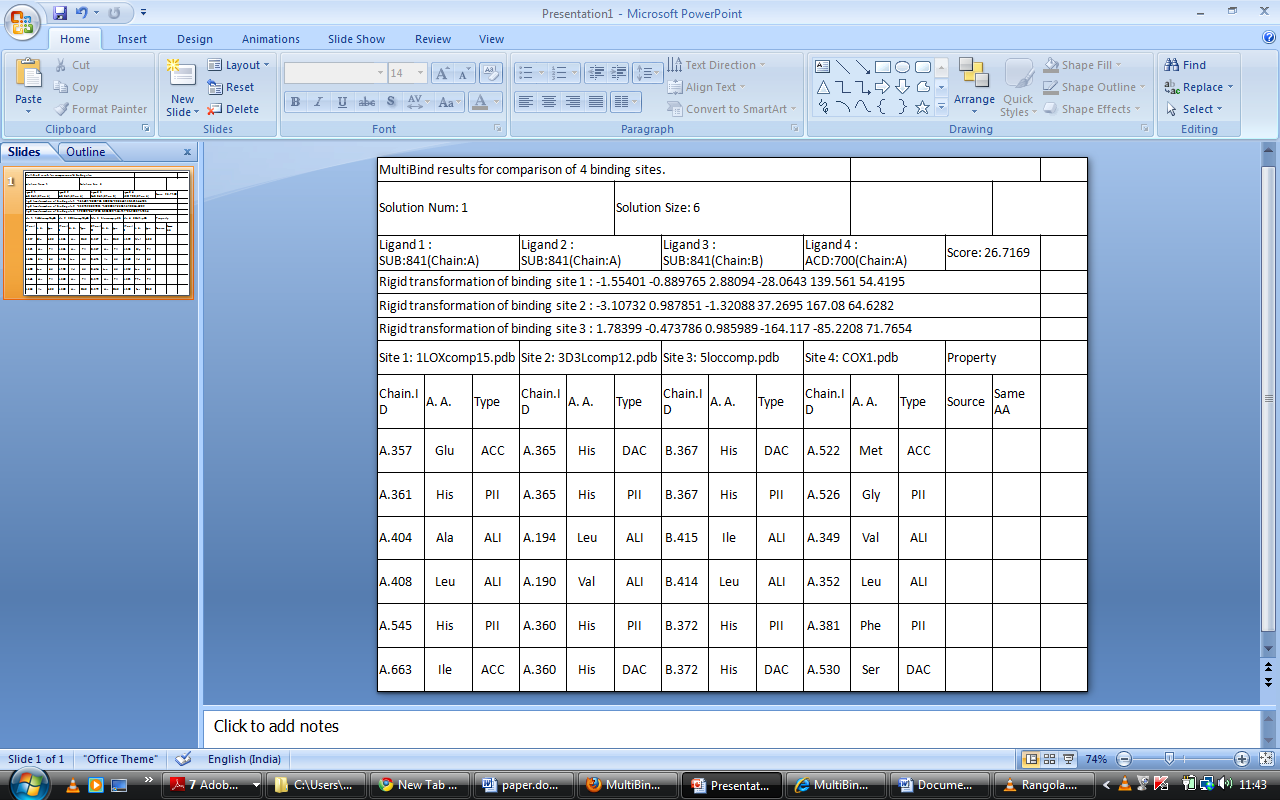


**19)15LOX-5LOX-COX2-sLOX3**


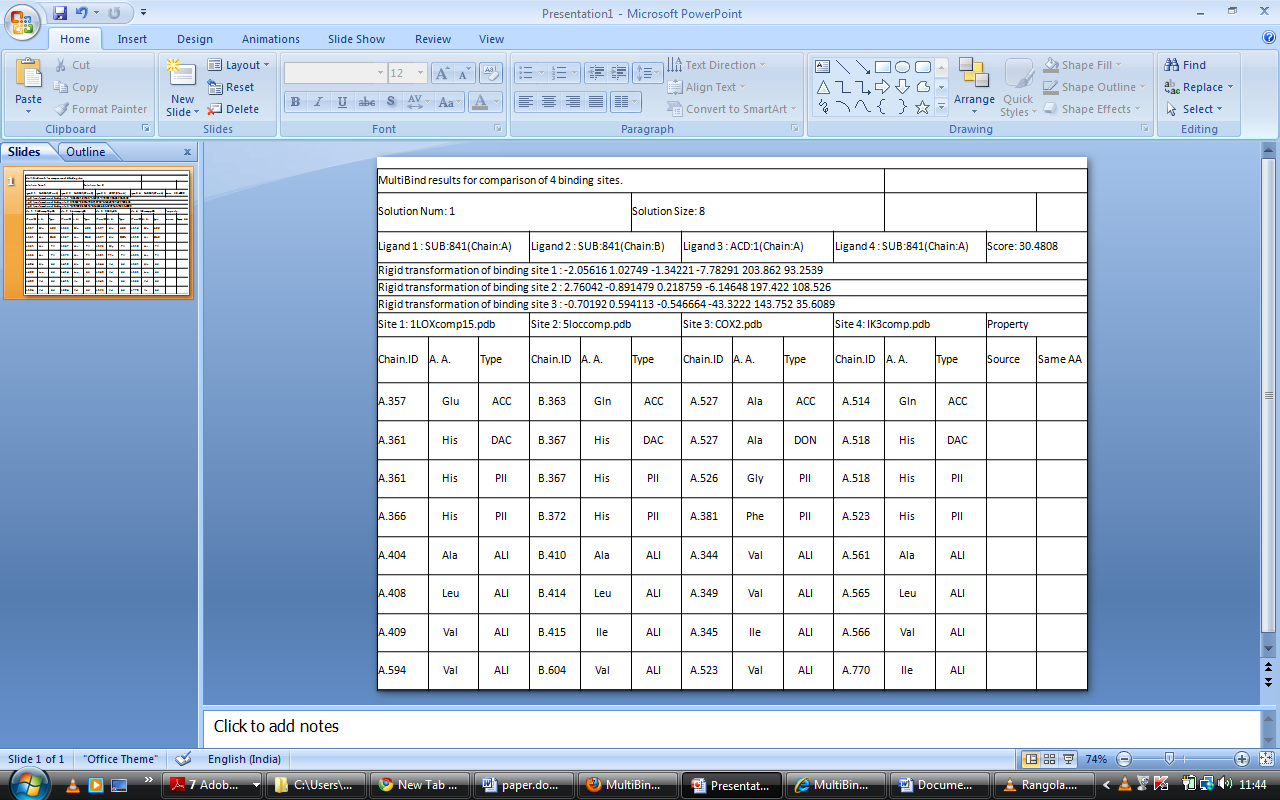


**20) 15LOX-5LOX-COX1-COX2**

*
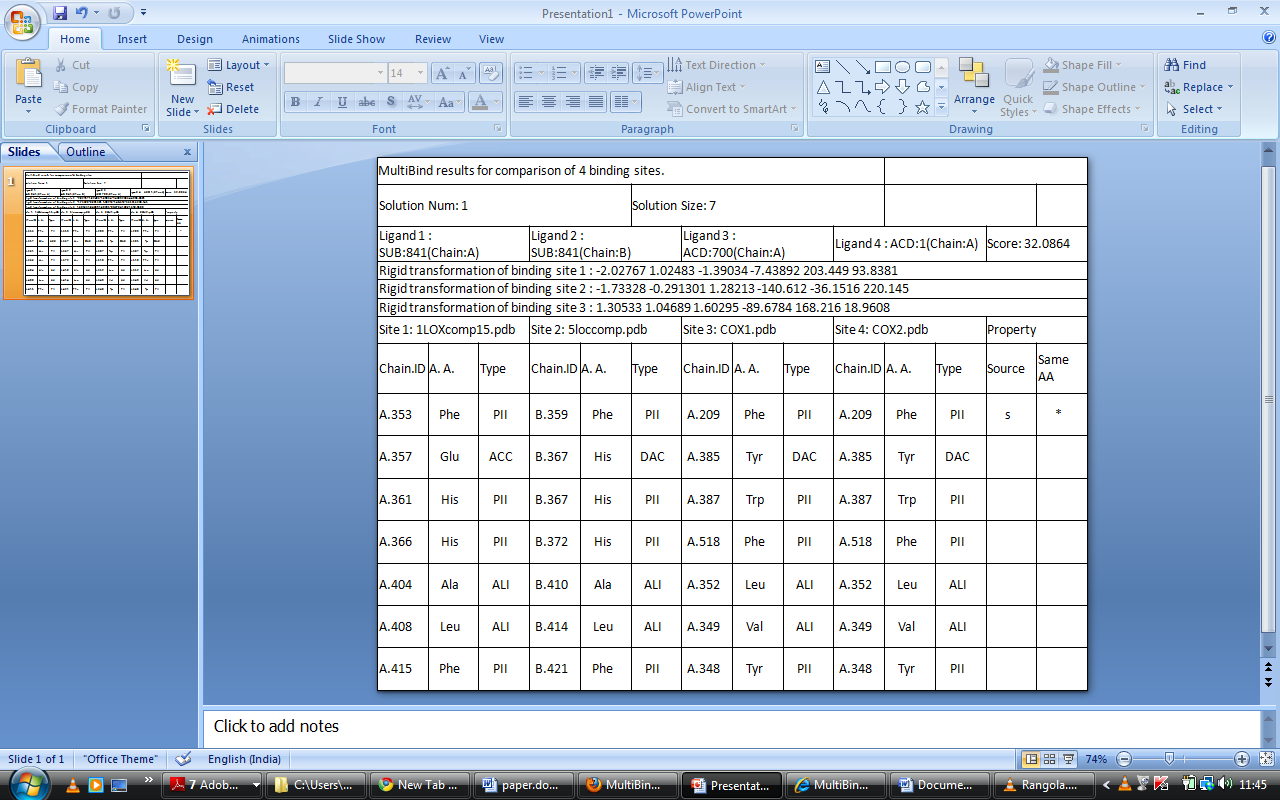
*

**21)15LOX-COX1-COX2-sLOX3**


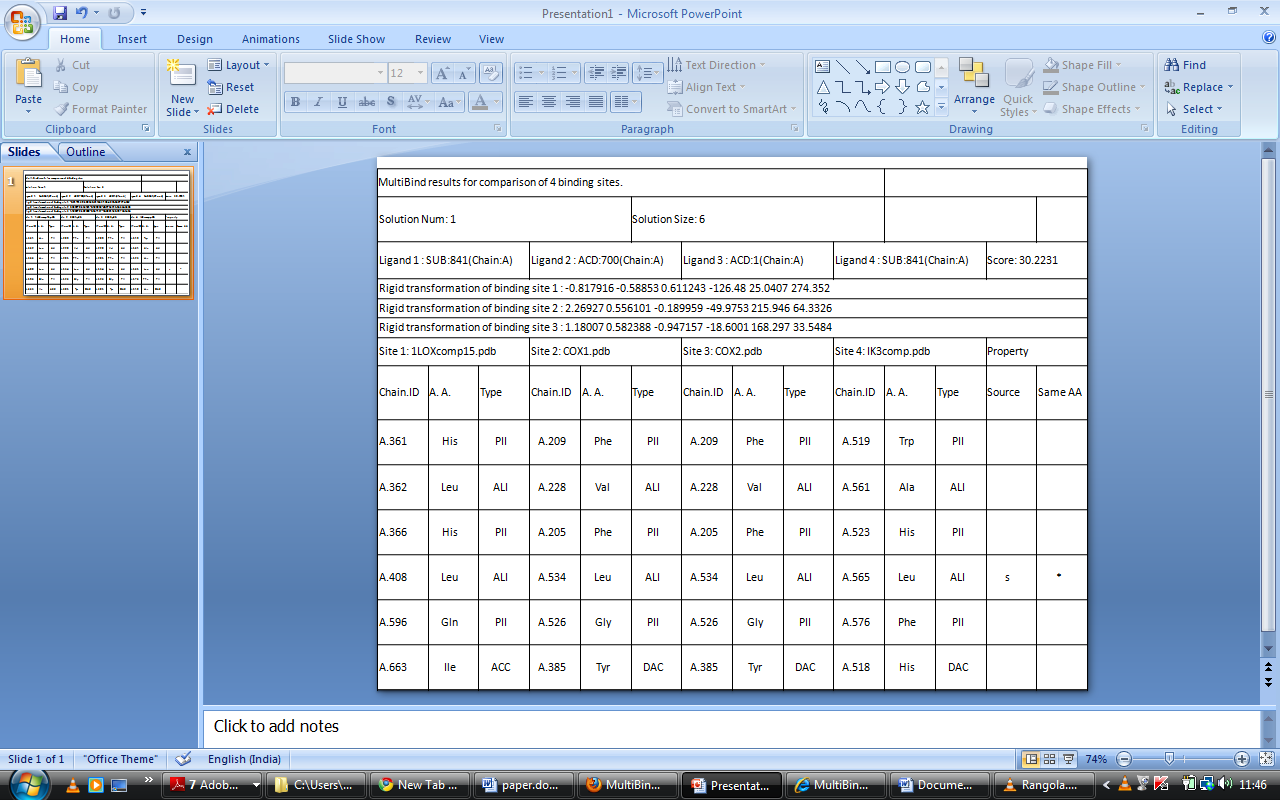


**22) sLOX1-COX1-COX2-sLOX3**


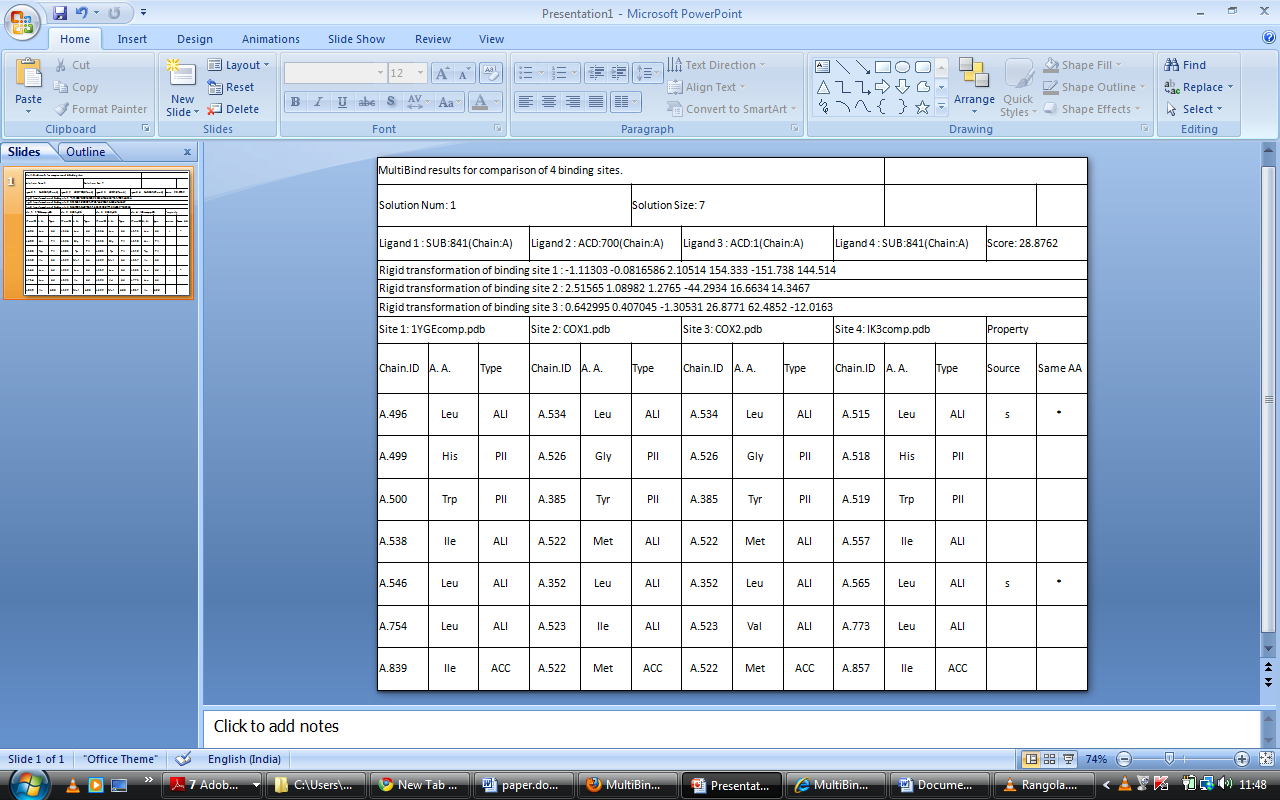


**23) sLOX1-12LOX-5LOX-sLOX3**


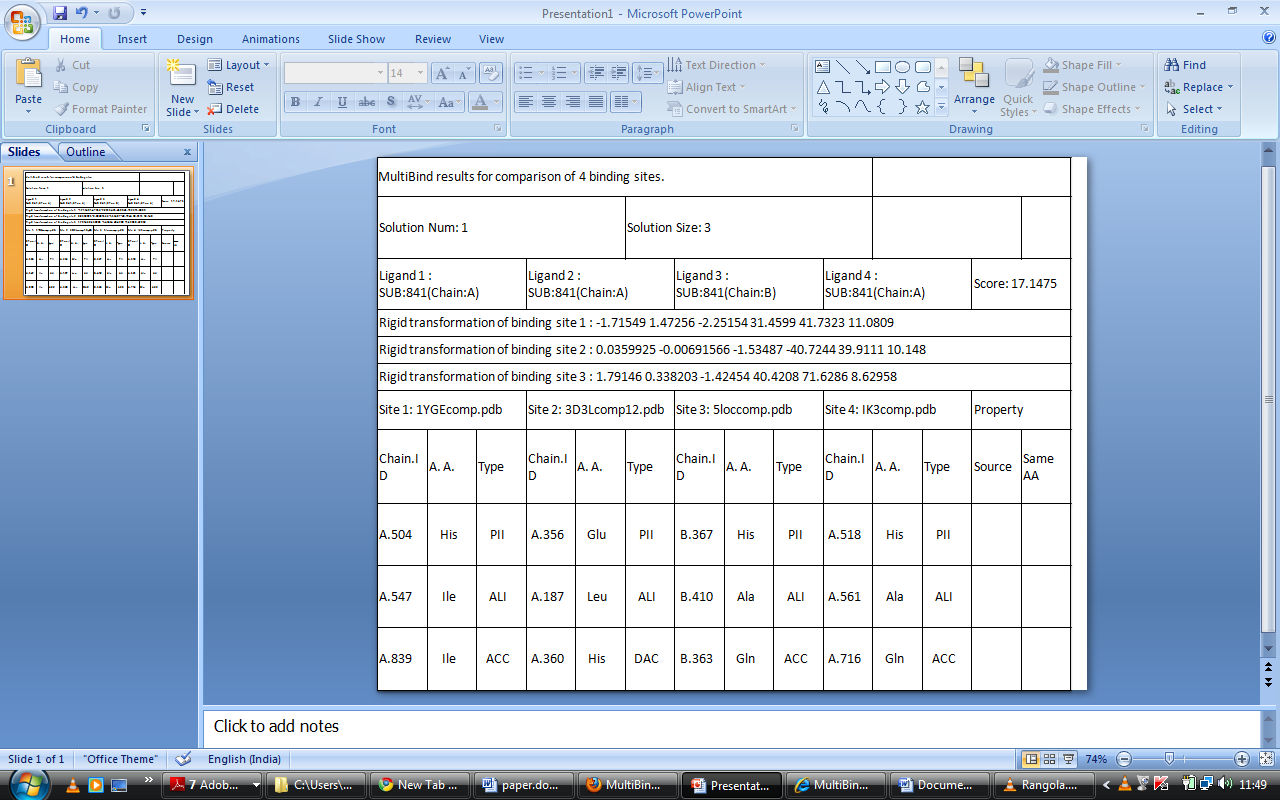


**24)sLOX1-12LOX-5LOX-COX1**


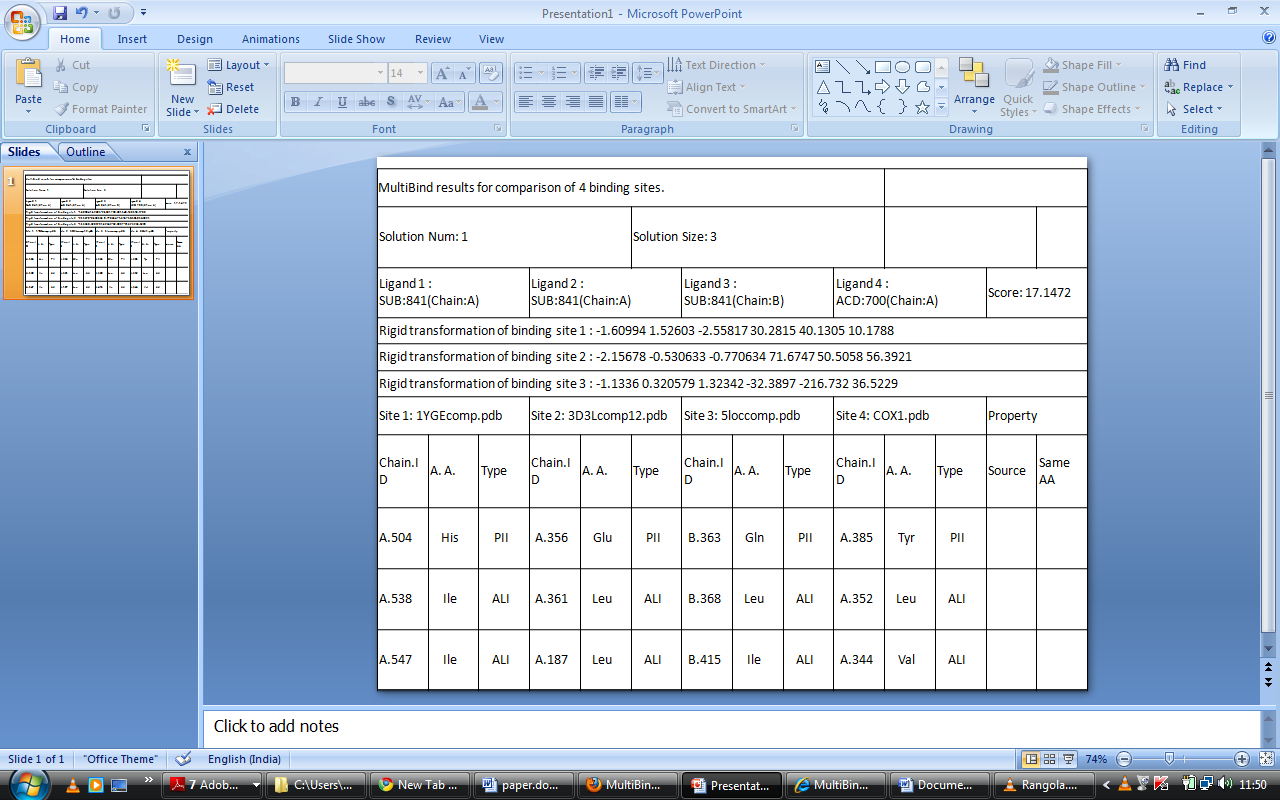


**25) sLOX1-12LOX-5LOX-COX2**


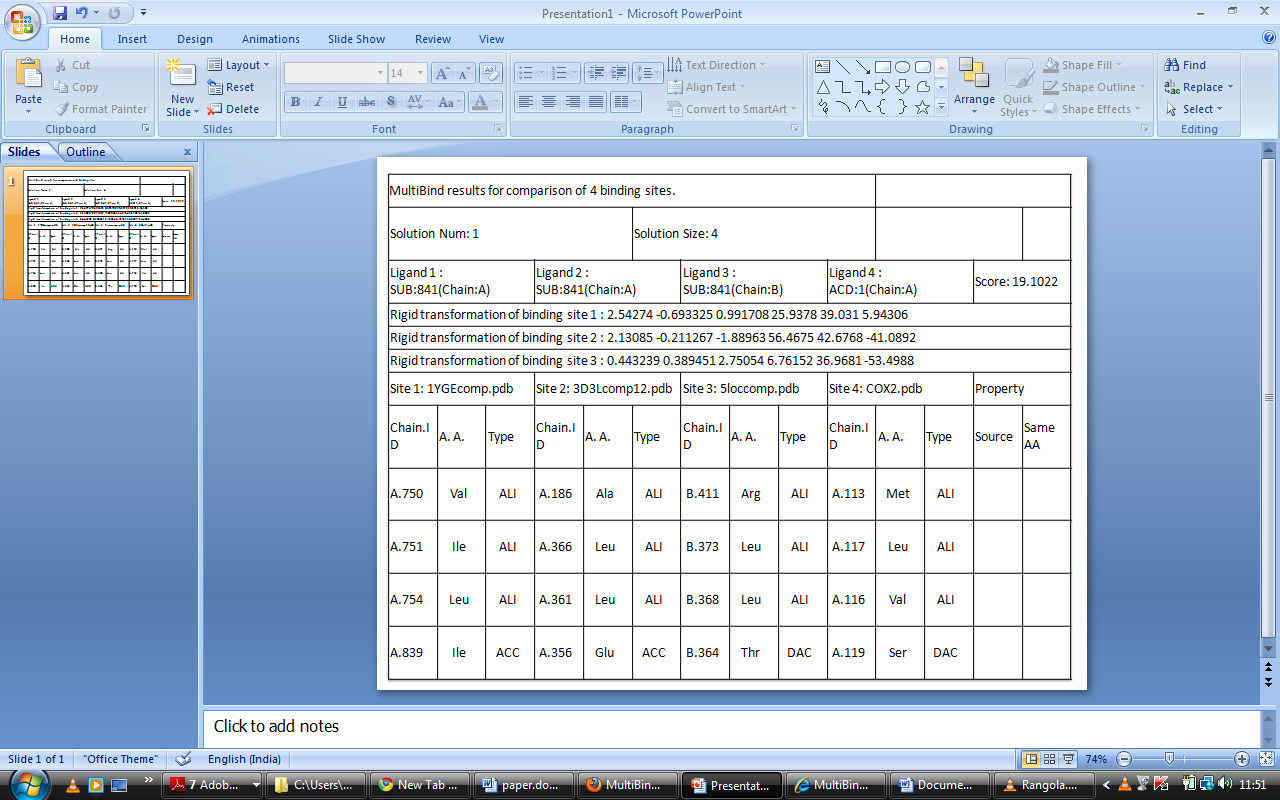


**26) sLOX1-12LOX-COX1-sLOX3**


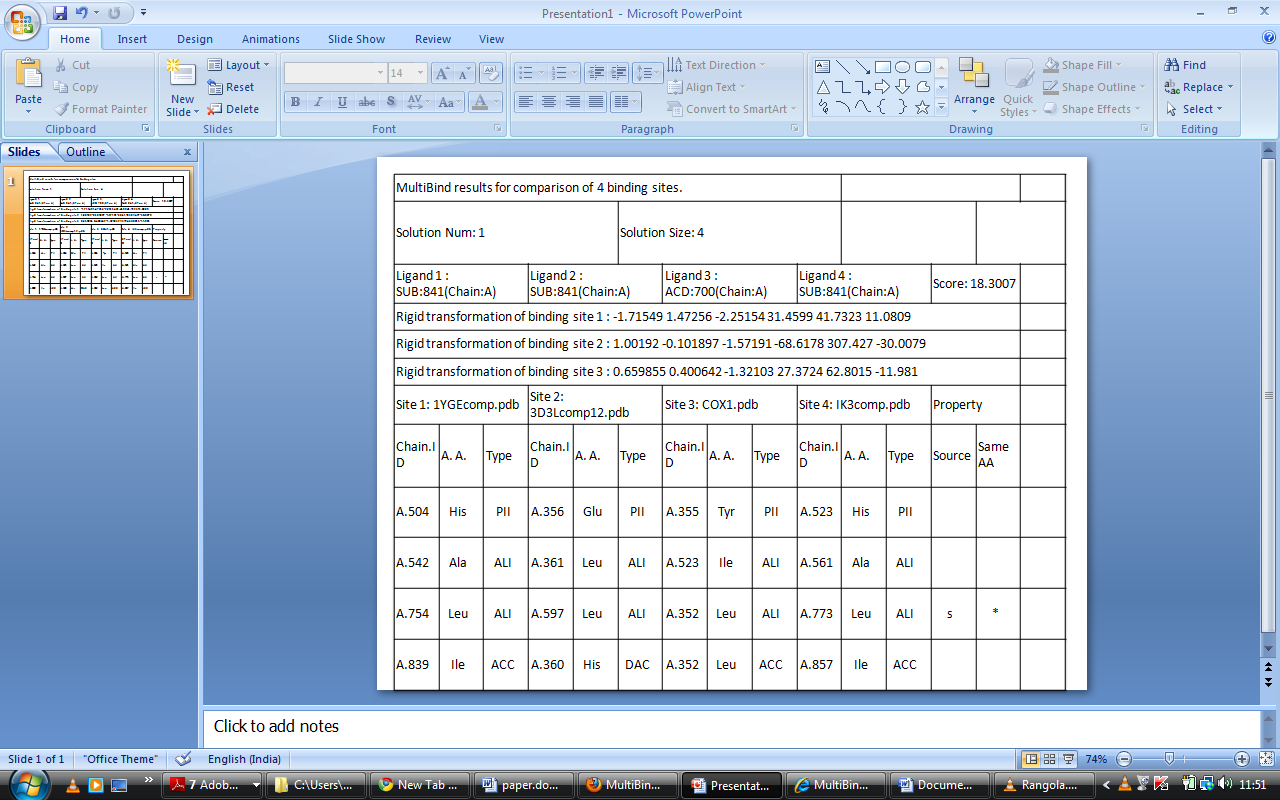


**27)sLOX1-12LOX-COX1-COX2**


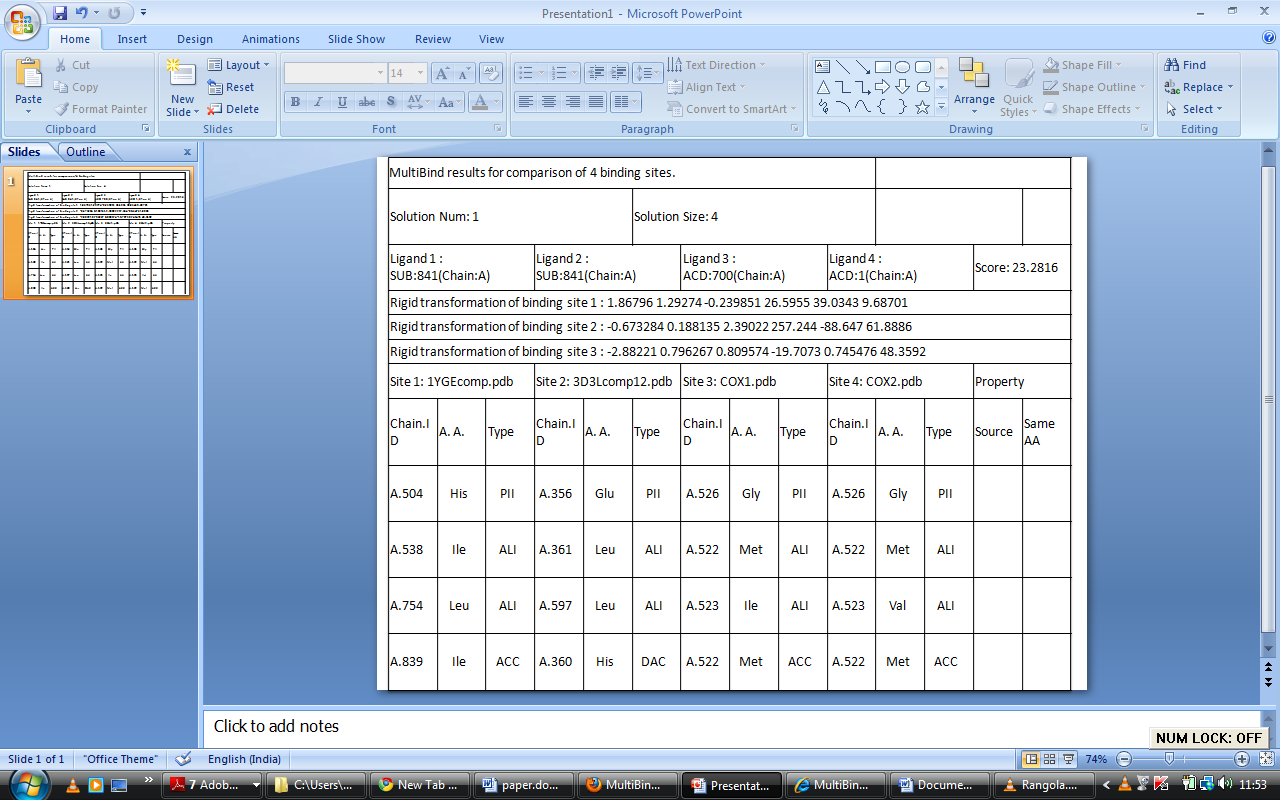


**28)sLOX1-12LOX-COX2-sLOX3**


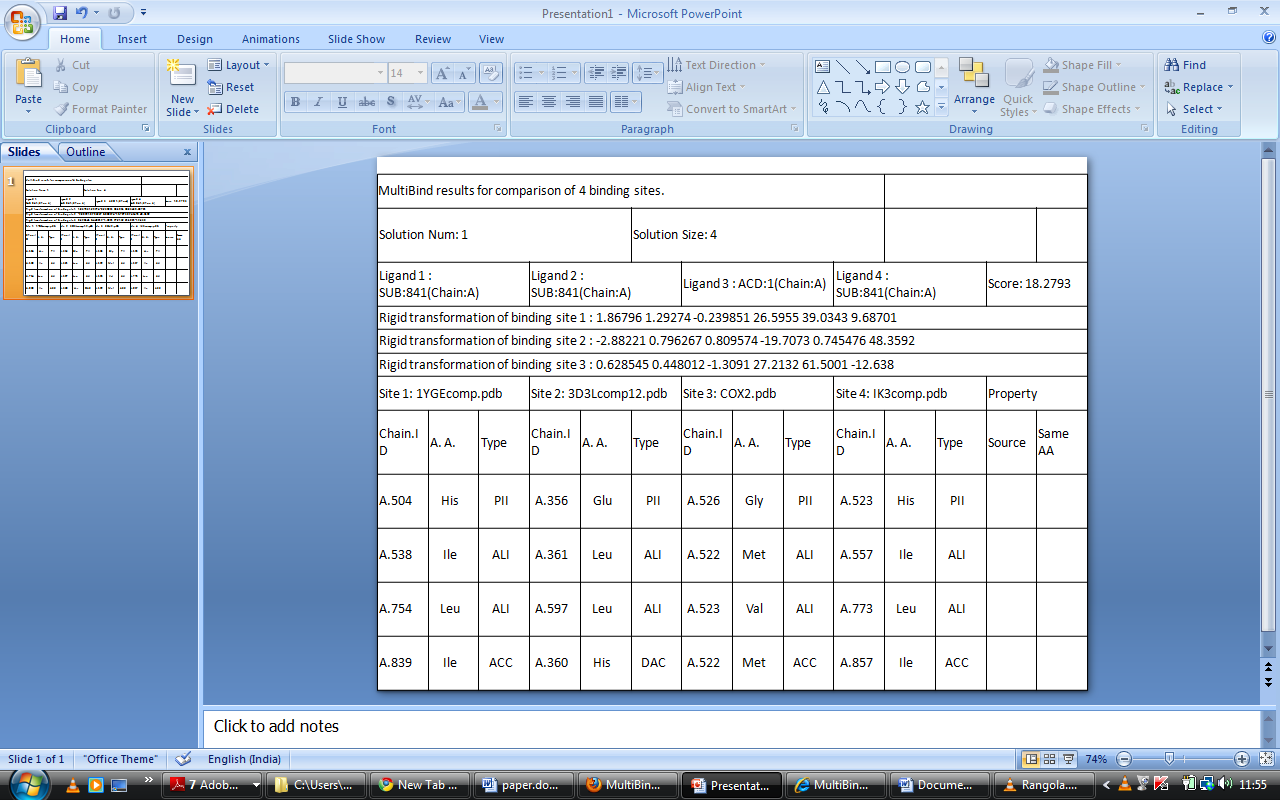


**29) sLOX1-5LOX-COX1-sLOX3**


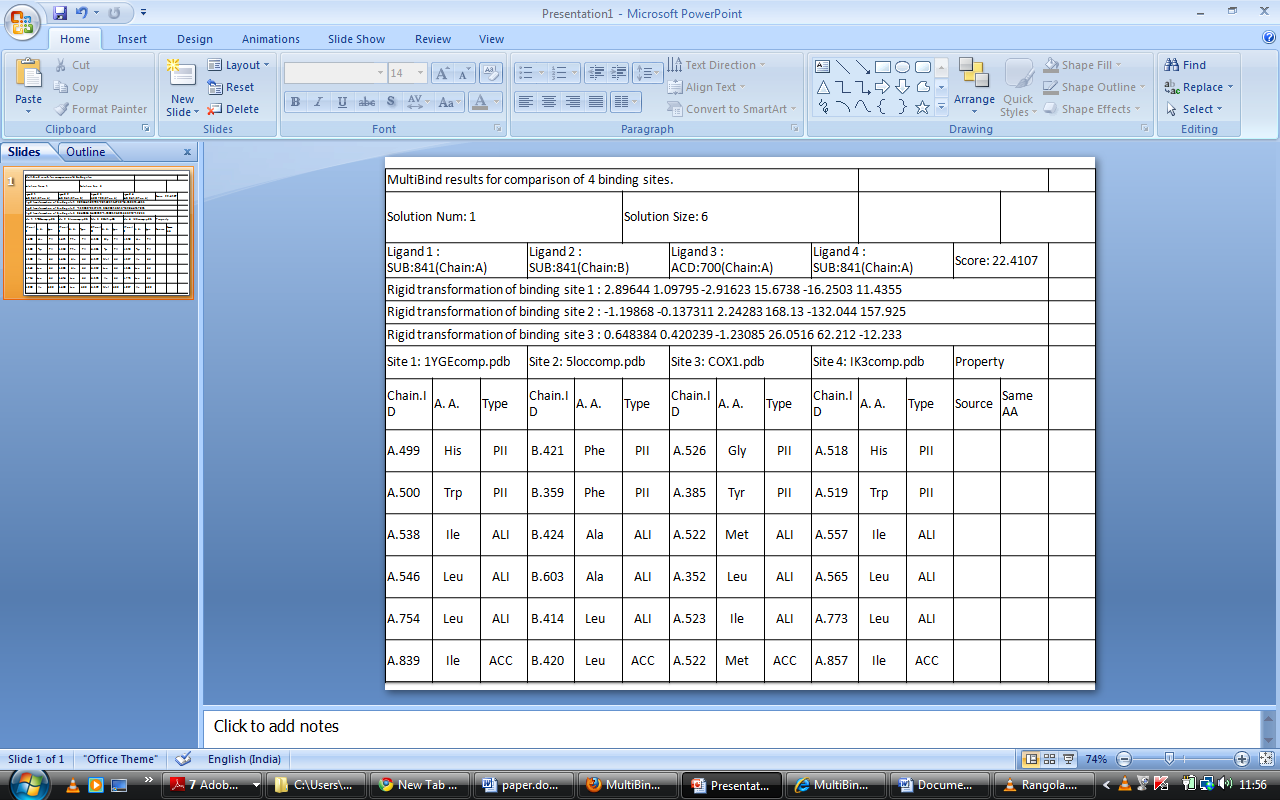


**30) sLOX1-5LOX-COX1-COX2**


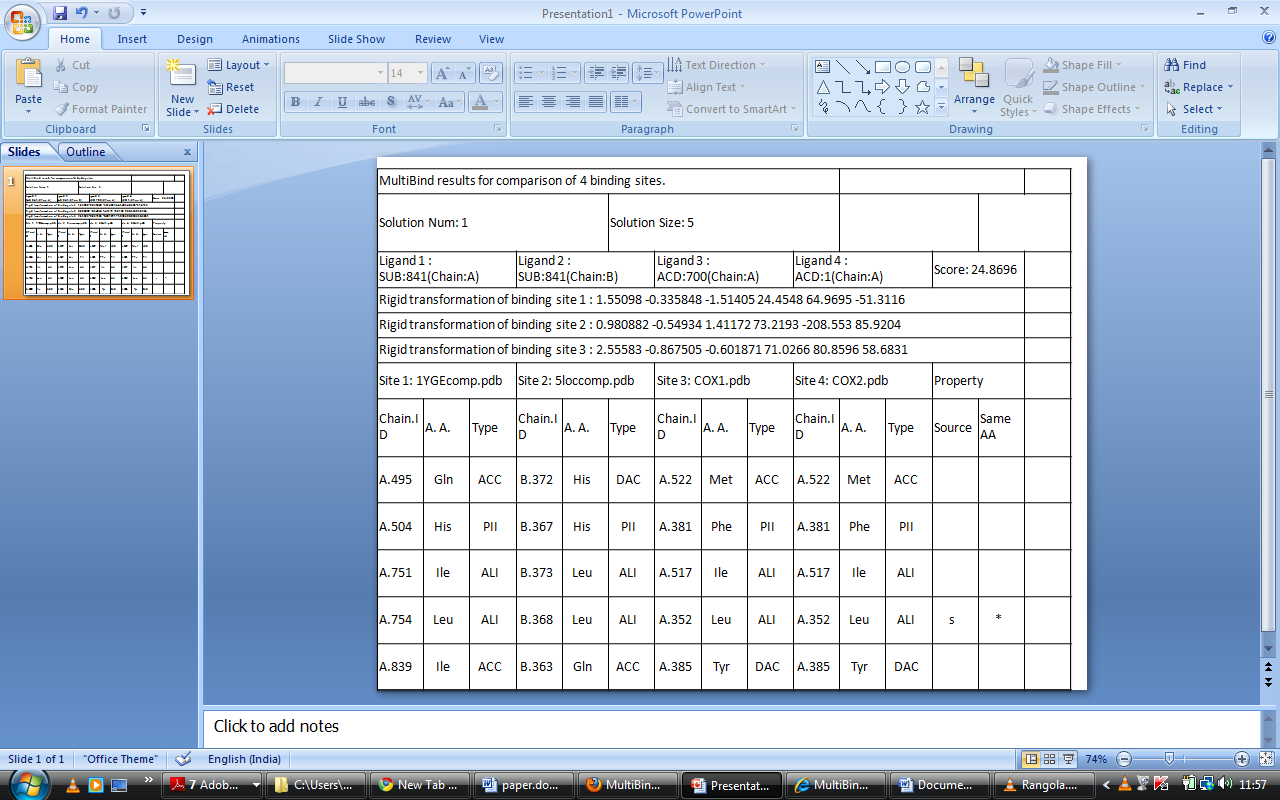


**31)sLOX1-5LOX-COX2-sLOX3**


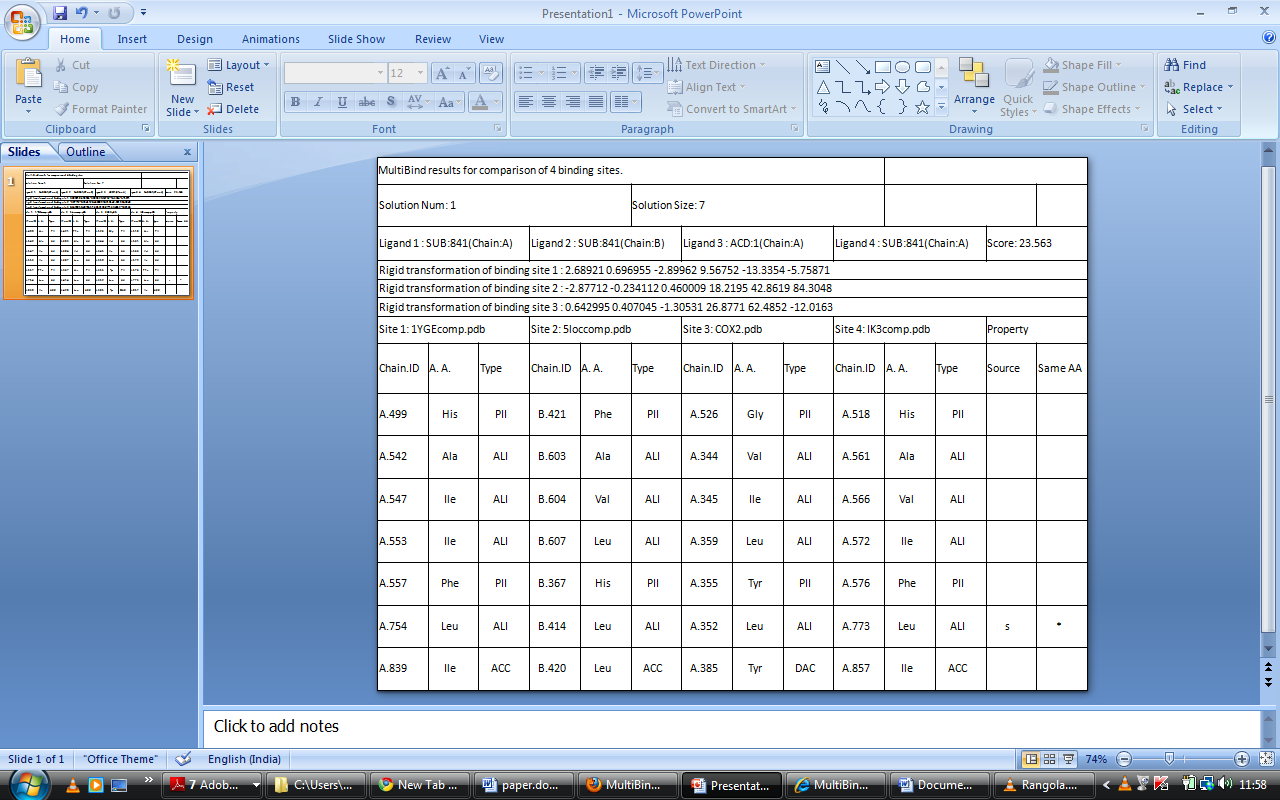


**32) 12LOX-5LOX-COX1-sLOX3**


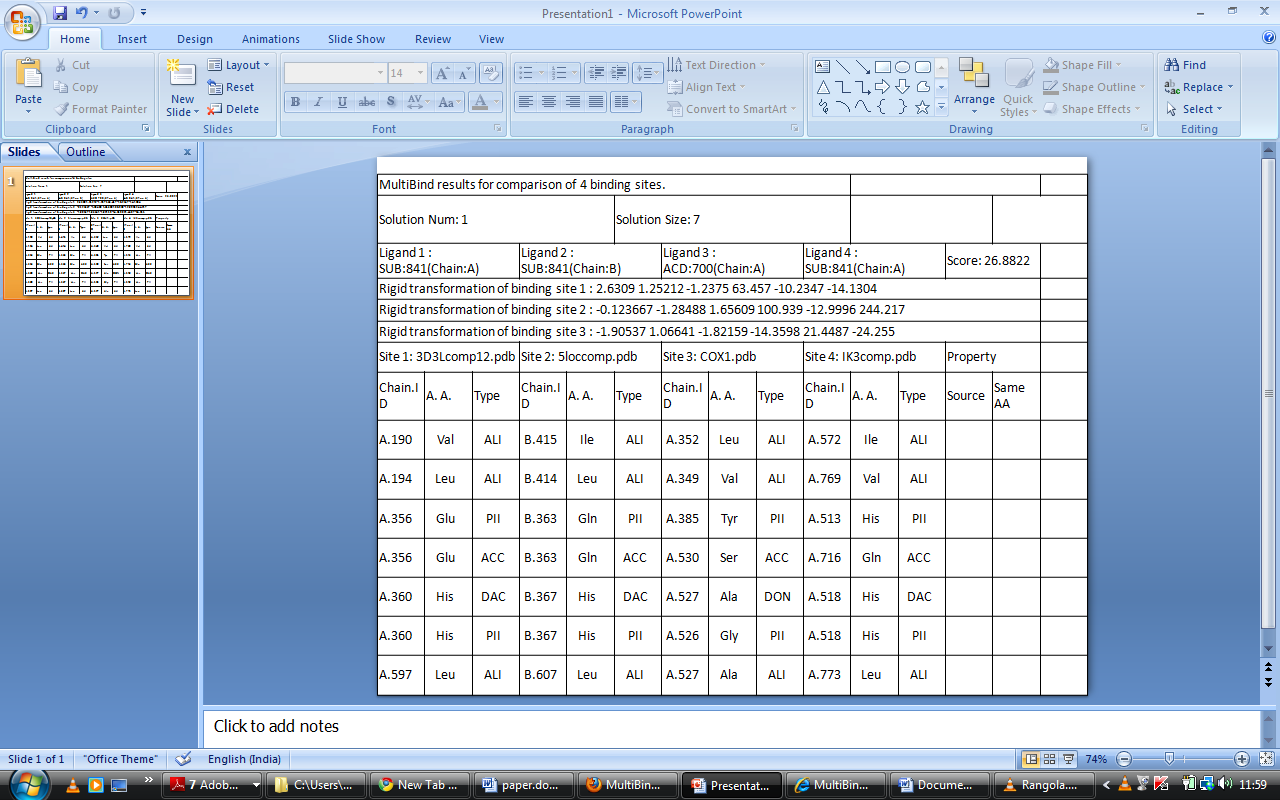


**33)12LOX-5LOX-COX2-sLOX3**


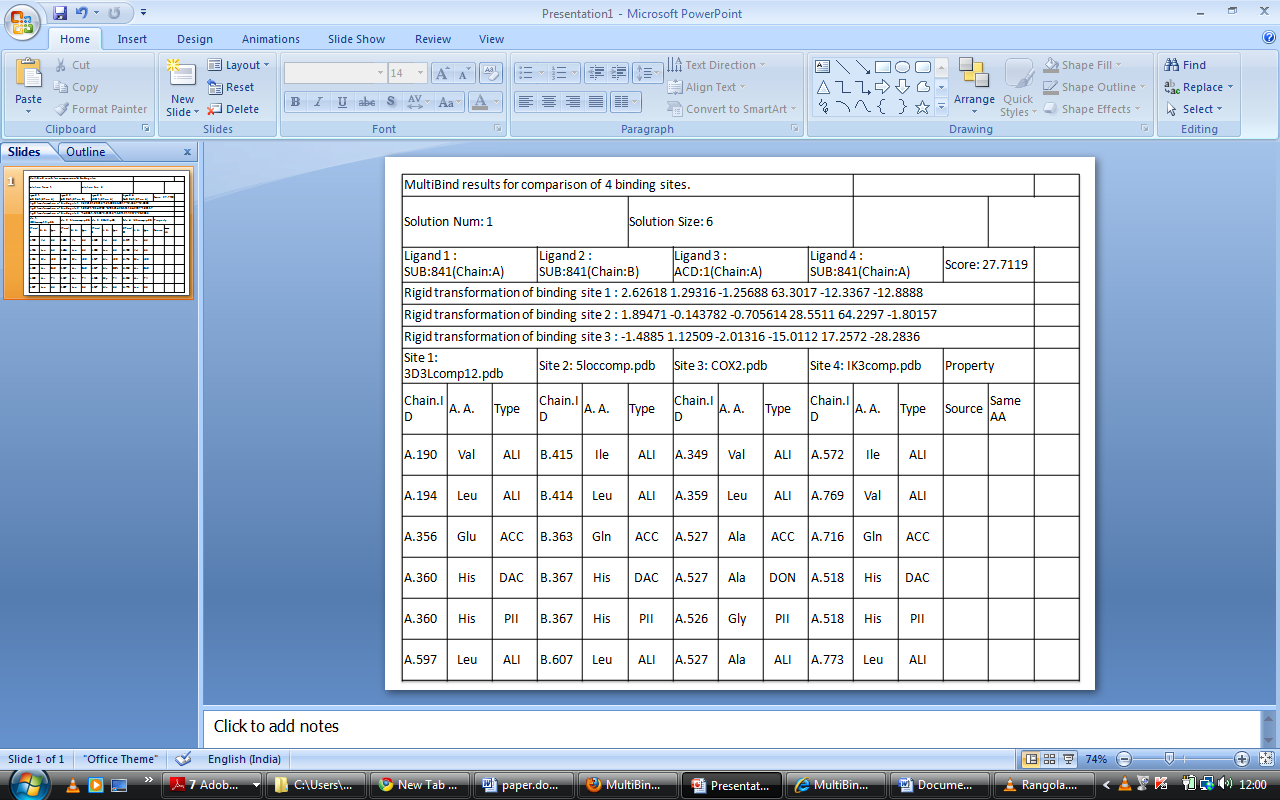


**34) 12LOX-COX1-COX2-sLOX3**


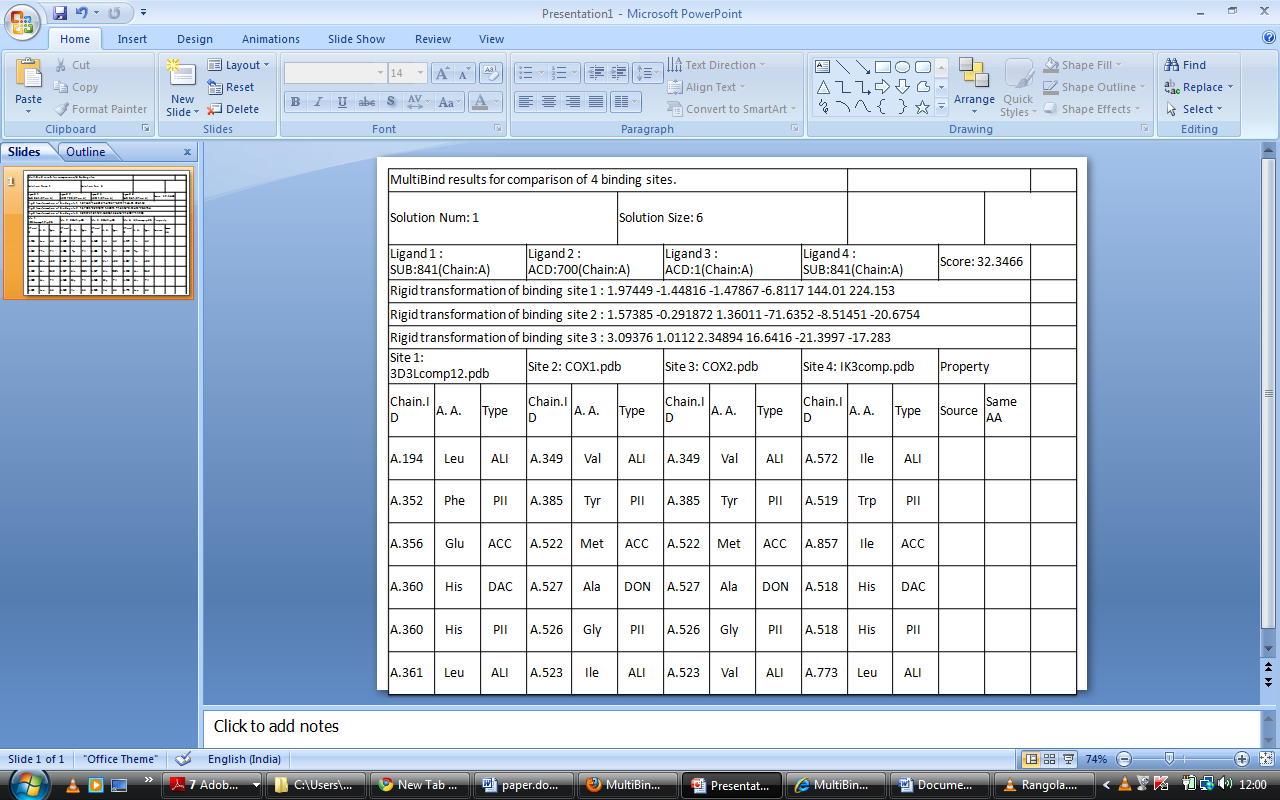


**35)12LOX-5LOX-COX1-COX2**


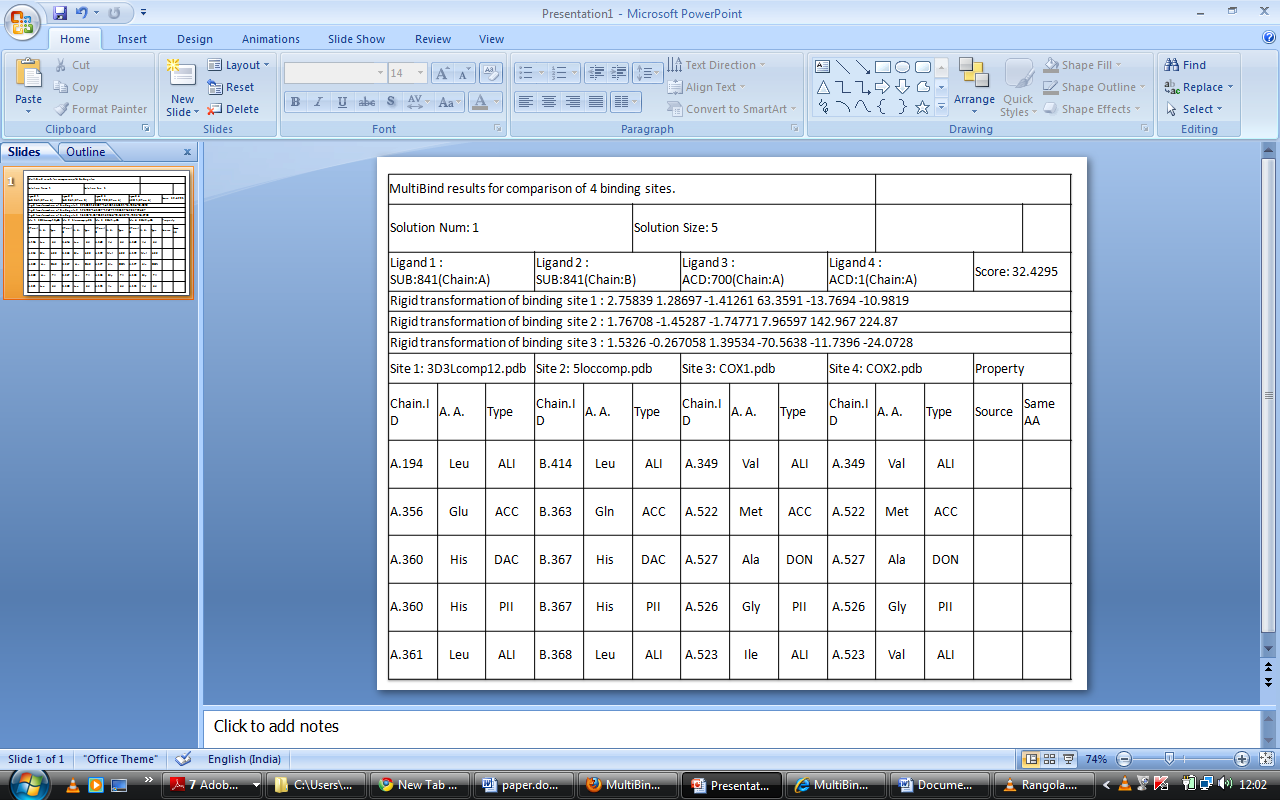


**36) 5LOX-COX1-COX2-sLOX3**


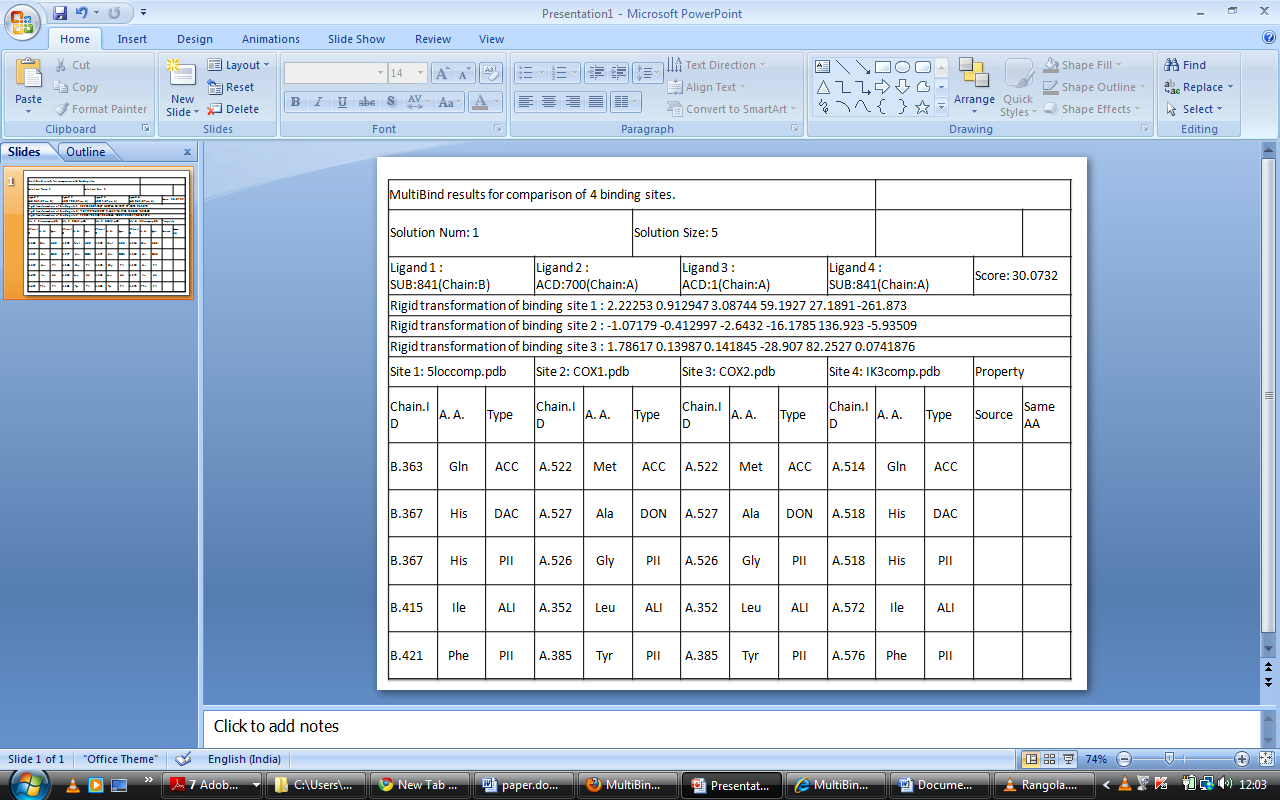


**MULTIPLE ALIGNMENTS OF FIVE BINDING SITES:**

The alignment between 5 binding sites resulted in 21 combinations. Comparison between 5 predicted binding sites of Arachidonic acid at a time revealed a pattern of between 6-3 common physiochemical properties. As the binding sites were aligned, eliminating one synthase at a time from the MULTIBIND during run time, the variation in the number of common physiochemical properties increased.

| **COMPARED PROTEINS** | **NO. OF DETECTED FEATURES** | **SCORE** |
| --- | --- | --- |
| 1. 15LOX-sLOX1-12LOX-COX1-sLOX3 | 5 | 20.7549 |
| 1. 15LOX-sLOX1-12LOX-5LOX-sLOX3 | 6 | 22.234 |
| 1. 15LOX-sLOX1-12LOX-5LOX-COX1 | 6 | 21.1242 |
| 1. 15LOX-sLOX1-12LOX-COX1-COX2 | 4 | 22.1076 |
| 1. 15LOX-sLOX1-5LOX-COX1-sLOX3 | 6 | 21.9424 |
| 1. 15LOX-sLOX1-5LOX-COX1-COX2 | 4 | 23.091 |
| 1. 15LOX-12LOX-5LOX-COX1-sLOX3 | 6 | 25.255 |
| 1. sLOX1-12LOX-5LOX-COX1-sLOX3 | 3 | 17.0781 |
| 1. sLOX1-5LOX-COX1-COX2-sLOX3 | 5 | 23.7522 |
| 1. 15LOX-sLOX1-12LOX-COX2-sLOX3 | 5 | 21.4176 |
| 1. 15LOX-sLOX1-5LOX-COX2-sLOX3 | 4 | 21.257 |
| 1. 15LOX-sLOX1-COX1-COX2-sLOX3 | 4 | 22.7136 |
| 1. 15LOX-12LOX-COX1-COX2-sLOX3 | 5 | 28.0485 |
| 1. 15LOX-5LOX-COX1-COX2-sLOX3 | 5 | 29.7177 |
| 1. sLOX1-12LOX-5LOX-COX2-sLOX3 | 3 | 17.5138 |
| 1. sLOX1-12LOX-COX1-COX2-sLOX3 | 4 | 22.8794 |
| 1. 15LOX-12LOX-5LOX-COX2-sLOX3 | 5 | 26.8689 |
| 1. 12LOX-5LOX-COX1-COX2-sLOX3 | 5 | 30.3481 |
| 1. 15LOX-sLOX1-12LOX-5LOX-COX2 | 4 | 21.53336 |
| 20)15LOX-12LOX-5LOX-COX1-COX2 | 5 | 28.8917 |
| 21)sLOX1-12LOX-5LOX-COX1-COX2 | 4 | 19.7853 |

**1) 15LOX-sLOX1-12LOX-COX1-sLOX3**


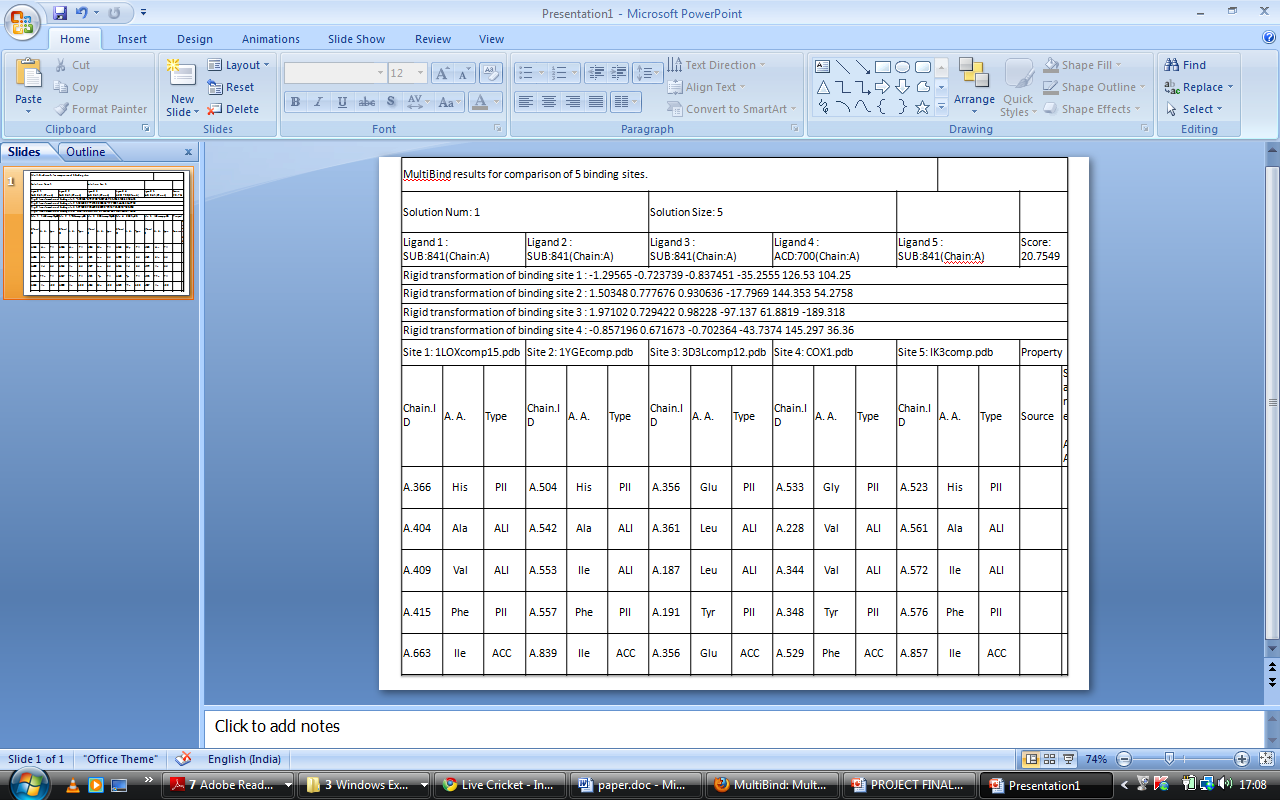


**2)15LOX-sLOX1-12LOX-5LOX-sLOX3**


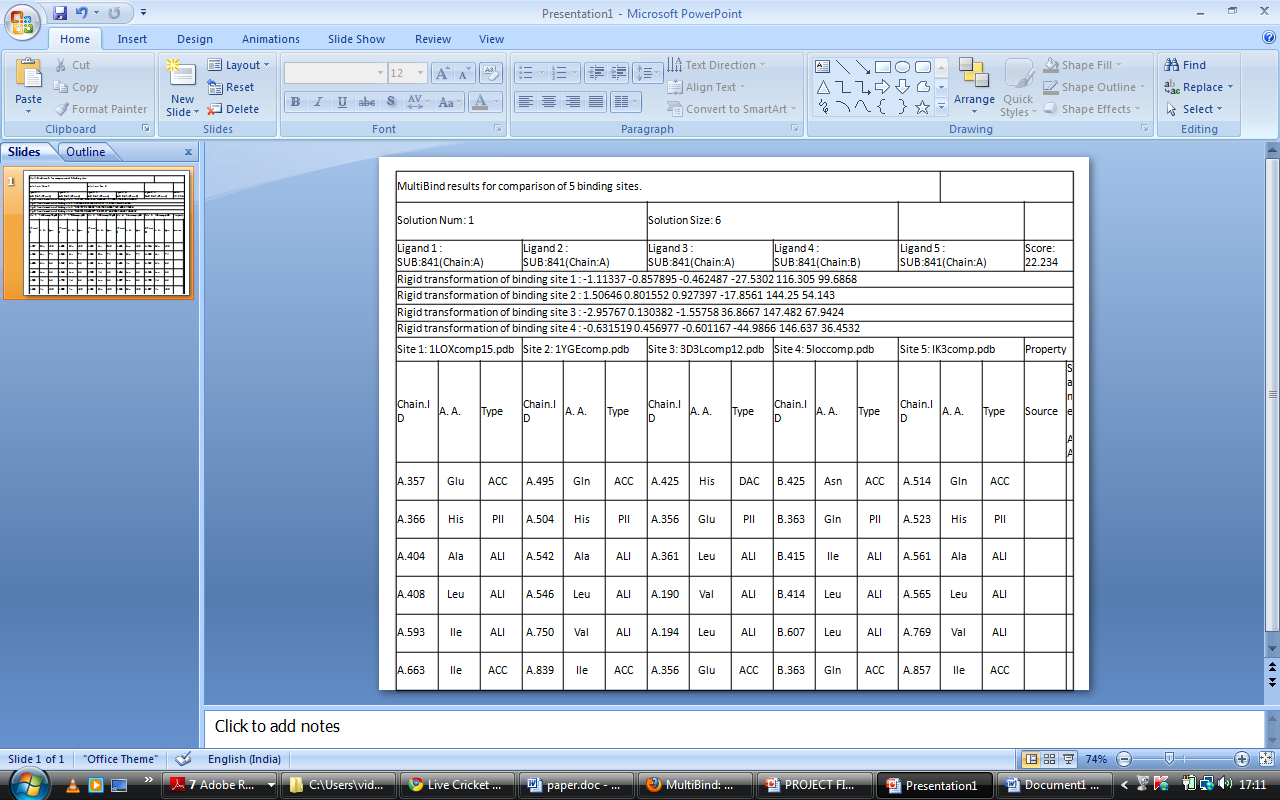


**3)15LOX-sLOX1-12LOX-5LOX-COX1**


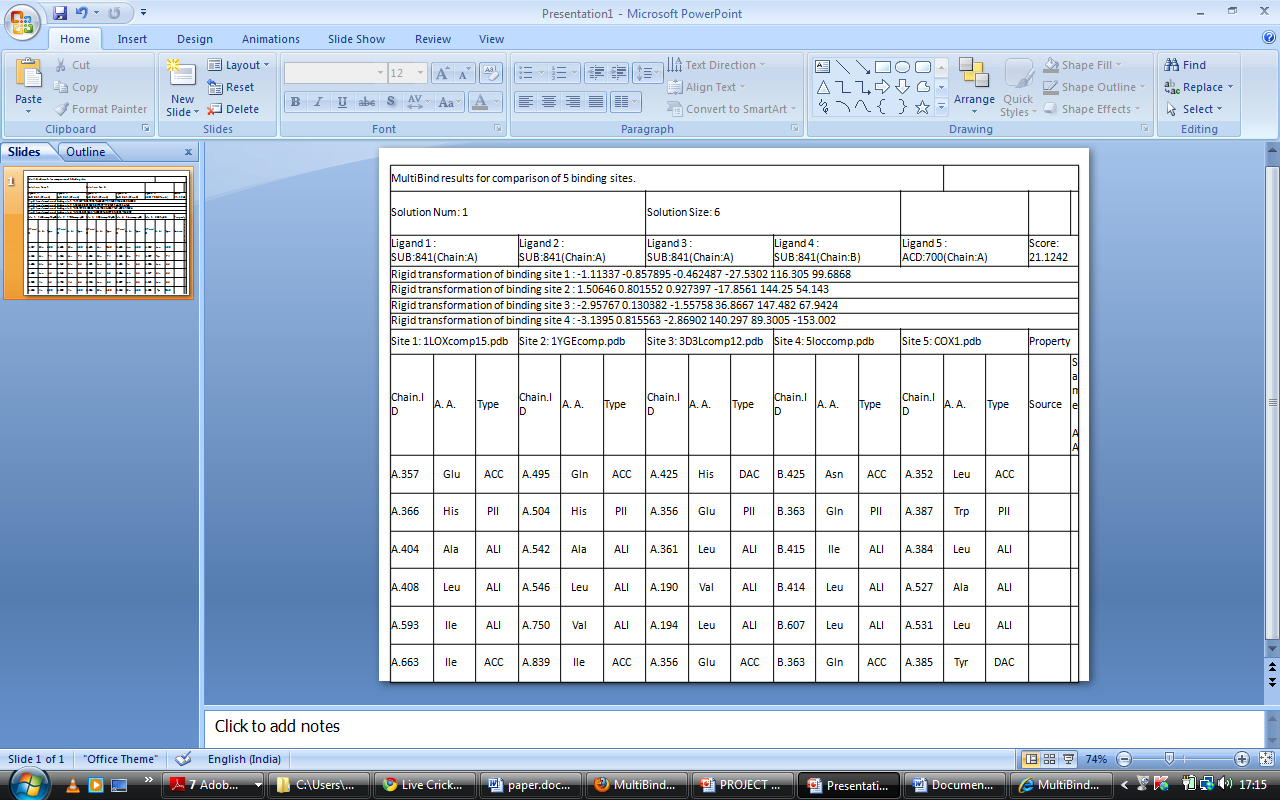


**4)15LOX-sLOX1-12LOX-COX1-COX2**


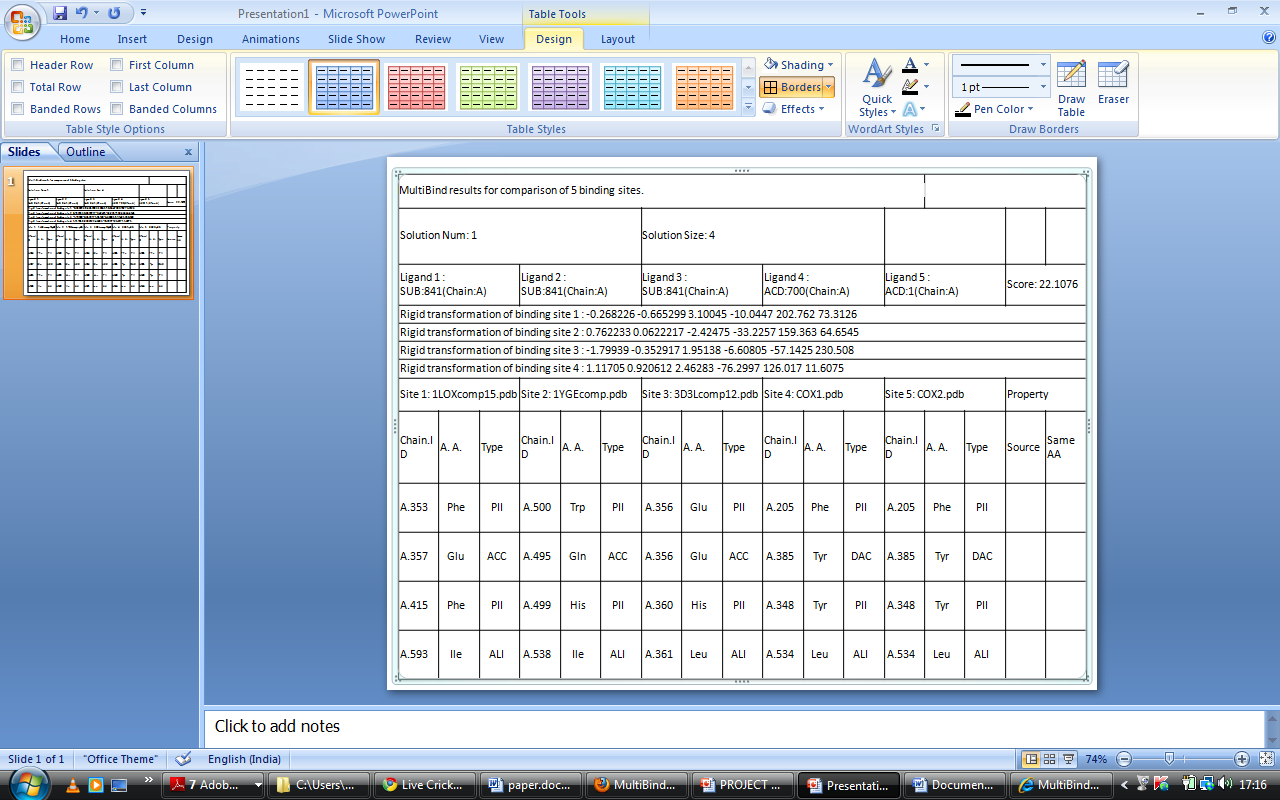


**5)15LOX-sLOX1-5LOX-COX1-sLOX3**


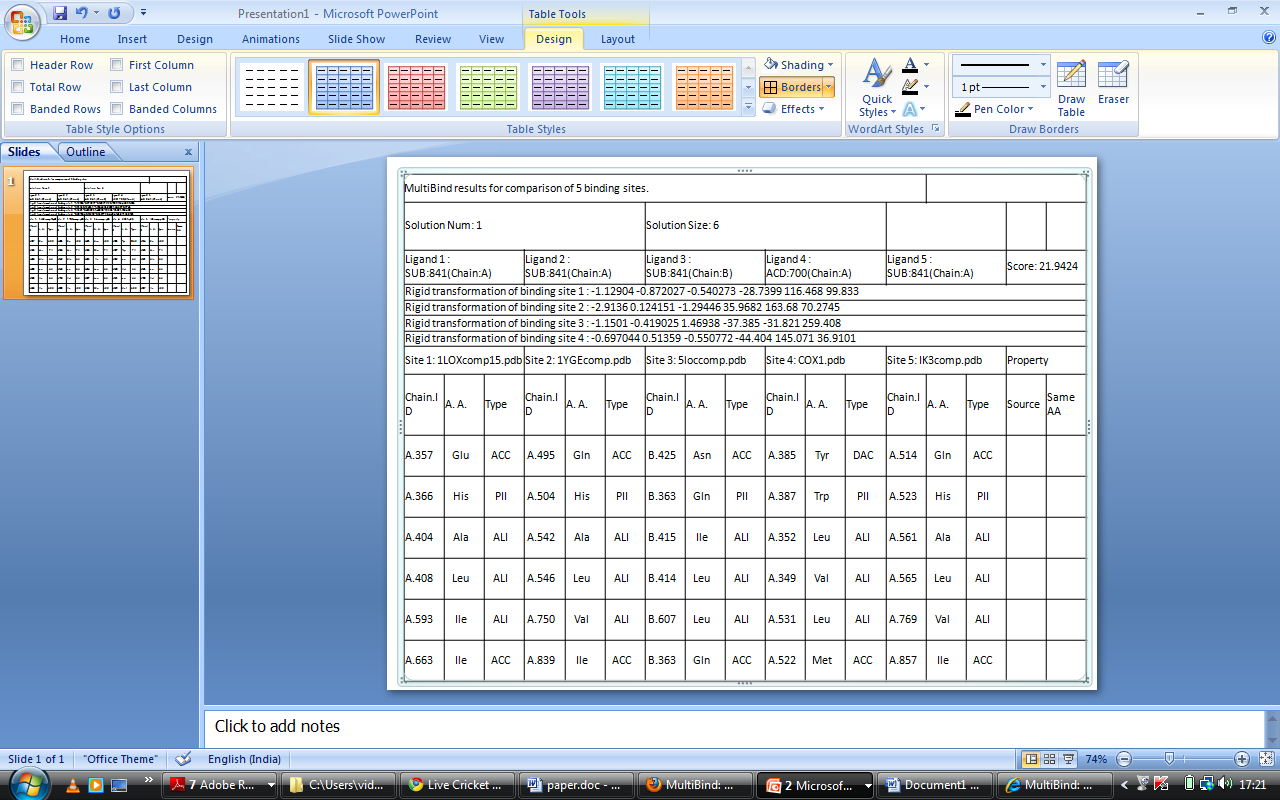


**6) 15LOX-sLOX1-5LOX-COX1-COX2**


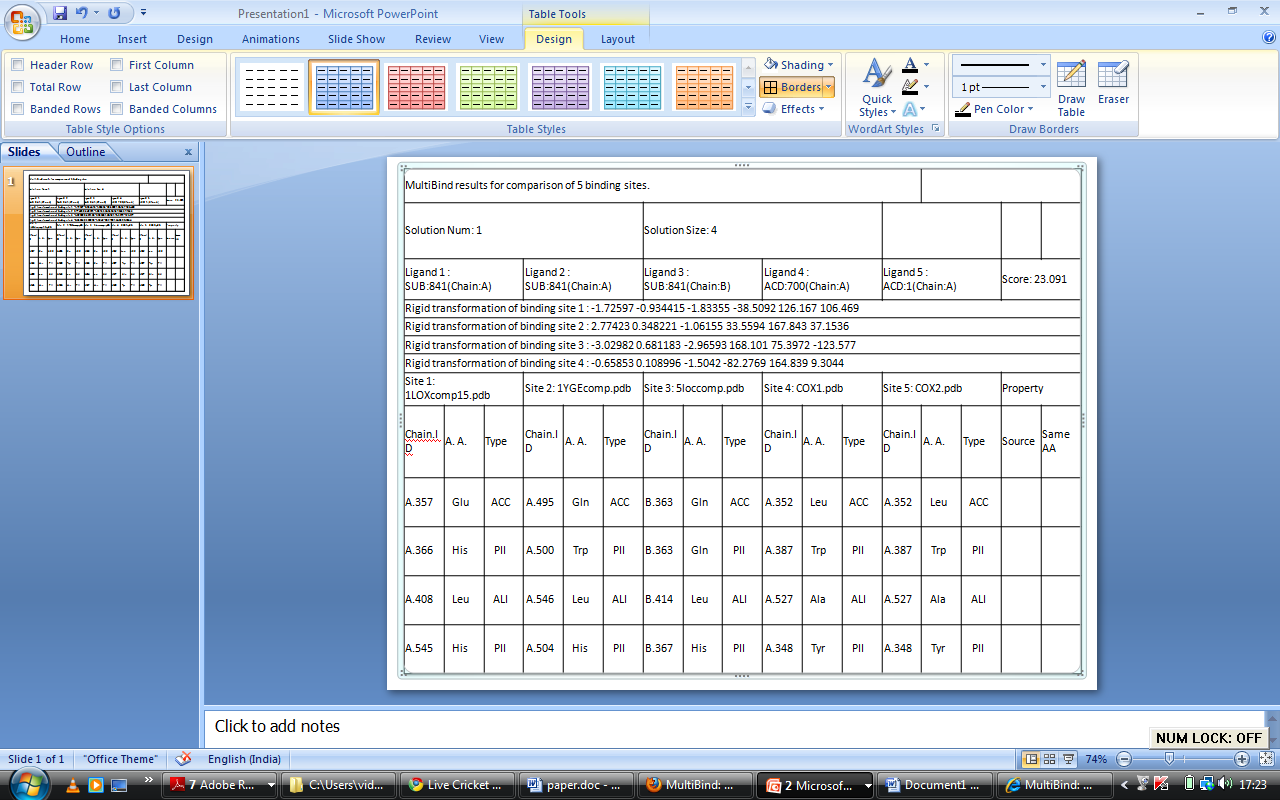


**7) 15LOX-12LOX-5LOX-COX1-sLOX3**


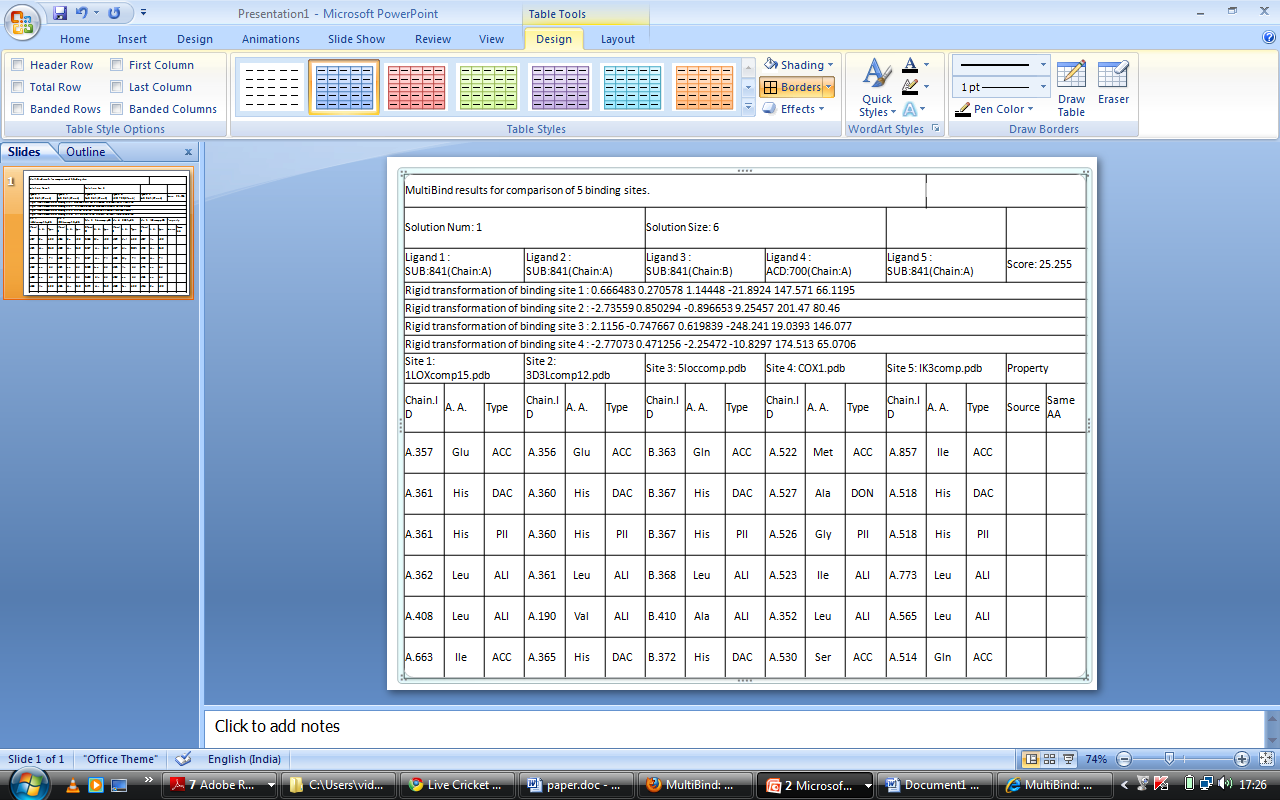


**8)sLOX1-12LOX-5LOX-COX1-sLOX3**


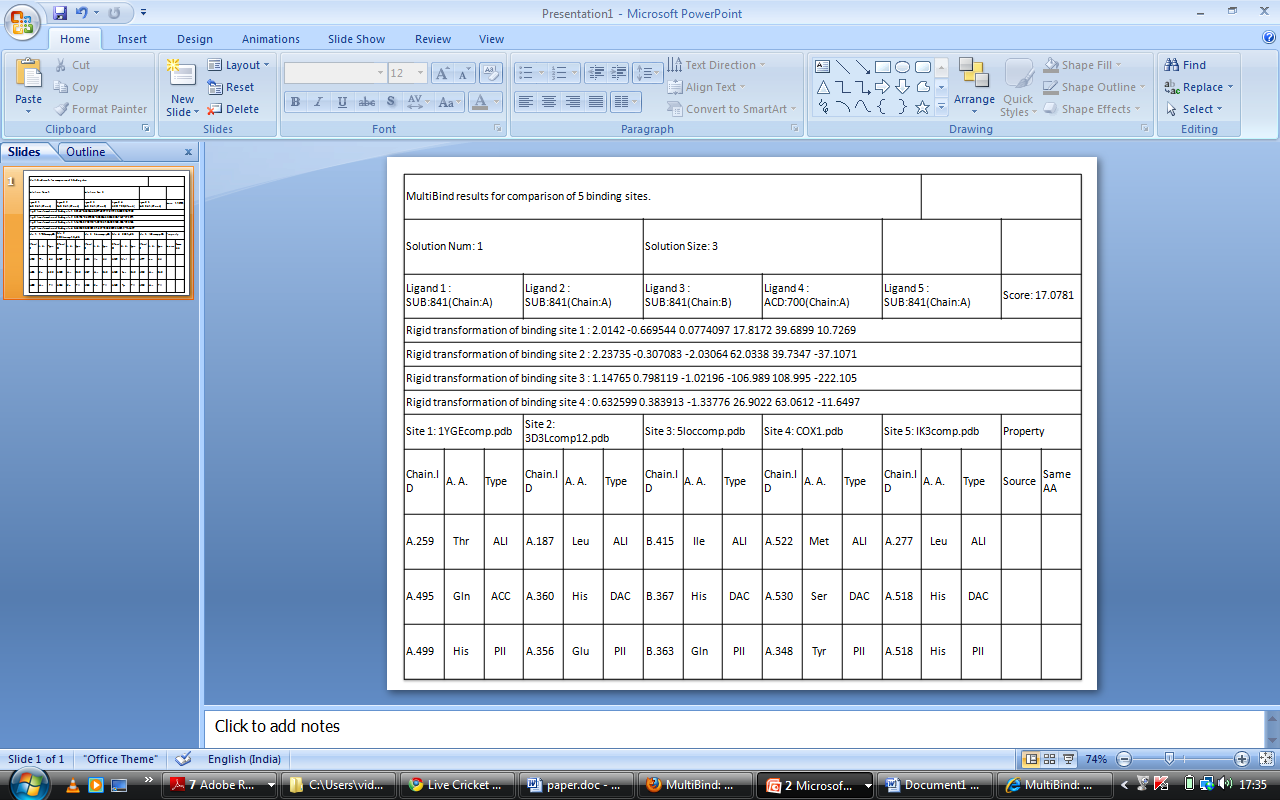


**9)sLOX1-5LOX-COX1-COX2-sLOX3**


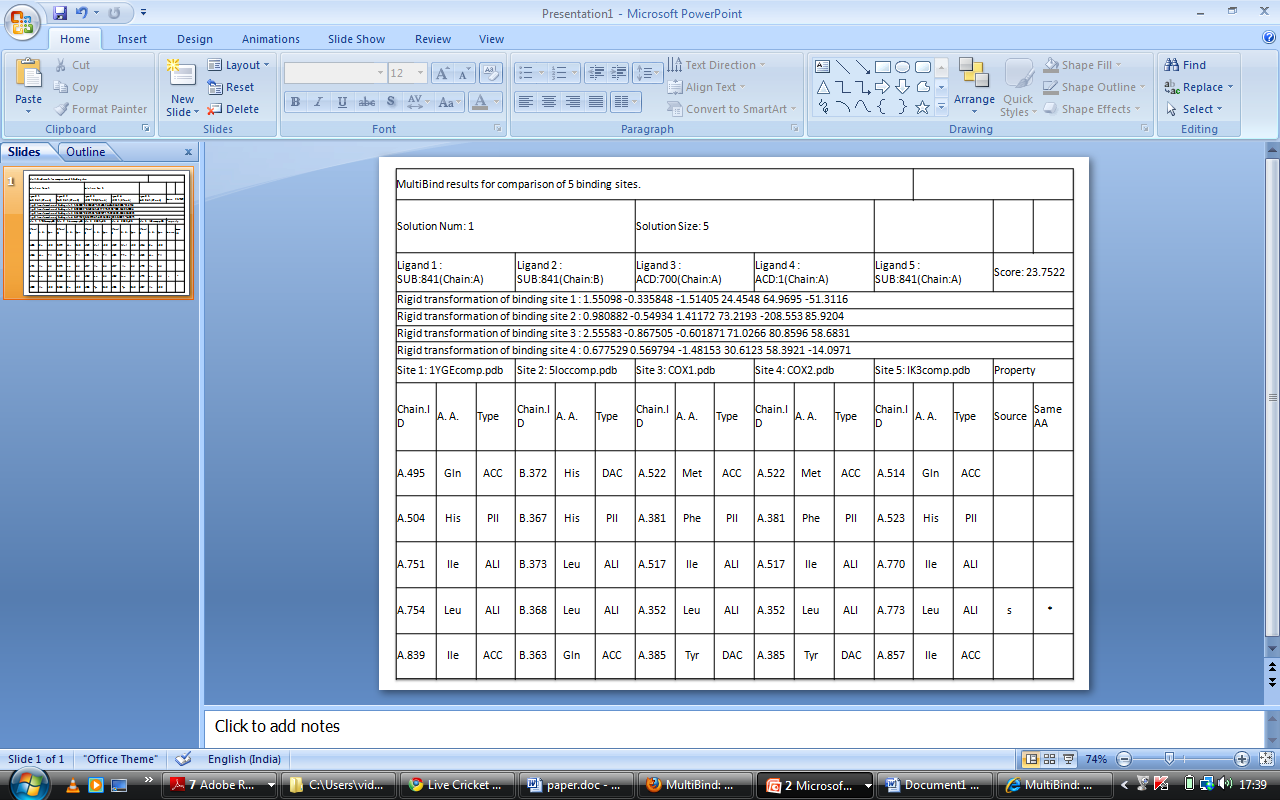


**10) 15LOX-sLOX1-12LOX-COX2-sLOX3**


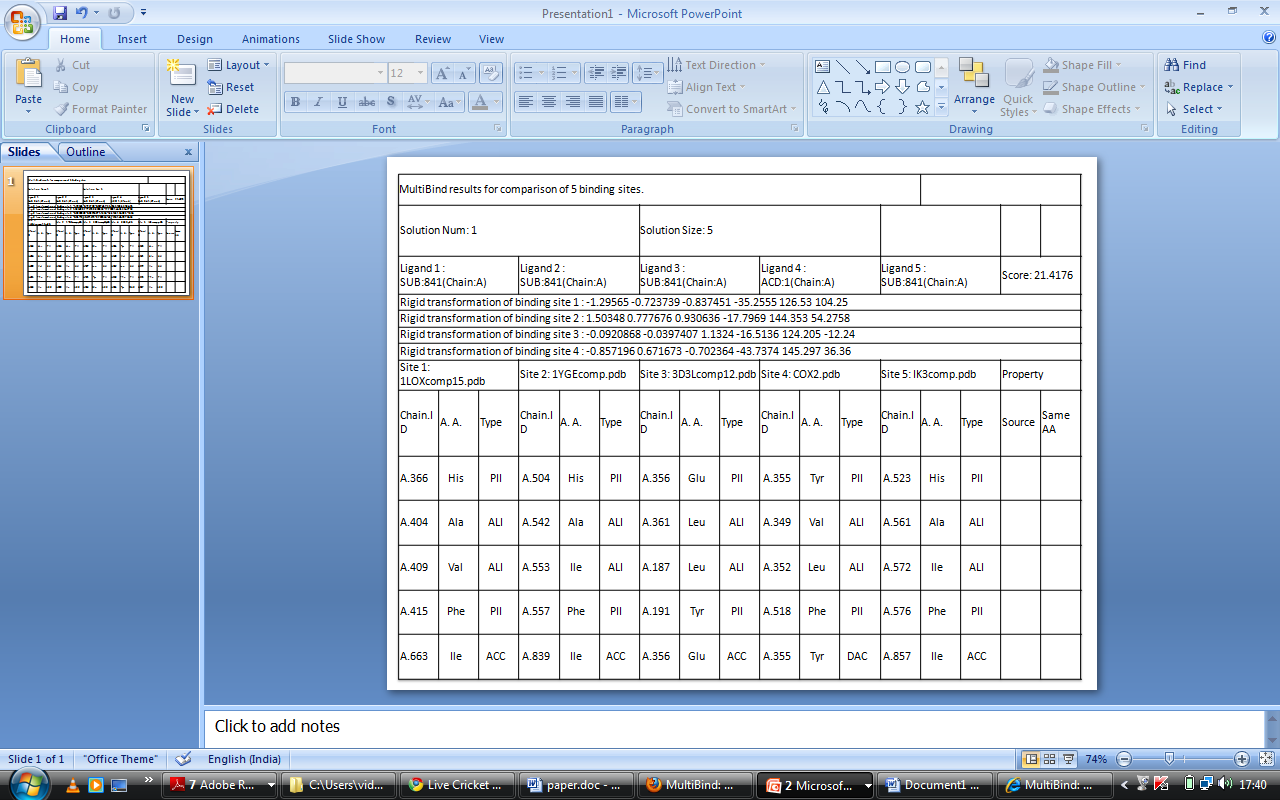


**11) 15LOX-sLOX1-5LOX-COX2-sLOX3**


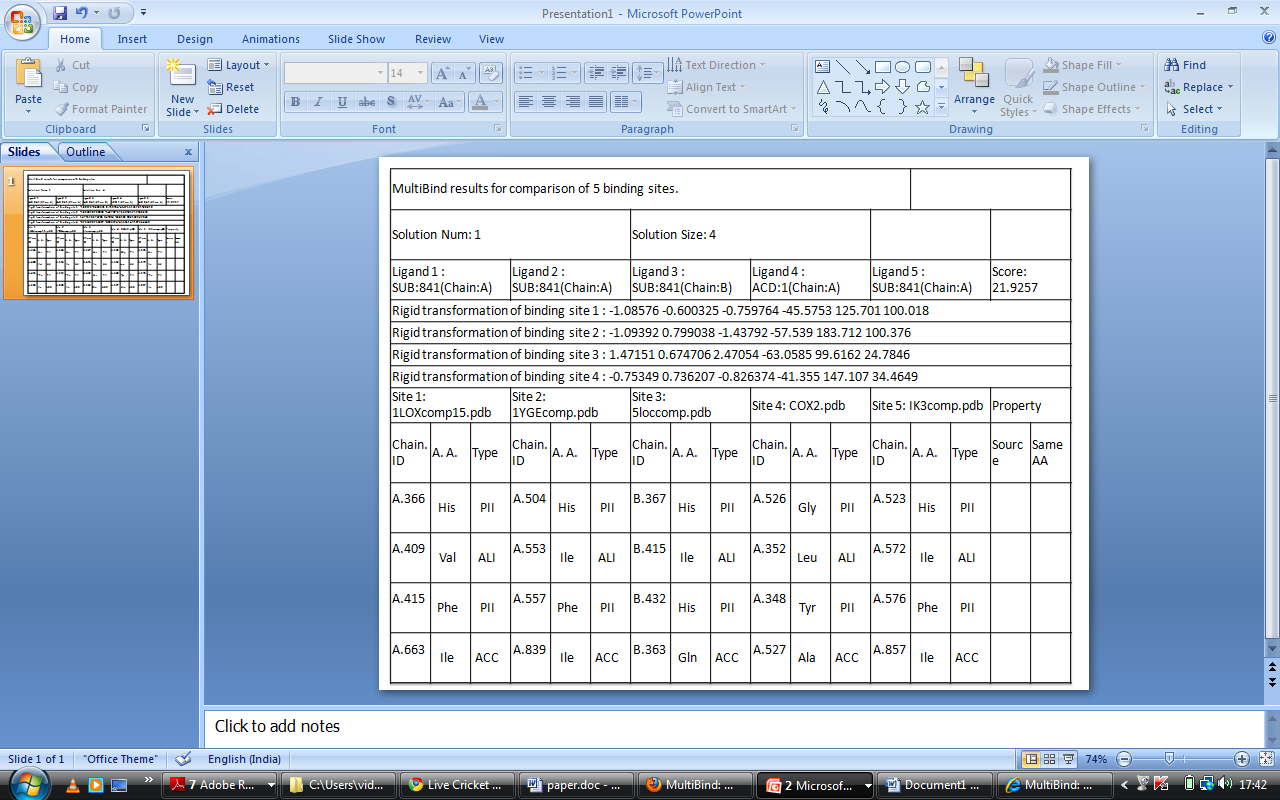


**12) 15LOX-sLOX1-COX1-COX2-sLOX3**


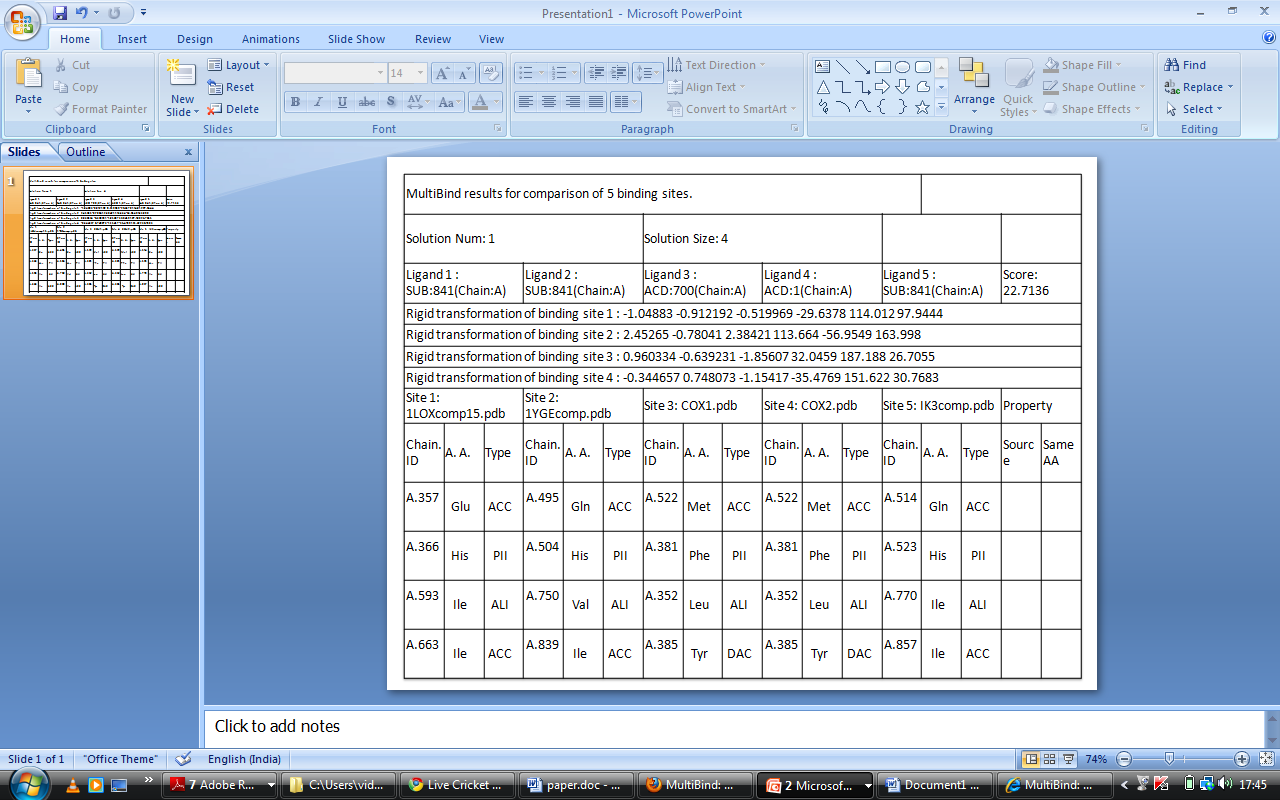


**13) 15LOX-12LOX-COX1-COX2-sLOX3**


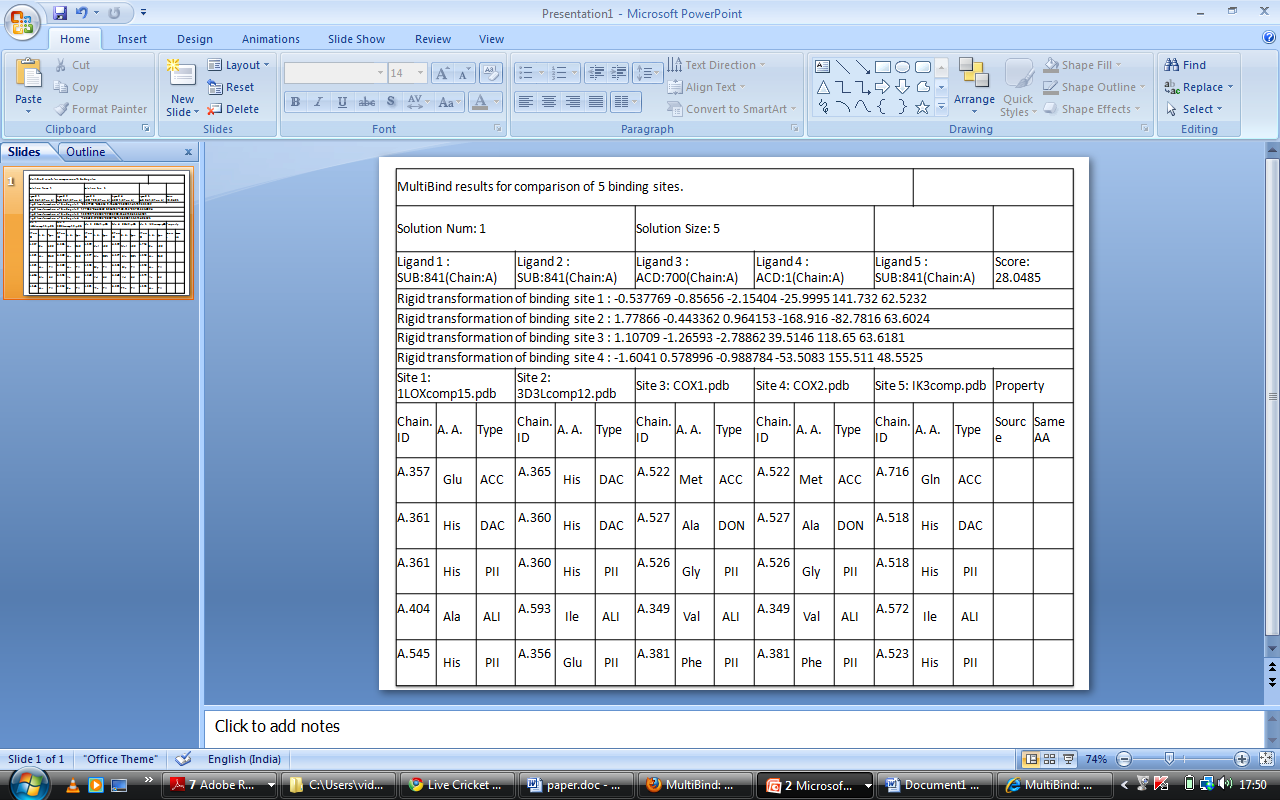


**14) 15LOX-5LOX-COX1-COX2-sLOX3**


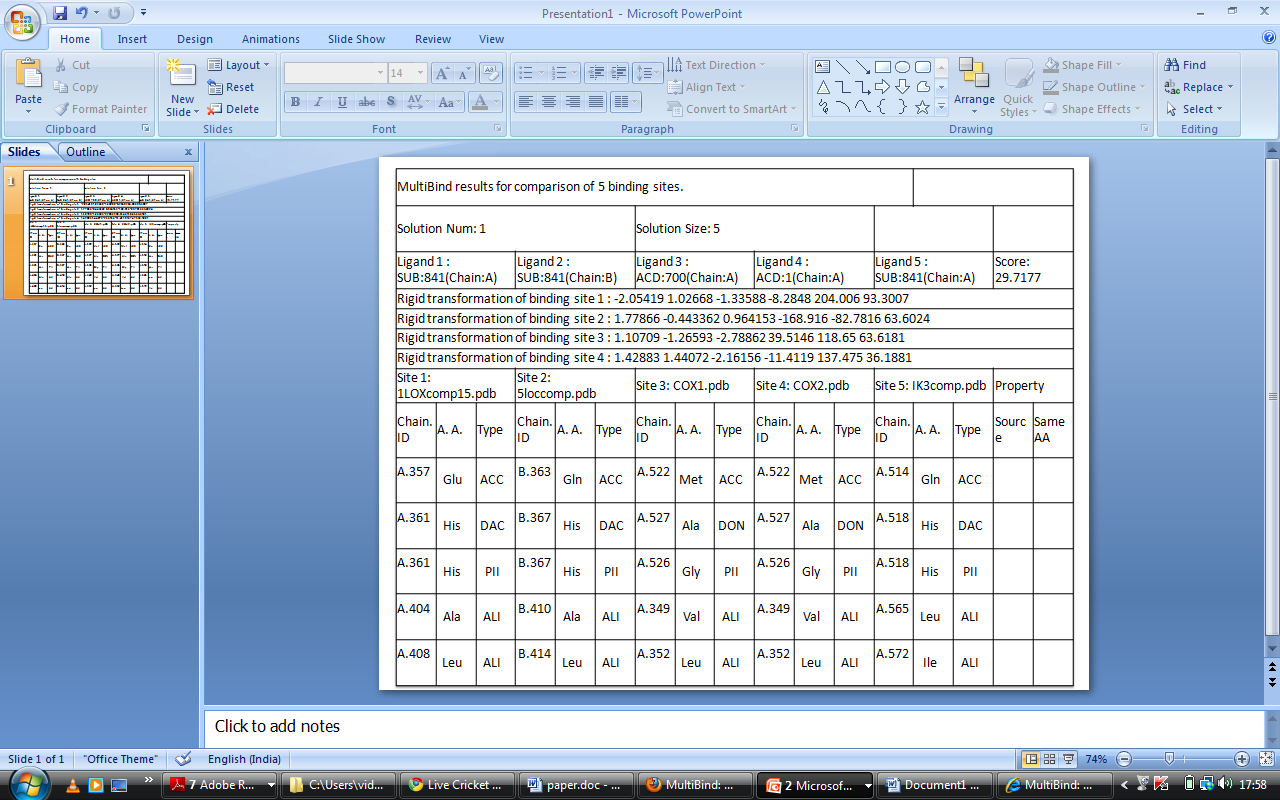


**15) sLOX1-12LOX-5LOX-COX2-sLOX3**


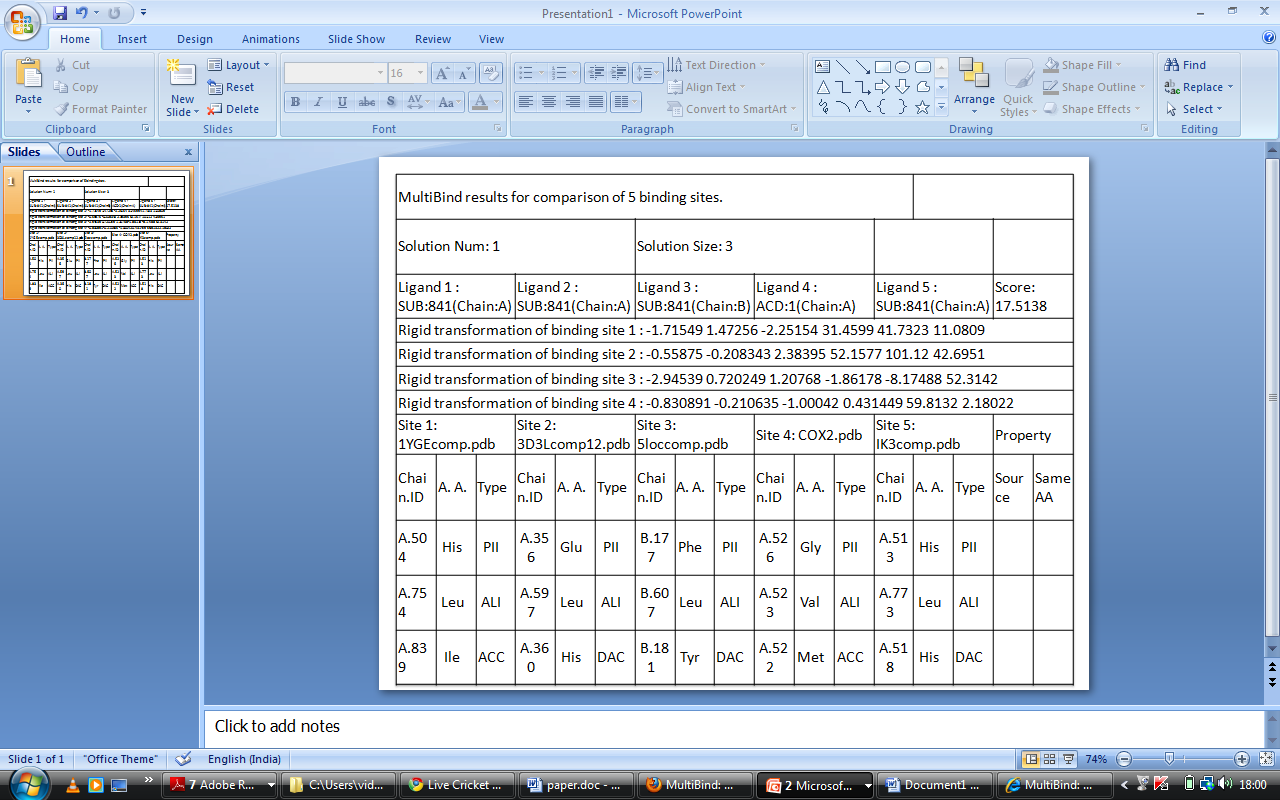


**16)** **sLOX1-12LOX-COX1-COX2-sLOX3**


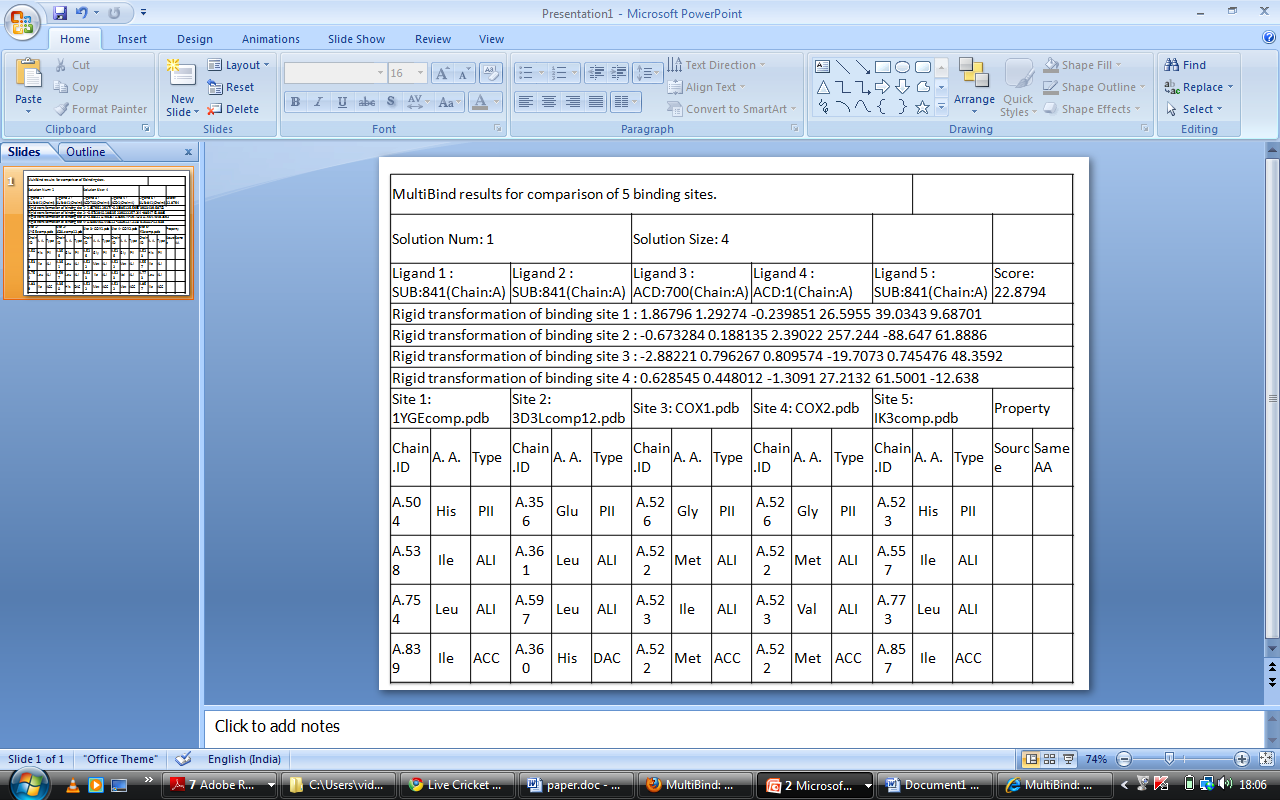


**17) 15LOX-12LOX-5LOX-COX2-sLOX3**


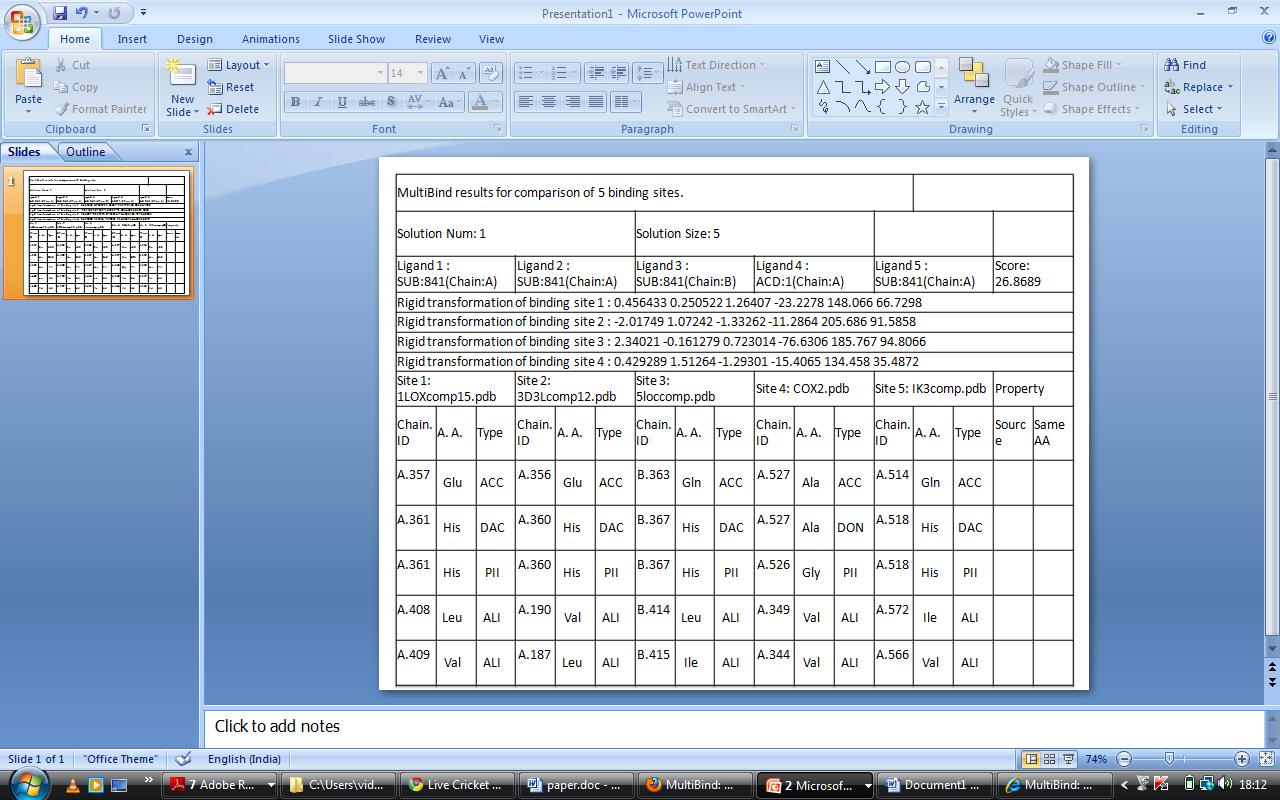


**18)12LOX-5LOX-COX1-COX2-sLOX3**


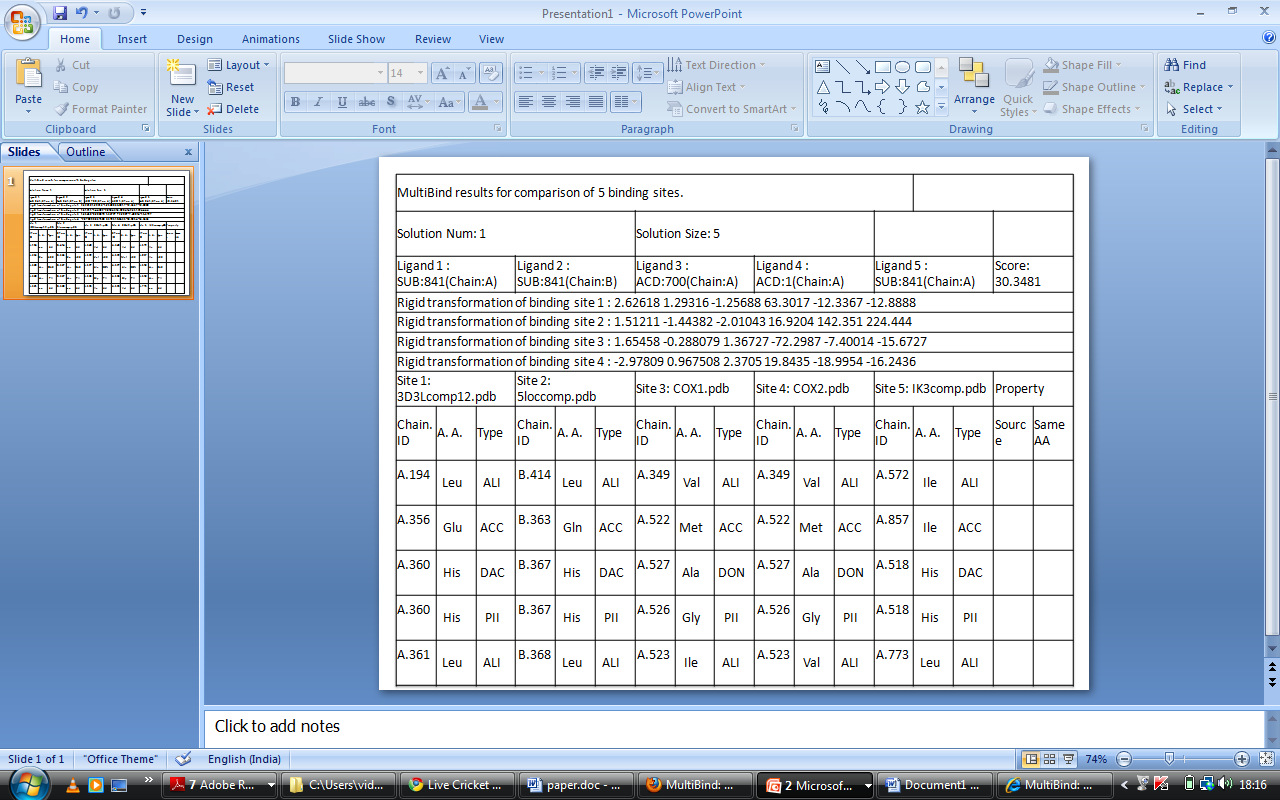


**19) 15LOX-sLOX1-12LOX-5LOX-COX2**


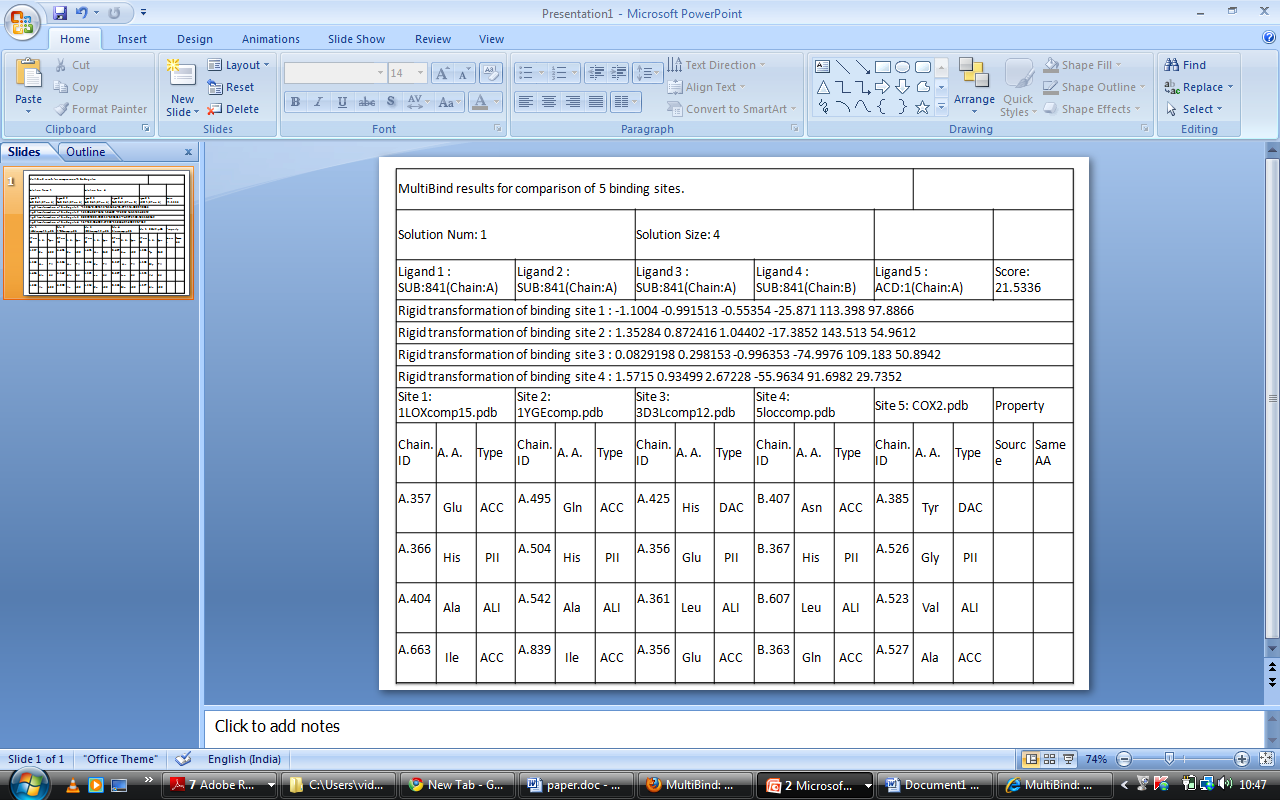


**20) 15LOX-12LOX-5LOX-COX1-COX2**


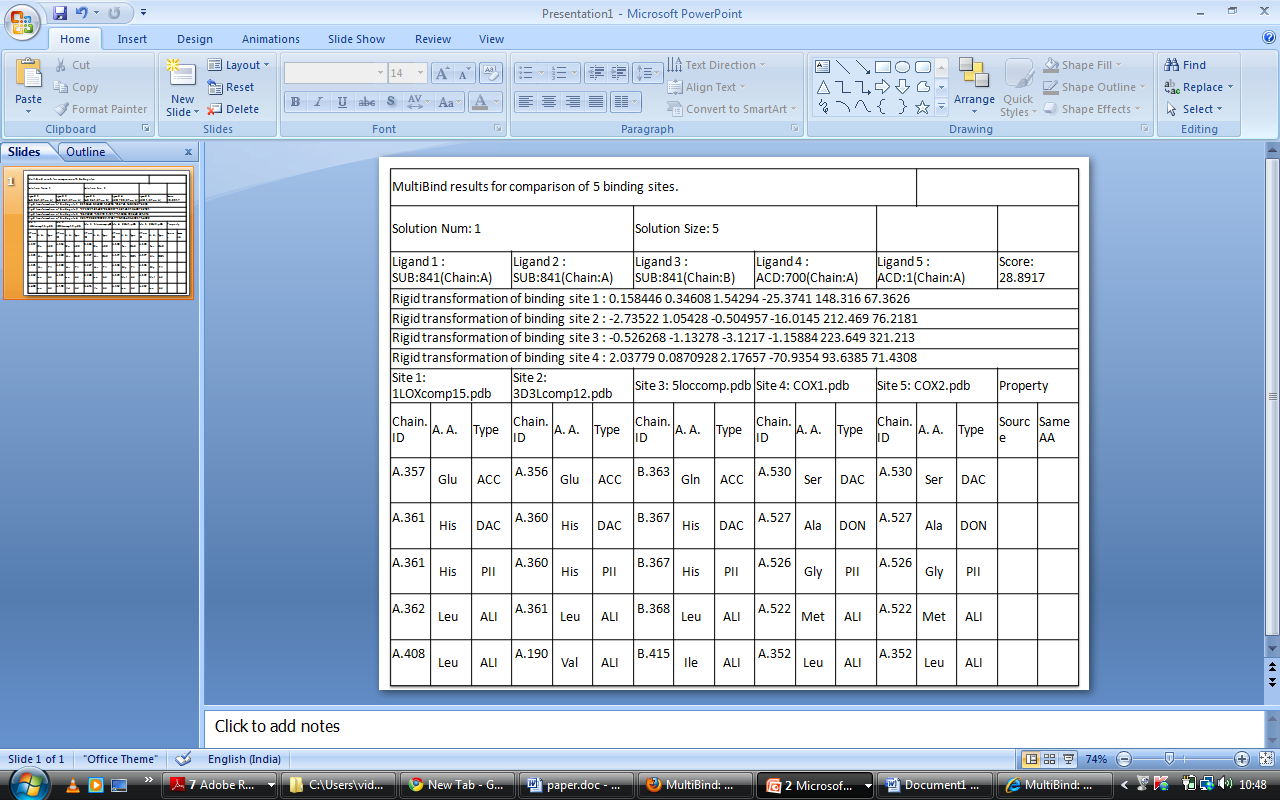


**21)sLOX1-12LOX-5LOX-COX1-COX2**


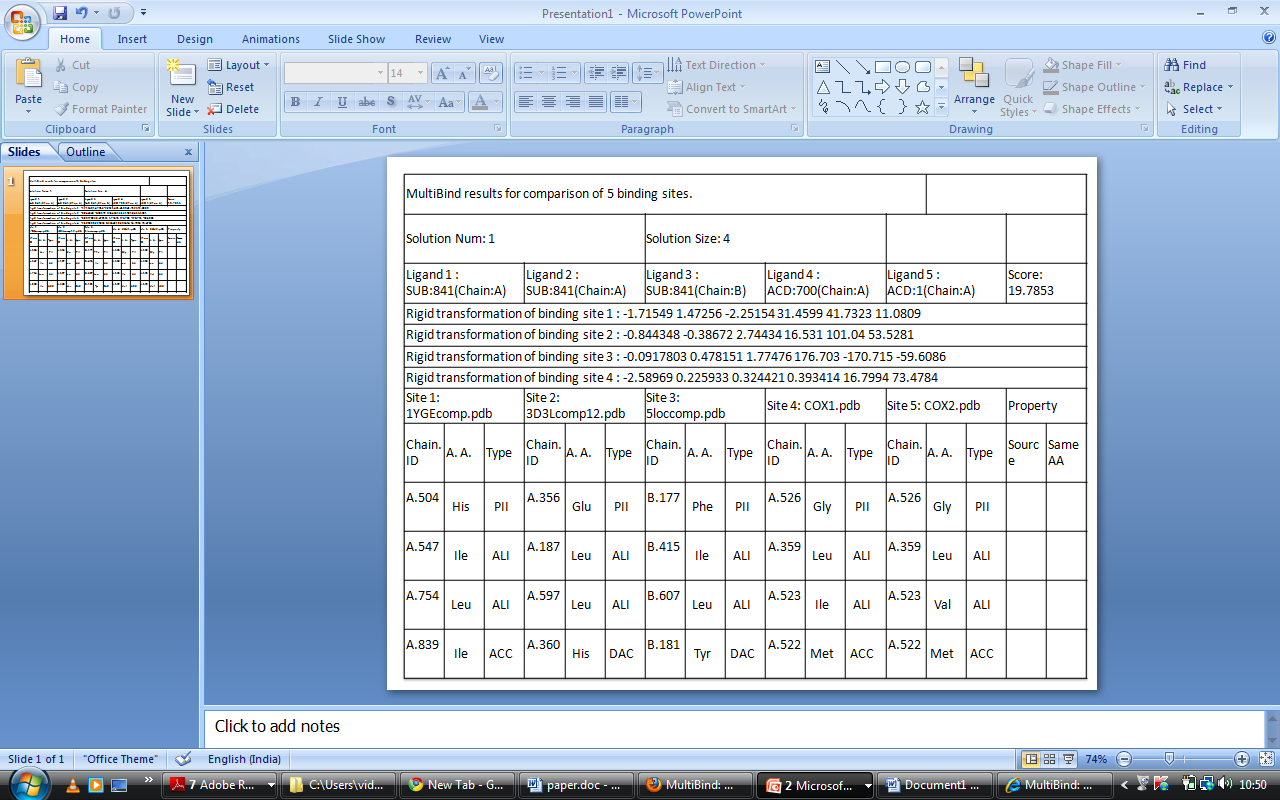


**MULTIPLE ALIGNMENTS OF 6 BINDING SITES**:

Binding sites were aligned, eliminating one synthase at a time from the MultiBind during run time. Comparison between six predicted binding sites of Arachidonic acid at a time revealed a pattern of between 4-5 common physicochemical properties.

| **COMPARED PROTEINS** | **NO. OF DETECTED FEATURES** | **SCORE** |
| --- | --- | --- |
| 1. 15LOX-sLOX1-12LOX-COX1-COX2-sLOX3 | 5 | 21.2547 |
| 1. 15LOX-12LOX-5LOX-COX1-COX2-sLOX3 | 5 | 26.0583 |
| 1. 15LOX-sLOX1-12LOX-5LOX-COX1-COX2 | 4 | 21.0095 |
| 1. 15LOX-sLOX1-12LOX-5LOX-COX1-sLOX3 | 4 | 19.9473 |
| 1. 15LOX-sLOX1-5LOX-COX1-COX2-sLOX3 | 4 | 20.5995 |
| 1. sLOX1-12LOX-5LOX-COX1-COX2-sLOX3 | 4 | 18.4751 |

1. **15LOX-sLOX1-12LOX-COX1-COX2-sLOX3**


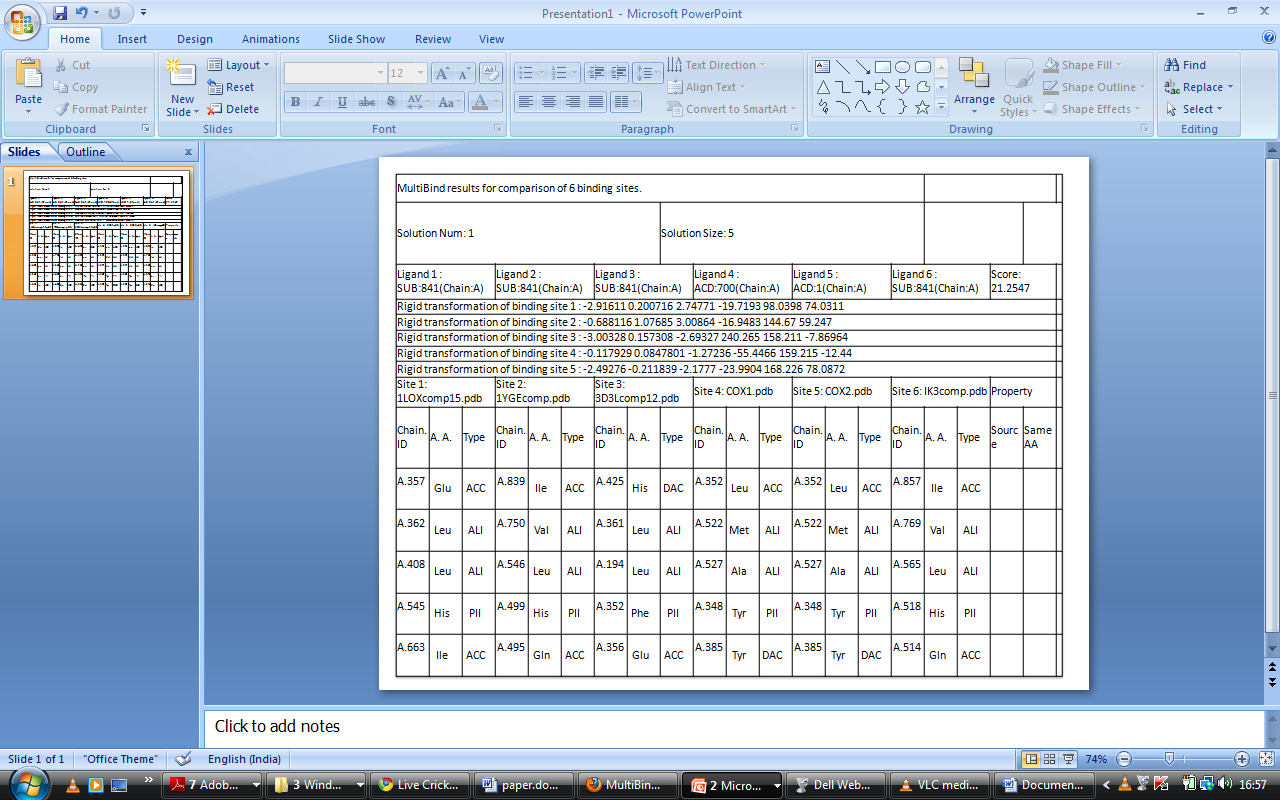


1. **15LOX-12LOX-5LOX-COX1-COX2-sLOX3**


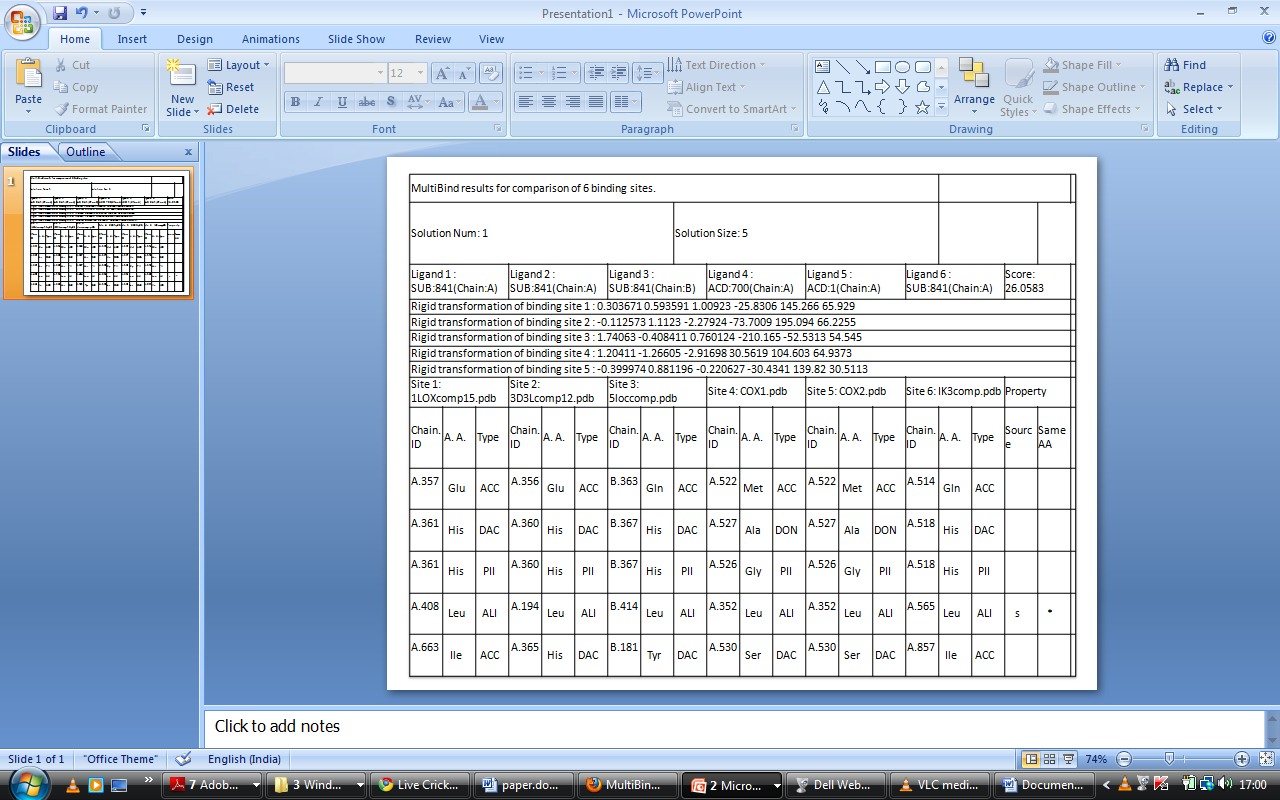


1. **15LOX-sLOX1-12LOX-5LOX-COX1-COX2**


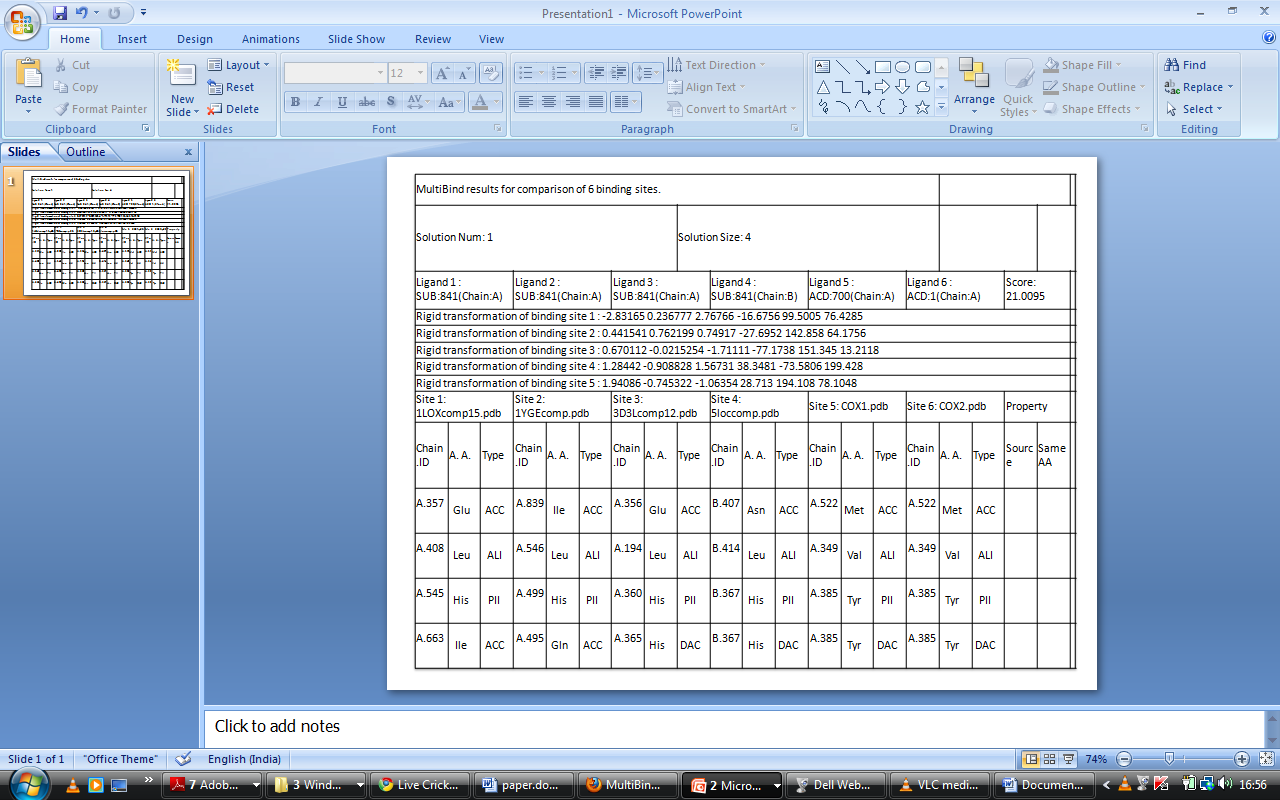


1. **15LOX-sLOX1-12LOX-5LOX-COX1-sLOX3**


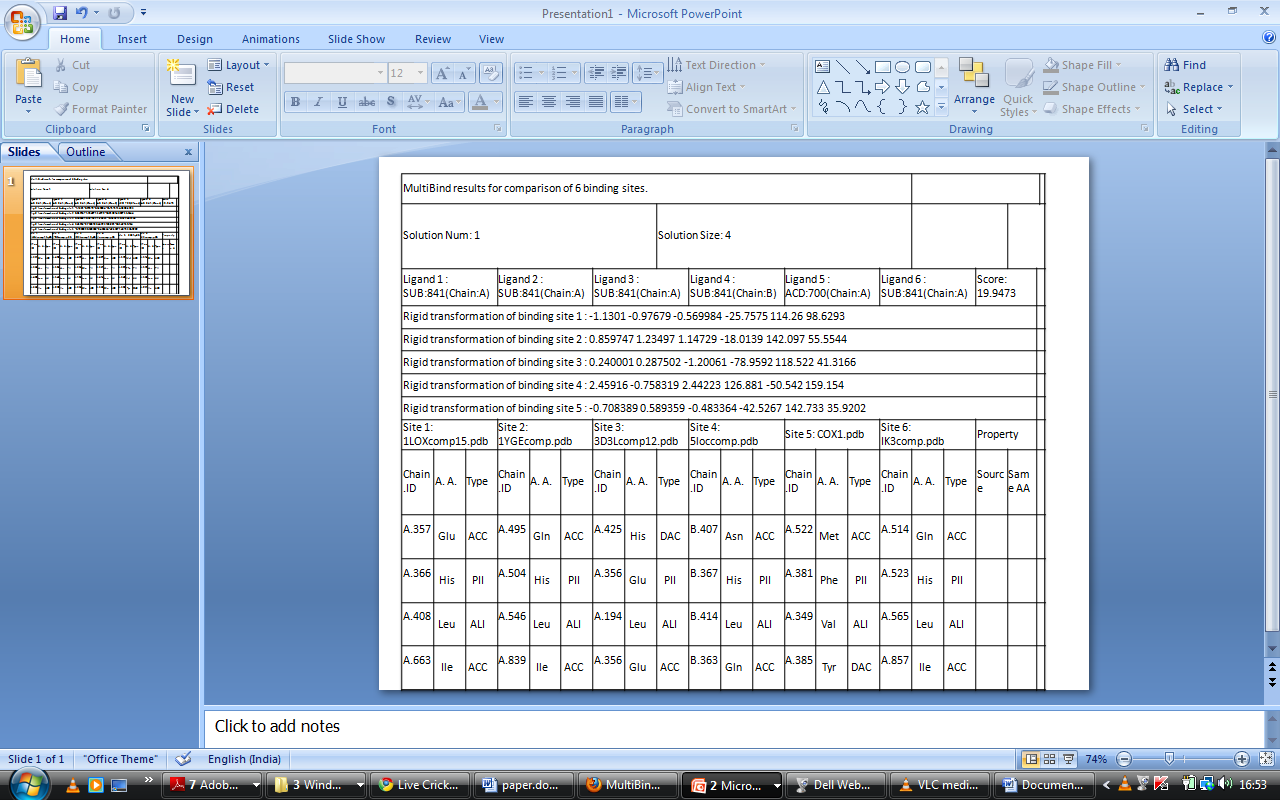


1. **15LOX-sLOX1-5LOX-COX1-COX2-sLOX3**


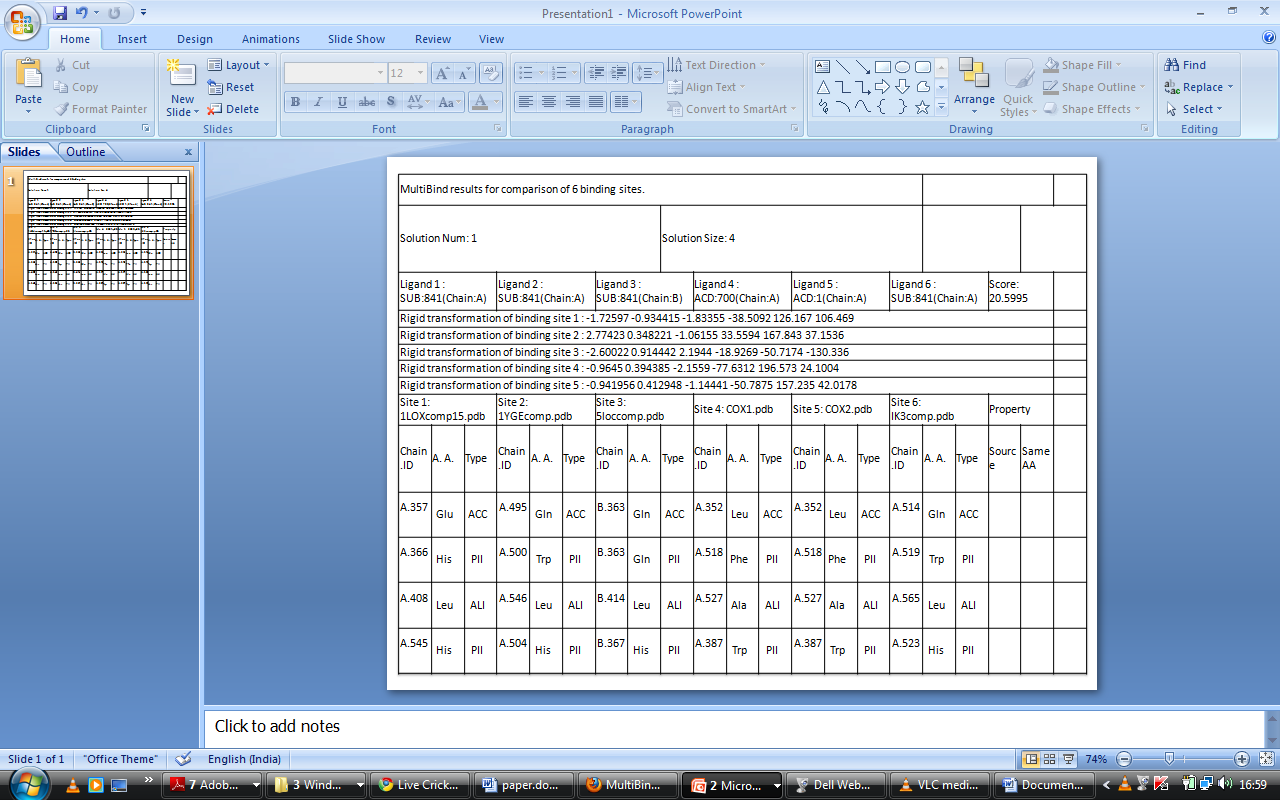


1. **sLOX1-12LOX-5LOX-COX1-COX2-sLOX3**


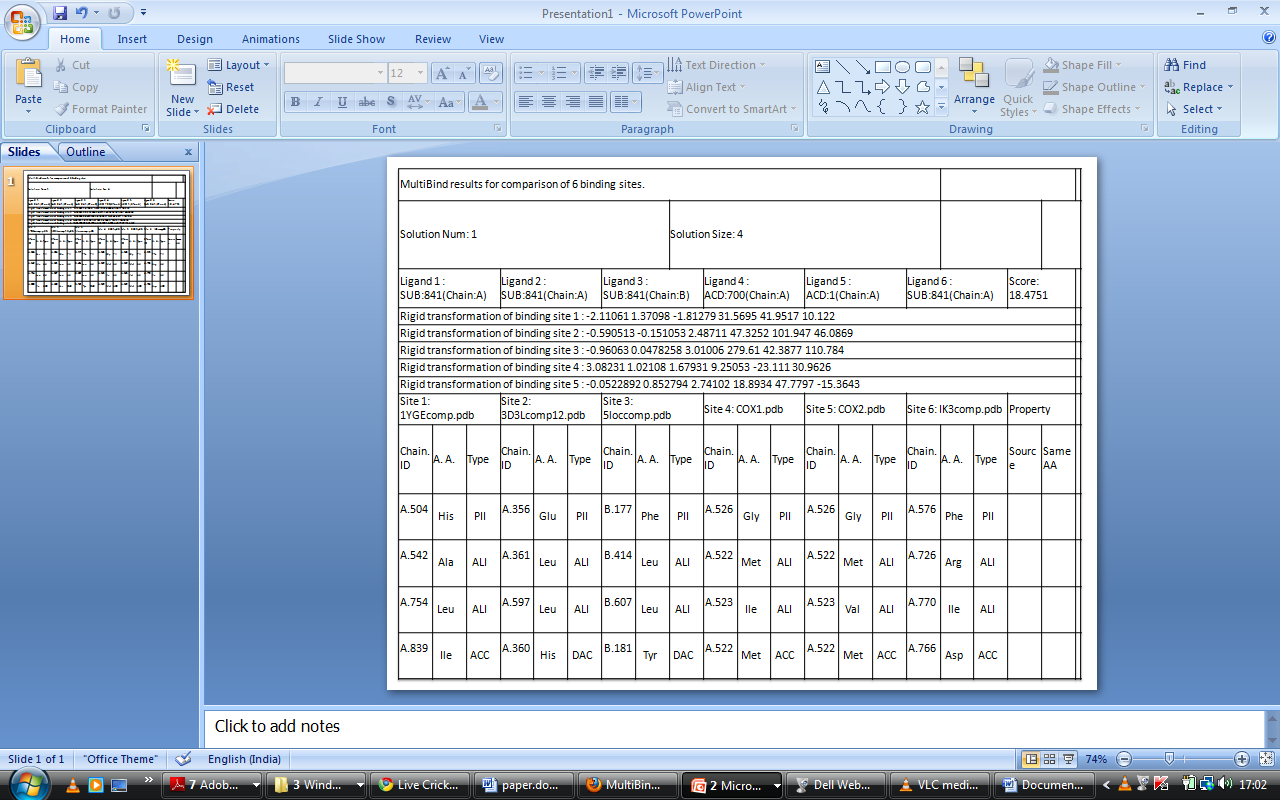

Supplement: Additional file 1: — Exploration of binding site pattern in arachidonic acid metabolizing enzymes, lipoxygenases and cycloxygenases. [file 13104_2015_1101_MOESM1_ESM.doc]
